# Analysis of Metabolic Differences Between Marselan and Cabernet Sauvignon Grape Berries from the Manas Wine-Producing Region of Xinjiang Based on Untargeted Metabolomics

**DOI:** 10.3390/foods15193534

**Published:** 2026-10-03

**Authors:** Guiyin Wei, Yun Wu, Na Xu, Jiawei Zhu, Ying Deng, Tiancong Zheng, Wenshu Huang, Weilin Chen, Wenrui Ma

**Affiliations:** College of Food Science and Pharmacy, Xinjiang Agricultural University, Urumqi 830052, China

**Keywords:** Marselan, Cabernet Sauvignon, Manas region, untargeted metabolomics, differential metabolites, flavonoids, phenylalanine metabolism

## Abstract

To characterize cultivar-specific metabolic differences between Marselan and Cabernet Sauvignon grapes cultivated in the Manas subregion on the northern foothills of the Tianshan Mountains in Xinjiang, China, mature berries from the two cultivars grown in adjacent plots within the same vineyard were analyzed, with three independent biological replicates per cultivar (*n* = 3). Untargeted metabolomics based on UHPLC–Q Exactive MS was used to compare the overall metabolic profiles of the two cultivars. PCA revealed clear separation between their global metabolic profiles in both positive- and negative-ion modes. A total of 236 candidate differential metabolites were identified using VIP > 1 and unadjusted *p* < 0.05, of which 34 further met FDR ≤ 0.05 and |log_2_FC| ≥ 1. After reviewing metabolite annotation evidence and grape berry metabolic context, 13 biologically interpretable features were selected for hierarchical clustering in the main text. KEGG analysis showed that flavonoid biosynthesis remained significantly enriched after multiple-testing correction, whereas phenylalanine metabolism and flavone and flavonol biosynthesis also showed distinct differential metabolite mapping patterns. Overall, Marselan and Cabernet Sauvignon exhibited distinct cultivar-associated metabolic phenotypes in mature berries, mainly involving phenylalanine and its downstream phenolic and flavonoid-related metabolic processes. Given the limited sample size and the risk of overfitting in supervised models, the conclusions were interpreted mainly on the basis of PCA, multiple-testing-corrected univariate statistics, and metabolite annotation evidence.

## 1. Introduction

Grapevine (*Vitis vinifera* L.) is a fruit crop of major economic importance worldwide. Grape berries contain diverse metabolites, including polyphenols, terpenoids, amino acids, and organic acids, which together influence important quality attributes of grapes and wines, including color, aroma, and mouthfeel [1,2,3]. Cabernet Sauvignon, a natural hybrid of Cabernet Franc and Sauvignon Blanc [4,5], originated in Bordeaux, France, and is widely cultivated as a red wine grape cultivar [6]. Marselan was bred by crossing Cabernet Sauvignon with Grenache Noir and is characterized by small berries, strong disease resistance, and deep berry color [7,8,9]. Owing to differences in genetic background and environmental responsiveness, the two cultivars differ in berry size, ripening dynamics, phenolic composition, and the volatile and sensory characteristics of their wines [10,11,12], suggesting distinct metabolic bases for berry quality formation.

The Manas wine-producing region of Xinjiang lies in the Manas River Basin at the northern foot of the Tianshan Mountains (approximately 44° N), along the southern margin of the Junggar Basin. Vineyards in this region are mainly distributed on gently sloping piedmont alluvial fans along riverbanks and across oasis plains [13], at elevations of 470–522 m. The region has a temperate continental arid/semi-arid climate [14,15], with abundant solar radiation and thermal resources. Annual sunshine duration is approximately 2550–3550 h, annual precipitation is only about 90–100 mm, and evapotranspiration during the April–September grape-growing season approaches 1000 mm [16]. Thus, evapotranspiration greatly exceeds precipitation. The region also has pronounced diurnal temperature variation and a frost-free period of approximately 150–172 days [17], conditions that favor the accumulation of sugars, pigments, and flavor compounds in wine grapes. The combination of abundant sunshine, low precipitation, and large diurnal temperature variation creates a grape-growing environment with distinctive regional terroir characteristics [15,18].

Environmental variables such as temperature, light, and water status can markedly influence the accumulation and transformation of sugars, organic acids, phenolic compounds, volatile aroma precursors, and other metabolites in grape berries, thereby influencing berry quality and wine style [19,20]. In recent years, increasing research attention has focused on the quality characteristics of different grape cultivars in the Manas region. Studies of red wine grape cultivars in Manas from 2021 to 2023 showed that cultivar genotype was the primary factor driving differences in wine flavonoid composition, whereas vintage significantly affected volatile aroma composition, particularly terpenes and C13-norisoprenoids, resulting in interannual variation in fruity, floral, and sweet sensory notes [21]. However, direct comparisons of intracellular metabolite profiles in mature Marselan and Cabernet Sauvignon berries grown under the same terroir conditions within the Manas subregion remain limited, and the metabolite basis and associated pathways underlying cultivar differences have not been systematically characterized.

Untargeted metabolomics coupled with liquid chromatography–high-resolution mass spectrometry (LC-HRMS) enables broad acquisition of metabolic features without predefining target analytes and supports characterization of complex metabolic phenotypes through statistical analysis and database annotation [22,23]. This approach has been widely applied to investigate grape berry metabolic composition and variation associated with cultivars and environmental factors. Hou et al. [1] analyzed 66 grape cultivars using LC-MS-based metabolomics and revealed extensive metabolic diversity and distinct cultivar-associated metabolic characteristics. Under identical microclimatic conditions, Martins et al. [24] compared different grape cultivars and found that amino acids, flavonoids, and metabolites associated with berry cuticular wax contributed to cultivar-specific metabolic phenotypes. Grape metabolic composition also shows substantial environmental plasticity because temperature, solar radiation, precipitation, and soil conditions across subregions can affect the accumulation of phenolic compounds, organic acids, and other metabolites [25]. Large-scale studies across multiple vineyards and vintages further demonstrate that cultivar, growing region, vintage, and cultivation conditions jointly shape grape metabolic fingerprints [26]. Therefore, untargeted metabolomics provides an effective approach for resolving metabolic differences among grape cultivars grown in the same vineyard or under similar cultivation conditions from a biochemical perspective.

Accordingly, mature Marselan and Cabernet Sauvignon grape berries from the Manas wine-producing region of Xinjiang were used as experimental materials. Untargeted metabolomics based on UHPLC–Q Exactive MS, together with unsupervised PCA, candidate differential metabolite screening, and Kyoto Encyclopedia of Genes and Genomes (KEGG) pathway analysis, was used to compare the overall metabolic profiles of the two cultivars and identify candidate metabolites and pathways associated with between-group differences. This study aimed to compare the observed metabolic accumulation differences between Marselan and Cabernet Sauvignon berries grown in the same production region under similar cultivation conditions, thereby providing a metabolic basis for evaluating their quality characteristics and regional utilization in the Manas region.

## 2. Materials and Methods

### 2.1. Experimental Materials and Sample Collection

The Marselan and Cabernet Sauvignon grapes used in this study were harvested from a vineyard in the Manas wine-producing region of the Xinjiang Uygur Autonomous Region, China. The two cultivars were sampled from adjacent plots within the same vineyard. In 2024, vines of both cultivars were 17 years old, trained using a single-trunk cordon system, grown in gravelly soil, and managed using identical cultivation practices. The vineyard was managed with drip irrigation; according to vineyard management records for 2024, irrigation was applied approximately once per month during the growing season (approximately every 30 days), at 450 m^3^·ha^−1^ per irrigation event. Sampling was conducted at ripening in September 2024, when the total sugar content of the berries reached 250 g/L. The hundred-berry weight and average cluster weight were 90 g and 100 g for Marselan, respectively, and 112.3 g and 204 g for Cabernet Sauvignon, respectively.

Three independent biological replicates were collected for each cultivar and assigned unique identifiers: Marselan samples were labeled MM-P1, MM-P2, and MM-P3, whereas Cabernet Sauvignon samples were labeled MC-P1, MC-P2, and MC-P3. For each biological replicate, 10 healthy, independently growing vines were selected within the same vineyard. Vines used for different biological replicates did not overlap and were distributed across different vineyard positions as far as possible to reduce local environmental sampling bias. During sampling, clusters with no disease, pest damage, or mechanical injury and with relatively uniform maturity were selected from different canopy positions and both sides of the canopy. Three clusters were randomly collected from each vine, giving 30 clusters per biological replicate. Mature, intact berries were then randomly sampled from the upper, middle, and lower portions of the clusters, with berry numbers balanced among positions as far as possible, giving 100 berries per biological replicate. The collected berries were pooled as one independent biological sample, immediately snap-frozen in liquid nitrogen, transported to the laboratory under low-temperature conditions, and stored at −80 °C. Before analysis, samples were thoroughly mixed and ground under low-temperature conditions for subsequent metabolomics analysis.

### 2.2. Main Reagents and Instruments

Methanol (CAS 67-56-1, purity ≥ 99.0%; Thermo Fisher Scientific, Waltham, MA, USA) was used for metabolite extraction, and 2-chloro-L-phenylalanine (CAS 103616-89-3, purity 98%; Aladdin, Shanghai, China) was added to the extraction solvent at 4 ppm as an internal standard to assist in monitoring consistency and stability during sample preparation and LC-MS analysis. Subsequent global metabolite relative abundances were normalized by total peak area rather than quantified absolutely using the internal standard peak area. Acetonitrile (≥99.9%; Thermo Fisher Scientific, Waltham, MA, USA), formic acid (LC-MS grade; TCI, Tokyo, Japan), ammonium formate (≥99.9%; Sigma-Aldrich, St. Louis, MO, USA), and ultrapure water produced using a Milli-Q system (Millipore, Burlington, MA, USA) were used to prepare the liquid chromatography mobile phases; all reagents met the specifications stated in their respective certificates of analysis.

Metabolomic profiling was performed using a Vanquish ultra-high-performance liquid chromatography system coupled to a Q Exactive high-resolution mass spectrometer (Thermo Fisher Scientific, USA). The system was fitted with an ACQUITY UPLC^®^ HSS T3 column (2.1 mm × 100 mm, 1.8 μm; Waters, Milford, MA, USA). Auxiliary equipment included an H1850-R refrigerated centrifuge (Xiangyi, Changsha, China), a BE-2600 vortex mixer (Qilinbeier, Haimen, China), an MB-96 tissue homogenizer (Zhejiang Meibi Instrument Co., Ltd., Pinghu, China), a KQ-800DE ultrasonic cleaner (Kunshan Ultrasonic Instruments Co., Ltd., Kunshan, China), and 0.22-μm PTFE membrane filters (Jinteng, Tianjin, China).

### 2.3. Sample Preparation and Metabolite Detection

#### 2.3.1. Sample Preparation

Following the untargeted metabolomics sample preparation procedure described by Chu et al. [27], with appropriate modifications for grape berry tissue, 50 mg of thoroughly ground grape sample was accurately weighed into a 2 mL centrifuge tube. A total of 600 μL of methanol extraction solution containing 4 ppm 2-chloro-L-phenylalanine was added, giving a solid-to-solvent ratio (*m*/*v*) of 1:12 (g:mL). After vortexing for 30 s to ensure complete dispersion, steel beads were added, and the samples were homogenized at 55 Hz for 60 s in a tissue grinder, followed by ultrasonic extraction at room temperature for 15 min. The extracts were centrifuged at 12,000 rpm and 4 °C for 10 min. The supernatants were collected, filtered through 0.22-μm PTFE membranes, transferred to LC injection vials, and subjected to LC-MS untargeted metabolomics analysis.

#### 2.3.2. Chromatographic Conditions

Chromatographic separation employed a Thermo Vanquish ultra-high-performance liquid chromatography system fitted with an ACQUITY UPLC^®^ HSS T3 column (2.1 mm × 100 mm, 1.8 μm; Waters, Milford, MA, USA). The column was maintained at 40 °C, the flow rate was 0.3 mL/min, and the injection volume was 2 μL. In positive-ion mode, mobile phase A was 0.1% formic acid in water and mobile phase B was 0.1% formic acid in acetonitrile. In negative-ion mode, mobile phase A was 5 mmol/L aqueous ammonium formate and mobile phase B was acetonitrile. The same gradient elution program was used in both ion modes: 0–1 min, 10% B; 1–5 min, 10–98% B; 5–6.5 min, 98% B; 6.5–6.6 min, 98–10% B; and 6.6–8 min, 10% B.

#### 2.3.3. Mass Spectrometric Conditions

Mass spectra were acquired using a Q Exactive high-resolution mass spectrometer equipped with an electrospray ionization (ESI) source. Full MS/dd-MS^2^ data were acquired in data-dependent acquisition (DDA) mode in both positive- and negative-ion modes.

The spray voltage was 3.50 kV in positive-ion mode and −2.50 kV in negative-ion mode. The sheath gas and auxiliary gas flow rates were 40 arb and 10 arb, respectively, and the capillary temperature was maintained at 325 °C. Full-scan MS1 spectra covered *m*/*z* 100–1000 at a resolution of 70,000 FWHM. In each acquisition cycle, the 10 most intense precursor ions were selected for higher-energy collisional dissociation (HCD) fragmentation. The normalized collision energy was set to 30 eV, MS/MS spectra were acquired at a resolution of 17,500 FWHM, and dynamic exclusion was set to automatic mode.

#### 2.3.4. Quality Assurance and Analytical Stability

To minimize technical variation during sample preparation and instrumental analysis, all six independent biological samples (three per cultivar) were prepared separately using the same pretreatment workflow described above, and each was analyzed once within the same LC-MS batch under identical instrumental conditions. 2-Chloro-L-phenylalanine was used as an exogenous internal standard to monitor within-batch consistency. Across the six biological sample injections, the retention time deviation of the internal standard was <2%, and its peak area coefficient of variation was <20%. The retention time shift of representative metabolites was <0.1 min. Blank samples showed no obvious contamination signals, and the chromatographic baseline remained stable.

### 2.4. Raw Data Processing and Metabolite Identification

Raw mass-spectrometric files were converted to mzXML using MSConvert in ProteoWizard (v3.0.8789). Peak detection, peak filtering, retention time correction, peak alignment, and peak area extraction were performed using the XCMS package (v3.12.0) in R. The main parameters were set as follows: method = “centWave”, bw = 2, ppm = 15, peakwidth = c(5, 30), mzwid = 0.015, and mzdiff = 0.01. A quantitative matrix of metabolic features was then generated. Total peak area normalization was subsequently applied to each sample by dividing the raw peak area of each retained metabolic feature by the sum of the peak areas of all retained metabolic features within the same sample. The resulting normalized values were used as relative abundances for subsequent statistical analyses.

Metabolite annotation was based on accurate mass and available MS/MS spectral information. Molecular formulas were predicted from precursor-ion *m*/*z* values, mass errors, and adduct forms, and candidate compounds were matched against HMDB, MassBank, LIPID MAPS, mzCloud, KEGG, and an in-house reference spectral library provided by the analytical service provider. The precursor-ion matching tolerance was <30 ppm. For metabolic features with acquired MS/MS spectra, experimental fragment ions were further compared with reference spectra. Only compounds for which authentic standards matched both retention time and MS/MS spectra under the same LC-MS conditions were considered structurally confirmed at the highest level; all other assignments were treated as putative annotations according to the strength of the available evidence. The complete metabolite table reports compound name, *m*/*z*, retention time (RT), ion mode, adduct ion, mass error, database/spectral library source, and the available identification/annotation level (Supplementary Table S2A).

### 2.5. Multivariate Statistical Analysis and Screening of Differential Metabolites

Statistical analyses were performed in R. Principal component analysis (PCA) was used for unsupervised evaluation of the overall sample distribution. Partial least squares-discriminant analysis (PLS-DA) and orthogonal partial least squares–discriminant analysis (OPLS-DA) were conducted using the ropls package (v1.22.0), and permutation tests were used to assess model-overfitting risk. Statistical analyses were based on six independent biological samples, with three replicates each for Marselan and Cabernet Sauvignon (*n* = 3). Univariate differences between the two cultivars were evaluated using Student’s *t*-test. Candidate differential metabolites were initially screened using VIP > 1 and unadjusted *p* < 0.05, followed by Benjamini–Hochberg multiple testing correction. Among the 236 candidate differential metabolites, 34 metabolic features that additionally met FDR ≤ 0.05 and |log_2_FC| ≥ 1 were included in a stricter statistical screening set. This statistical filtering reflects the strength of the between-group difference and does not represent confidence in compound structural identification; annotation evidence was evaluated separately using accurate mass, MS/MS information, spectral library matching, and authentic standard verification. Fold change (FC) was defined for MC-P relative to MM-P.

### 2.6. KEGG Pathway Enrichment Analysis

To elucidate the major biological processes associated with differential metabolites in Marselan and Cabernet Sauvignon grape berries, the workflow described by Pang et al. [28] was used as a reference. The 236 candidate differential metabolites obtained using VIP > 1 and unadjusted *p* < 0.05 were used as the input set for KEGG pathway analysis, and metabolites successfully mapped to KEGG were included in subsequent enrichment and topology analyses. *p* values were adjusted for multiple testing using the Benjamini–Hochberg method to obtain FDR values. Pathway topology analysis used betweenness centrality to evaluate node contribution, and KEGG Mapper was used to visualize metabolite–pathway relationships. The differential abundance score (DA score), defined as (Up_hits − Down_hits), was used to describe the overall direction of change in candidate differential metabolites within each pathway.

## 3. Results

### 3.1. Quality Assessment of LC-MS Analysis

**Figure 1:**
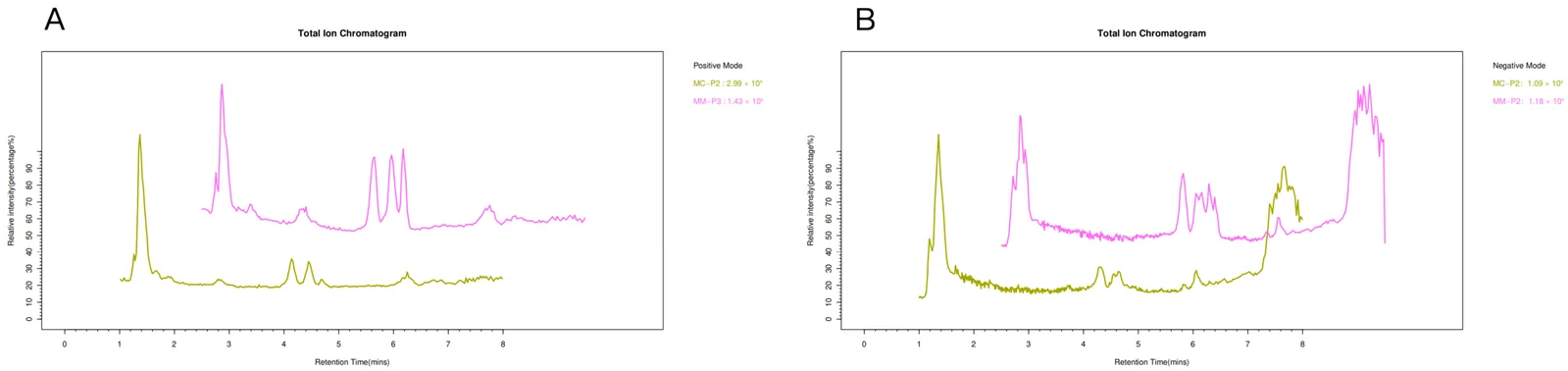
LC-MS chromatographic response profiles of Marselan and Cabernet Sauvignon grape berries in positive- and negative-ion modes. (**A**) Positive-ion mode; (**B**) negative-ion mode.

To illustrate the overall chromatographic response in untargeted metabolomics analysis, representative total ion current (TIC) chromatograms of Marselan and Cabernet Sauvignon grape berry samples acquired in positive and negative ionization modes are presented, respectively. As shown in Figure 1, abundant chromatographic responses were detected in both ionization modes, indicating that a relatively rich set of ion features can be effectively obtained under the adopted chromatography-mass spectrometry conditions. The two cultivars exhibit similar overall chromatographic profiles in partial retention time intervals, but differences are observed in the response intensity of some chromatographic peaks. The aforementioned representative TICs confirm that good chromatographic separation and signal response were achieved throughout the analysis, which provides a data foundation for subsequent metabolite identification and comparative analysis between the two cultivars.

After peak extraction, retention time correction, peak alignment, normalization, and database matching, 1291 metabolite annotations were obtained from the LC-MS data. Table 1 summarizes the biological sample size, metabolite annotation counts, and differential metabolite screening results. Identification and annotation information for the 236 candidate differential metabolites used in subsequent differential analysis is provided in Supplementary Table S2A, and their relative abundances and statistical results across the six biological samples are provided in Supplementary Table S2B; the distribution of annotation levels for the candidate differential metabolites and the stricter statistical screening set is summarized in Supplementary Table S1. Because this study used untargeted relative quantification and no standard curves were established, the reported signal values represent relative abundance differences among samples.

### 3.2. Differences in Overall Metabolic Profiles Between Marselan and Cabernet Sauvignon Grape Berries

#### 3.2.1. Principal Component Analysis (PCA)

**Figure 2:**
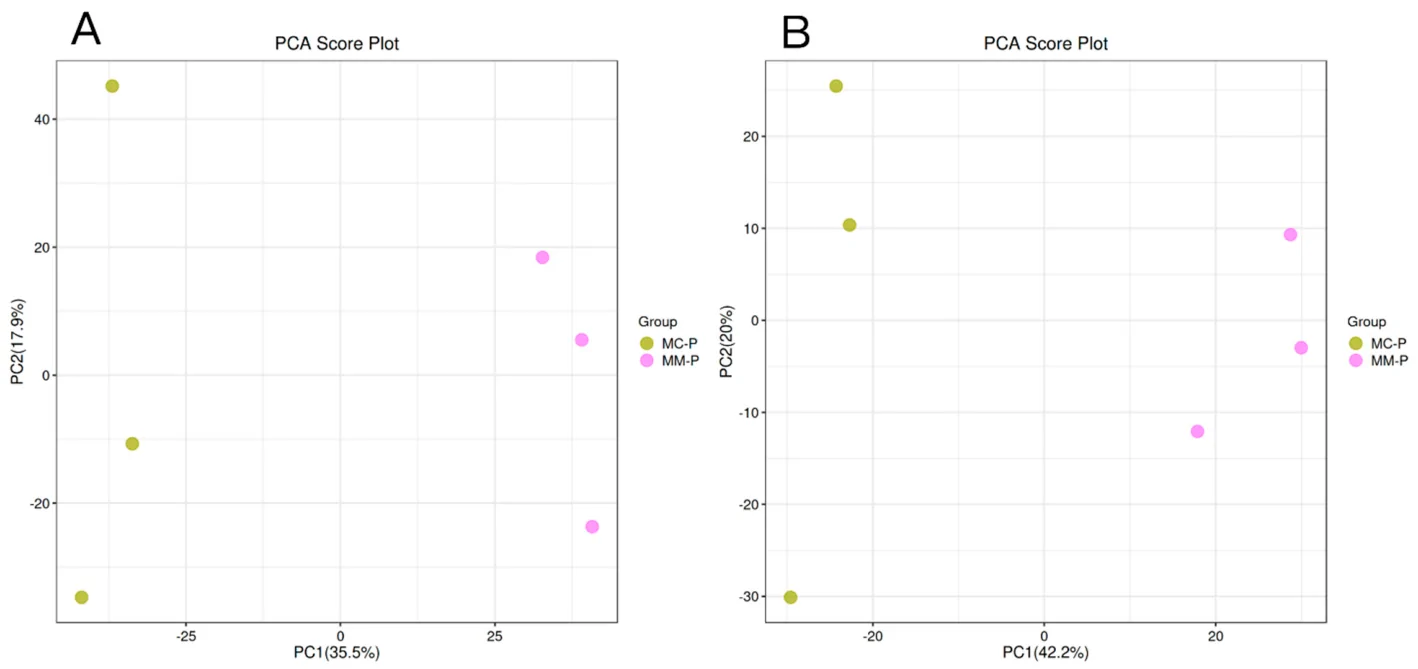
Principal component analysis (PCA) score plots of Marselan and Cabernet Sauvignon grape berries in positive- and negative-ion modes. Note: (**A**) Positive-ion mode; (**B**) negative-ion mode. Each cultivar included three independent biological replicates (*n* = 3).

To evaluate differences in the overall metabolic profiles of Marselan and Cabernet Sauvignon grape berries, principal component analysis (PCA) was conducted separately for the positive- and negative-ion datasets. As shown in Figure 2, PC1 and PC2 explained 35.5% and 17.9% of the variance, respectively, in positive-ion mode, for a cumulative explained variance of 53.4%. In negative-ion mode, PC1 and PC2 explained 42.2% and 20.0% of the variance, respectively, for a cumulative explained variance of 62.2%. In both ion modes, Marselan and Cabernet Sauvignon samples were clearly separated, mainly along PC1, and biological replicates generally clustered within their respective groups. Although some within-group dispersion was observed along PC2, the overall between-group separation remained clear. As an unsupervised method, PCA demonstrated distinct overall metabolic profiles between mature berries of the two cultivars, providing a basis for subsequent differential metabolite screening and metabolic pathway analysis. The corresponding PCA loading plots are provided in Supplementary Figure S1 to show the distribution of variable loadings in principal component space and assist in identifying metabolic features contributing to sample separation.

#### 3.2.2. PLS-DA Analysis

PLS-DA was initially used to explore sample separation trends and variable contributions under supervised grouping conditions. Given that each group contained only three biological replicates (*n* = 3), R^2^Y = 1, and the permutation test Q^2^ intercept remained above 0, the model showed a clear risk of overfitting. Therefore, PLS-DA was not used as primary evidence in the main text; its score plots and permutation tests were moved to the Supplementary Materials (Supplementary Figure S2) and are shown only for exploratory purposes.

#### 3.2.3. OPLS-DA Analysis and Permutation Test

**Figure 3:**
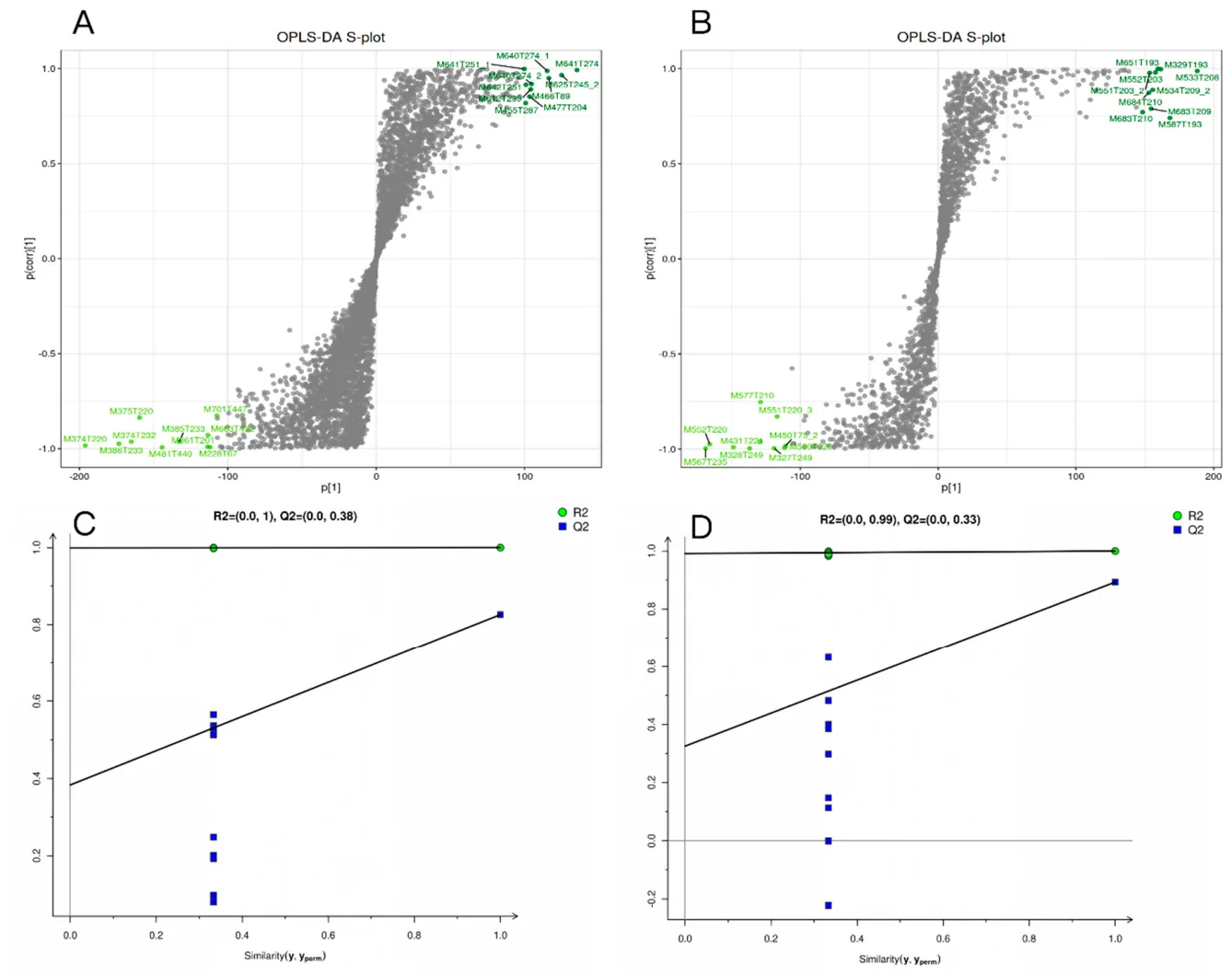
Orthogonal partial least squares–discriminant analysis (OPLS-DA) S-plots and model permutation tests for Marselan and Cabernet Sauvignon grape berries in positive- and negative-ion modes. Note: (**A**) OPLS-DA S-plot in positive-ion mode; (**B**) OPLS-DA S-plot in negative-ion mode; (**C**) permutation test of the positive-ion OPLS-DA model; (**D**) permutation test of the negative-ion OPLS-DA model.

To further identify metabolic features contributing strongly to the differences between Marselan and Cabernet Sauvignon grape berries, S-plots for positive- and negative-ion modes (Figure 3A,B) were constructed from the OPLS-DA models. Metabolic features in both ion modes showed a typical S-shaped distribution. Variables located at the extremes of the plots and far from the origin showed high covariance and correlation with class separation and, therefore, represented the main candidate differential features driving separation between the two metabolic profiles. Variables at the upper-right and lower-left ends of the S-plots were associated with the two cultivar groups in opposite directions, indicating that the metabolic differences were not driven by a single metabolite but involved covariation among multiple metabolic features with distinct accumulation patterns. Permutation tests were further performed to evaluate the robustness of the OPLS-DA models (Figure 3C,D). According to the regression plots, the intercepts of the R^2^ and Q^2^ regression lines at a permutation correlation coefficient of 0 were approximately 1.00 and 0.38, respectively, in positive-ion mode and approximately 0.99 and 0.33, respectively, in negative-ion mode. Although the Q^2^ values of the original models were higher than those of the permuted models, the positive Q^2^ intercepts indicate a risk of overfitting; therefore, the OPLS-DA results were used only for exploratory visualization of variable contributions.

The main multivariate statistical results and model validation parameters are summarized in Table 2. Feature-level univariate statistics are provided in Supplementary Tables S2B, S3 and S5.

### 3.3. Screening of Differential Metabolites Between Marselan and Cabernet Sauvignon

#### 3.3.1. Volcano Plot Analysis of Differential Metabolites

**Figure 4:**
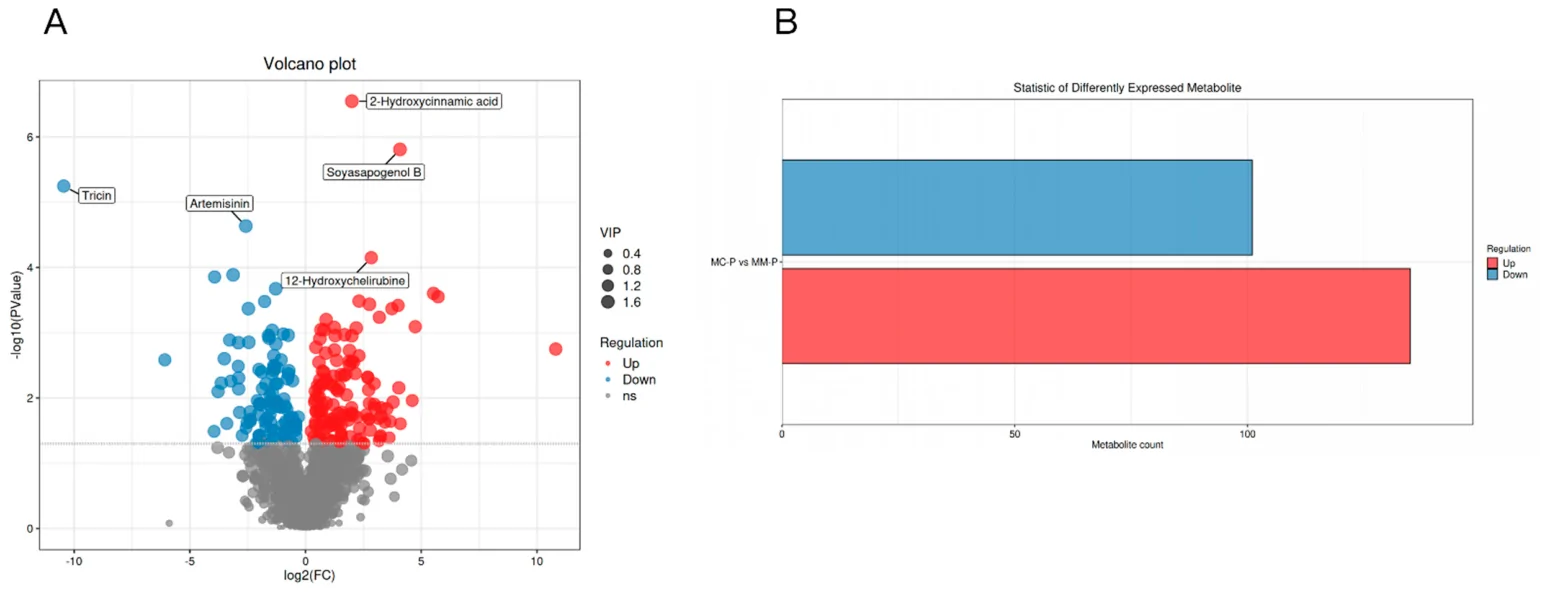
Volcano plot and numerical summary of differential metabolites in Marselan and Cabernet Sauvignon grape berries. Note: (**A**) Volcano plot of differential metabolites; (**B**) number of differential metabolites. Red indicates metabolites with increased abundance, blue indicates metabolites with decreased abundance, and gray indicates metabolites without significant differences; bubble size represents the VIP value.

To characterize differential metabolism between Marselan and Cabernet Sauvignon grape berries, 236 candidate differential metabolites were identified using VIP > 1 and *p* < 0.05. Of these, 135 showed higher relative abundance, and 101 showed lower relative abundance, accounting for 57.2% and 42.8%, respectively (Figure 4). Complete identification and annotation information for the candidate differential metabolites is provided in Supplementary Table S2A, and their relative abundances and statistical results are provided in Supplementary Table S2B. Using Cabernet Sauvignon relative to Marselan (MC-P vs. MM-P) as the comparison direction, phenolic acid-related compounds, such as 2-hydroxycinnamic acid, showed higher relative abundance in Cabernet Sauvignon, whereas tricin, trans-p-coumaric acid 4-glucoside, and L-phenylalanine showed higher relative abundance in Marselan. These candidate metabolites are mainly associated with aromatic amino acid, phenylpropanoid, and flavonoid-related secondary metabolic processes, indicating that under the present sampling conditions the two cultivars showed changes in opposite directions at multiple related metabolic nodes.

#### 3.3.2. Hierarchical Clustering Analysis of High-Confidence Differential Metabolites

**Figure 5:**
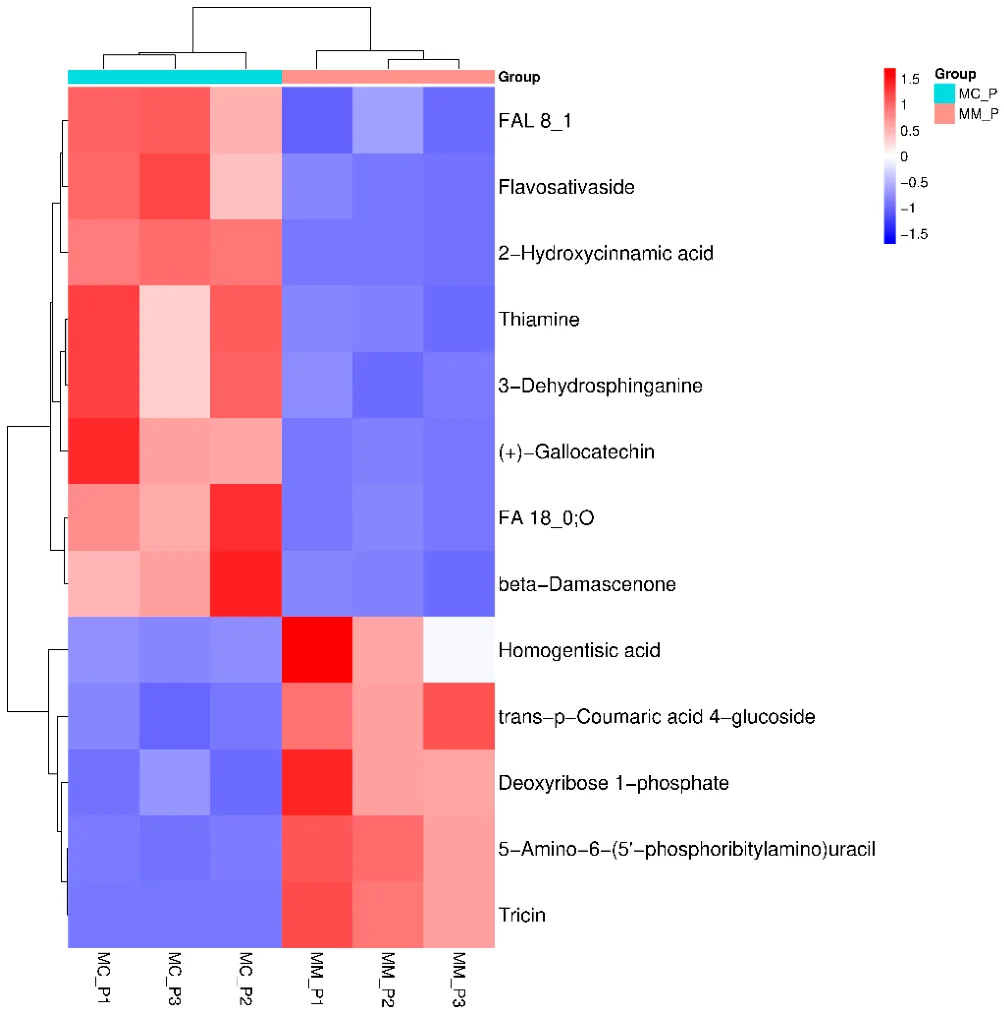
Hierarchical clustering heatmap of differential metabolites retained after annotation quality review in Marselan and Cabernet Sauvignon grape berries. Note: Relative abundances of individual metabolic features were standardized by row Z-scores; red indicates relatively high abundance and blue indicates relatively low abundance. Both samples and metabolic features were subjected to two-way hierarchical clustering using Euclidean distance and complete linkage. MC-P, Cabernet Sauvignon; MM-P, Marselan; each group contained three independent biological replicates (*n* = 3).

To further characterize differential metabolite accumulation, 236 candidate metabolites were initially screened using VIP > 1 and raw *p* < 0.05, of which 34 additionally met FDR ≤ 0.05 and |log_2_FC| ≥ 1. After further review of annotation evidence, KEGG pathway relevance, and grape berry metabolic context, 13 representative features were selected for hierarchical clustering in the main text (Figure 5). The complete set of 34 strictly filtered features is provided in Supplementary Table S3, and the 13 reviewed features displayed in Figure 5 are listed in Supplementary Table S4. Relative abundances were row-standardized as Z-scores, and two-way hierarchical clustering was performed using Euclidean distance and complete linkage. MC-P1–MC-P3 and MM-P1–MM-P3 formed two main branches, indicating different overall accumulation patterns for the selected features in the two cultivars. Specifically, 2-hydroxycinnamic acid and (+)-gallocatechin showed higher relative abundance in Cabernet Sauvignon, whereas trans-p-coumaric acid 4-glucoside, tricin, and homogentisic acid showed higher relative abundance in Marselan. The opposite accumulation directions among different features indicate differential accumulation among phenolic-acid-, flavan-3-ol-, and other related metabolic branches rather than a uniform increase or decrease in all related metabolites.

#### 3.3.3. Relative Abundance Analysis of Key Differential Metabolites

**Figure 6:**
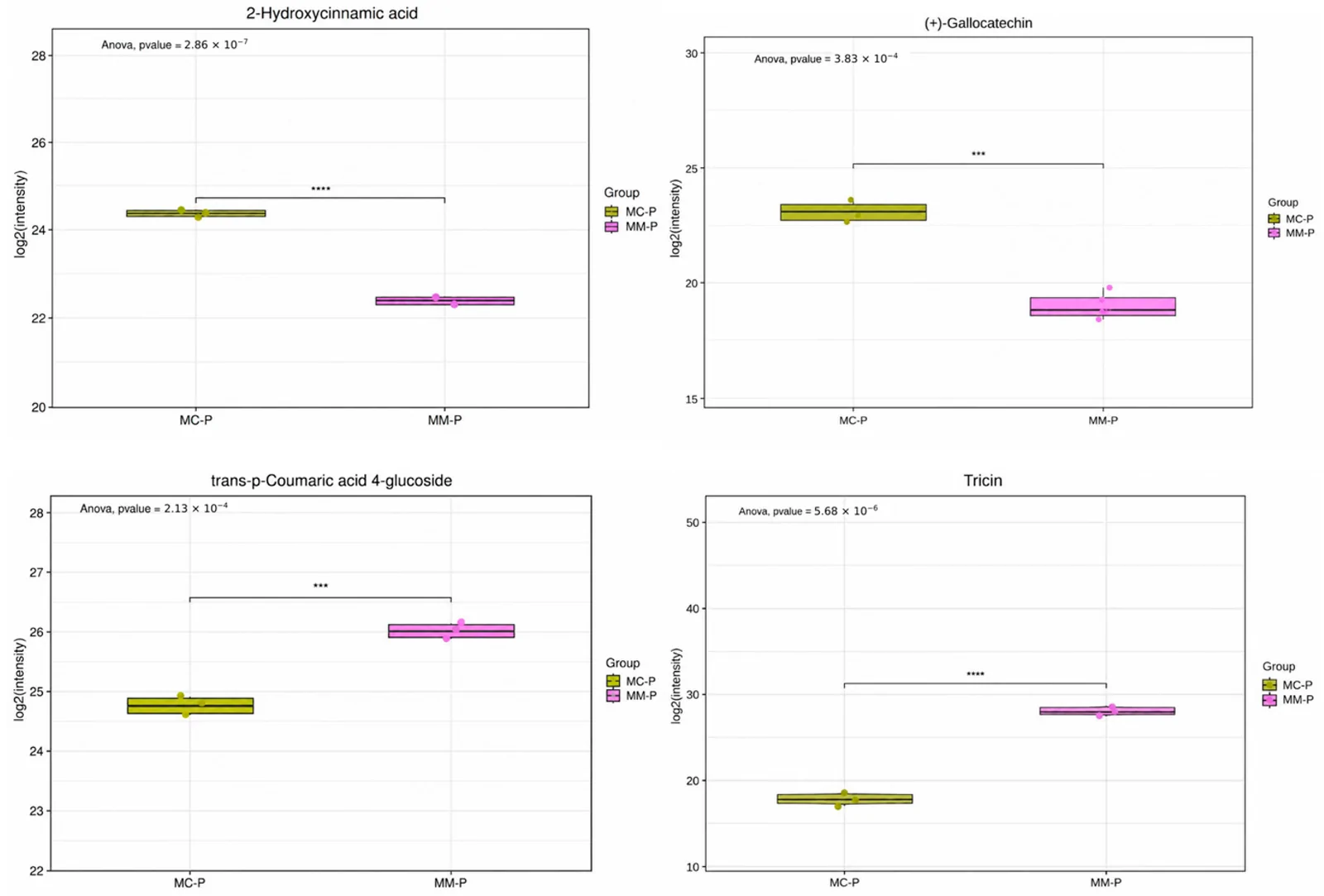
Comparison of the relative abundances of key differential metabolites in Marselan and Cabernet Sauvignon grape berries. Note: Statistical significance is indicated as follows: ***, *p* < 0.001; ****, *p* < 0.0001.

To further compare representative differential features associated with phenylpropanoid- and flavonoid-related metabolic processes in the two cultivars, four annotation-reviewed features—2-hydroxycinnamic acid, (+)-gallocatechin, trans-p-coumaric acid 4-glucoside, and tricin—were selected from the stricter statistical screening set for relative-abundance analysis (Figure 6). Statistical results and annotation information for these four representative features are provided in Supplementary Table S5. All four features showed clear and directionally consistent accumulation differences between the two cultivars.

2-Hydroxycinnamic acid showed an FC of 4.00 in Cabernet Sauvignon relative to Marselan (log_2_FC = 2.00, FDR = 0.00137), while (+)-gallocatechin showed an FC of 15.97 (log_2_FC = 4.00, FDR = 0.0145), indicating greater accumulation of hydroxycinnamic acid- and flavan-3-ol-related features in Cabernet Sauvignon. In contrast, trans-p-coumaric acid 4-glucoside showed higher relative abundance in Marselan (MC-P/MM-P: FC = 0.41, log_2_FC = −1.29, FDR = 0.0227), and the feature annotated as tricin was also more abundant in Marselan (FC = 0.0007, log_2_FC = −10.45, FDR = 0.00954).

From a metabolic perspective, 2-hydroxycinnamic acid and trans-p-coumaric acid 4-glucoside are both associated with hydroxycinnamic acid-related metabolism but accumulated in opposite directions in the two cultivars. Likewise, (+)-gallocatechin and tricin represent flavan-3-ol- and flavonoid-related features, respectively, and also showed opposite cultivar-associated trends. These responses across different downstream branches further support differential allocation of phenolic metabolism downstream of phenylalanine between Marselan and Cabernet Sauvignon.

### 3.4. KEGG Pathway Enrichment Analysis of Differential Metabolites

To further elucidate the principal metabolic processes associated with differential metabolic features in the two cultivars, KEGG pathway analysis was performed on the 236 candidate differential metabolites. Flavonoid biosynthesis remained significantly enriched after Benjamini–Hochberg correction and was the pathway with the clearest statistical evidence in this study. Phenylalanine metabolism and flavone and flavonol biosynthesis did not remain significant after correction but contained mapped differential metabolites and showed topological contributions and were, therefore, treated as supplementary evidence for related metabolic differences.

**Figure 7:**
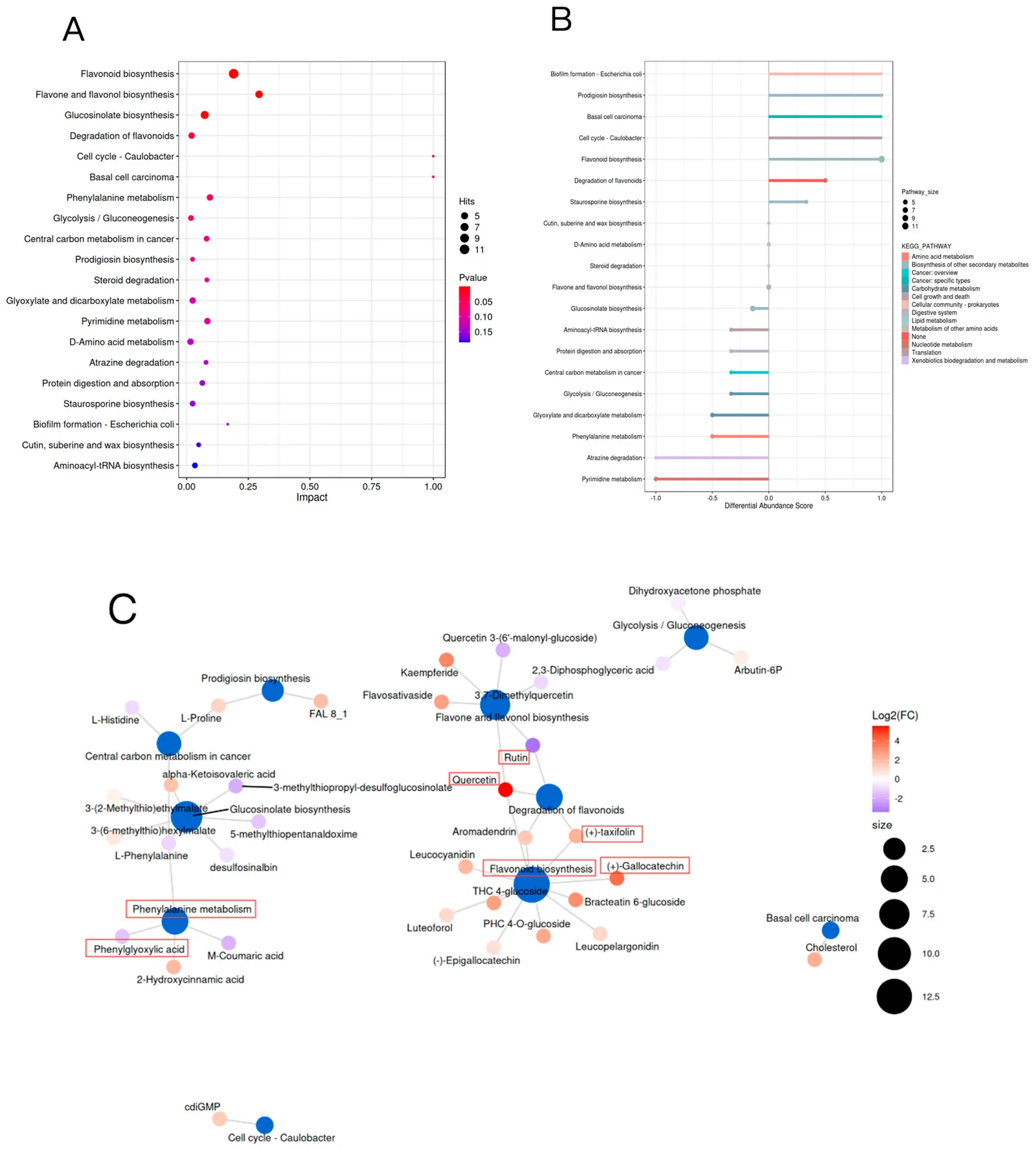
KEGG pathway topology analysis, differential abundance scores, and metabolite–pathway association network of differential metabolites in Marselan and Cabernet Sauvignon grape berries. (**A**) KEGG pathway topology analysis; bubble size indicates the number of differential metabolites mapped to each pathway, color indicates the *p* value, and the x-axis represents pathway impact. (**B**) KEGG pathway differential abundance score; positive values indicate that differential metabolites in the pathway tend to be relatively more abundant in Cabernet Sauvignon (MC-P), whereas negative values indicate relative enrichment in Marselan (MM-P). (**C**) Differential metabolite–KEGG pathway association network; metabolite node colors represent log_2_(FC) for MC-P relative to MM-P, with red indicating relative enrichment in MC-P and purple indicating relative enrichment in MM-P.

Differential abundance scoring further showed that the flavonoid biosynthesis pathway showed an overall positive score, indicating that annotated differential metabolites in this pathway generally tended to accumulate in Cabernet Sauvignon. In contrast, phenylalanine metabolism had a negative score, suggesting that related metabolites tended to accumulate more strongly in Marselan, whereas flavone and flavonol biosynthesis showed a clear bidirectional pattern (Figure 7B). These patterns indicate that the mapped metabolites in shared phenolic pathways changed in different directions between the two cultivars.

The metabolite–pathway association network further showed that 2-hydroxycinnamic acid, (+)-gallocatechin, and (+)-taxifolin had higher relative abundance in Cabernet Sauvignon, whereas L-phenylalanine, trans-p-coumaric acid 4-glucoside, quercetin 3-(6″-malonyl-glucoside), and rutin had higher relative abundance in Marselan (Figure 7C). Together with the hierarchical clustering and representative feature analyses, these results indicate that the between-cultivar differences were not characterized by synchronized changes in all phenylalanine- and flavonoid-related metabolites but rather by differential accumulation across phenolic acid-, flavan-3-ol-, and flavonoid-related branches.

In summary, flavonoid biosynthesis was the major pathway that remained significantly enriched after multiple-testing correction, whereas phenylalanine metabolism and flavone and flavonol biosynthesis provided supplementary evidence for phenolic metabolic differentiation between Marselan and Cabernet Sauvignon.

## 4. Discussion

The metabolic composition of grape berries is jointly shaped by cultivar genetic background and growing environment. In this study, Marselan and Cabernet Sauvignon were grown in adjacent plots within the same vineyard, with the same vine age, irrigation regime, and cultivation practices, and all samples were analyzed by liquid chromatography–mass spectrometry (LC-MS) in the same batch. This design reduced, to some extent, potential interference from cultivation management and analytical batch. However, because the study involved adjacent plots in a single vineyard without independent plot-level replication, contributions from local soil microenvironment, canopy light exposure, berry position, yield, and phenological status cannot be fully excluded. PCA showed separation of the overall metabolic profiles of the two cultivars in both positive- and negative-ion modes. Univariate analysis identified 236 candidate differential metabolites, of which 34 met the stricter criteria of FDR ≤ 0.05 and |log_2_FC| ≥ 1. After review of annotation quality and grape metabolic context, 13 biologically interpretable features were selected for hierarchical clustering in the main text.

These findings are generally consistent with the strong cultivar effects reported in previous studies. Hou et al. [1] conducted metabolomic analysis of 66 grape cultivars and revealed extensive metabolic diversity among grape genetic resources. Under the same microclimate at the Quinta de S. Luiz vineyard in the Douro Demarcated Region of northern Portugal, Martins et al. [24] compared three cultivars—Sousão, Touriga Nacional, and Viosinho—and found that amino acids, flavonoids, and cuticular wax-related metabolites contributed to cultivar-specific metabolic phenotypes. Lu et al. [29] further showed, through integrated metabolomic and transcriptomic analyses, that grape cultivars differ broadly across multiple branches of flavonoid metabolism. A three-year study (2021–2023) of nine red wine grape cultivars in the Manas region—Cabernet Sauvignon, Cabernet Franc, Marselan, Merlot, Malbec, Syrah, Petit Verdot, Dornfelder, and Pinot Noir—also showed that cultivar genotype is an important determinant of wine flavonoid composition, further supporting the possibility of persistent cultivar-associated chemical phenotypes under Manas conditions [21].

However, grape metabolic phenotypes also show substantial environmental plasticity, and factors such as light, temperature, water status, and soil conditions can regulate the accumulation of phenolic compounds, flavonoids, organic acids, and other metabolites [19,20,25]. Recent studies have shown that even within the same wine region, differences in solar radiation and edaphoclimatic conditions among subregions can produce distinct grape berry metabolic phenotypes, while water status, canopy structure, and diurnal temperature variation can also affect berry biochemical composition [30,31]. Therefore, the differences observed in this study are more appropriately interpreted as cultivar-associated metabolic phenotypic differences under the same regional background and similar cultivation conditions; the observed metabolic changes should not be attributed solely to genetic factors.

Based on the differential metabolites obtained through more stringent statistical screening combined with the results of KEGG pathway analysis, it can be observed that the metabolic differences between the two grape varieties are mainly involved in phenylalanine metabolism and the downstream phenylpropane and flavonoid-related metabolic processes. The relative accumulation levels of 2-hydroxycinnamic acid and (+)-gallocatechin are higher in Cabernet Sauvignon, while the metabolic characteristics of trans-p-coumaric acid 4-glucoside and tricin are relatively enriched in Marselan. The above-mentioned opposite accumulation trends indicate that the differences between the two varieties are not manifested as a consistent overall increase or decrease in related metabolites, but more likely reflect the differential partitioning among different branches downstream of phenolic secondary metabolism, demonstrating certain variety-specific regulatory characteristics of secondary metabolism. These results show that phenylalanine-derived phenolic acids, flavan-3-ols, and modified flavonoid-related metabolites accumulated in different directions between the two cultivars. Although pooled QC samples were not analyzed in this study, several methodological safeguards support the accuracy and stability of the replicate data. Specifically, the addition of 2-chloro-L-phenylalanine (4 ppm) as an internal standard throughout the extraction procedure allowed the monitoring of potential sample preparation and injection variations, while the use of a high-resolution Q Exactive mass spectrometer under identical, carefully controlled chromatographic and MS acquisition parameters minimized technical variability across injections. Accordingly, the representative total ion chromatograms of Marselan and Cabernet Sauvignon samples showed adequate chromatographic separation and signal response, providing a basis for subsequent comparative metabolomic analysis.

Previous studies have shown that, in addition to anthocyanins, different flavonoid subpathways also differ significantly among grape cultivars [29]. Integrated metabolomic and transcriptomic analysis has further shown that changes in the expression of key genes such as *C4H*, *CHS*, and *GST* during grape berry development are closely associated with the accumulation of different flavonoid metabolites [32]. Different red wine grape cultivars can also exhibit characteristic flavonoid profiles, and flavonols, hydroxycinnamic acids, and anthocyanin derivatives are important metabolites for distinguishing cultivar identity [33,34]. In addition, light exposure, source–sink relationships, and exogenous treatments can reshape flavonoid- and phenylpropanoid-related metabolism in grape berries [30,35]. The present study demonstrates cultivar-associated differences in phenolic metabolites and related pathways in mature Marselan and Cabernet Sauvignon berries; however, changes in metabolite abundance alone are insufficient to elucidate their upstream regulatory mechanisms.

In addition to phenolic metabolism, differential metabolites, such as β-damascenone and homogentisic acid, suggest that the two cultivars may also differ in aroma-related and other quality-associated metabolic processes. β-Damascenone was relatively enriched in mature Cabernet Sauvignon berries. This compound is an important aroma-related norisoprenoid, and its accumulation is influenced by carotenoid-derived metabolism, light exposure, and the canopy microenvironment. Previous studies have shown that both cluster shading and canopy orientation can significantly affect the accumulation of volatile compounds, such as β-damascenone [36,37]. In this study, both Marselan and Cabernet Sauvignon vines were trained using a single-trunk cordon system and managed consistently under the same training system. Therefore, the difference in the relative abundance of β-damascenone between the two cultivars is unlikely to be explained by differences in the training system and may instead reflect cultivar-specific metabolic backgrounds and their interactions with the microenvironment.

Meanwhile, a comparative study of Marselan and Cabernet Sauvignon wines from five wine-producing regions in China—the Jiaodong Peninsula, Huaizhuo Basin, Loess Plateau, Eastern Foothill of Helan Mountain, and Xinjiang—found that terpenoids could effectively distinguish wines from the two cultivars and that Marselan wines showed more pronounced floral aroma characteristics [12]. Previous studies have mainly focused on the overall aroma characteristics of finished wines, whereas the present study analyzed metabolite composition in grape berries. Although β-damascenone showed higher relative abundance in Cabernet Sauvignon berries, a single aroma-related metabolite in grape berries cannot directly determine the overall aroma of the final wine. Grape-to-wine aroma formation involves multiple stages, including the release of aroma precursors, yeast metabolism, and fermentation-driven transformations, and the metabolite composition of the raw fruit can undergo further changes during processing. Studies of fruit processing have shown that processing formulation and treatment can substantially alter phenolic composition, total phenol content, antioxidant activity, and sensory attributes, indicating that the chemical composition of raw fruit does not correspond directly to final processed product quality [38]. Therefore, the metabolic phenotypic differences reported here apply only to mature grape berries. Future work should integrate transcriptomics, enzyme activity analysis, fermentation trials, and multi-vintage and multi-vineyard validation to clarify the regulatory basis of the key metabolic differences and their significance in wine quality formation.

## 5. Conclusions

This study showed that, under the sampling conditions of a single vineyard in the Manas region of Xinjiang, mature Marselan and Cabernet Sauvignon berries exhibited distinct metabolic phenotypes, with the main differences concentrated in phenolic acid- and flavonoid-related metabolism downstream of phenylalanine. 2-Hydroxycinnamic acid and (+)-gallocatechin showed higher relative abundance in Cabernet Sauvignon, whereas trans-p-coumaric acid 4-glucoside and the feature tentatively annotated as tricin showed higher relative abundance in Marselan. KEGG analysis further showed that flavonoid biosynthesis was the major pathway that remained significantly enriched after multiple-testing correction, suggesting that flavonoid-related metabolism may be an important feature distinguishing the chemical phenotypes of mature berries of the two cultivars. These findings provide a reference framework for understanding cultivar-dependent differences in grape berry metabolic composition in the Manas region and offer candidate metabolic markers that may be valuable for cultivar discrimination, vineyard management, and the assessment of winemaking potential.

Within the sampling and analytical scope of this study, 2-chloro-L-phenylalanine was used as an internal standard to monitor retention time and peak area consistency across the six biological sample injections, and all samples were analyzed in the same LC-MS batch under identical acquisition conditions. Nevertheless, the study was conducted in a single vineyard without independent plot replication, included only three biological replicates per cultivar, and did not incorporate pooled QC samples or repeated technical injections; therefore, the conclusions should be interpreted within the specific conditions of this study. Future validation across multiple vintages and vineyards, together with a more comprehensive quality control design, will be needed to confirm the cultivar-dependent metabolic features identified here and to evaluate their stability across growing environments.

## Figures and Tables

**Table 1:** Summary of sample size, metabolite annotation, and differential metabolite screening.

Data Level	Number	Definition/Description
Biological samples	6	MC-P1–P3 (Cabernet Sauvignon) and MM-P1–P3 (Marselan), *n* = 3 per cultivar.
Annotated metabolites	1291	Supplementary Materials primarily summarize candidate differential metabolites and subsequent screening sets; corresponding identifiers are given below.
Candidate differential metabolites	236	VIP ≥ 1 + unadjusted *p* < 0.05; MC-P relative to MM-P: 135 higher and 101 lower. Annotation-level summary: Supplementary Table S1; identification/annotation: Table S2A; relative abundance and statistics: Table S2B.
Stricter statistical screening set	34	Among the 236 candidates, features additionally meeting FDR ≤ 0.05 and |log_2_FC| ≥ 1; complete list and annotation levels: Supplementary Table S3.
Main-text heatmap subset	13	Selected from the 34 strictly filtered features after review of annotation evidence and grape metabolic context; the 13 heatmap features are listed in Supplementary Table S4.

**Table 2:** Summary of the main multivariate statistical results and permutation test parameters.

Analysis	Ion Mode	Key Statistics	Interpretation
PCA	Positive	PC1 = 35.5%; PC2 = 17.9%; cumulative explained variance = 53.4%	Descriptive unsupervised separation
PCA	Negative	PC1 = 42.2%; PC2 = 20.0%; cumulative explained variance = 62.2%	Descriptive unsupervised separation
PLS-DA	Positive	R^2^X = 0.511; R^2^Y = 1.000; Q^2^ = 0.893; permutation Q^2^ intercept = 0.75	Exploratory only; overfitting risk
PLS-DA	Negative	R^2^X = 0.585; R^2^Y = 1.000; Q^2^ = 0.937; permutation Q^2^ intercept = 0.56	Exploratory only; overfitting risk
OPLS-DA	Positive	Permutation R^2^ intercept = 1.00; permutation Q^2^ intercept = 0.38	Exploratory visualization only
OPLS-DA	Negative	Permutation R^2^ intercept =0.99; permutation Q^2^ intercept = 0.33	Exploratory visualization only

## Data Availability

The original contributions presented in this study are included in the article/Supplementary Materials. Further inquiries can be directed to the corresponding author.

## Supplementary Materials

The following supporting information can be downloaded at: https://www.mdpi.com/article/10.3390/foods15193534/s1, Figure S1: PCA loading plots and PLS-DA permutation tests of Marselan and Cabernet Sauvignon grape berry metabolomic profiles in positive- and negative-ion modes. (A) PCA loading plot in positive-ion mode; (B) PCA loading plot in negative-ion mode; (C) permutation test of the PLS-DA model in positive-ion mode; (D) permutation test of the PLS-DA model in negative-ion mode; Figure S2: PLS-DA score plots and permutation tests of Marselan and Cabernet Sauvignon grape berry metabolomic profiles in positive- and negative-ion modes. (A) PLS-DA score plot in positive-ion mode; (B) PLS-DA score plot in negative-ion mode; (C) permutation test of the PLS-DA model in positive-ion mode; (D) permutation test of the PLS-DA model in negative-ion mode; Table S1: Summary of the current annotation-level labels for candidate and stricter statistical feature sets; Table S2: (A) Identification and annotation information for the 236 candidate differential metabolites (VIP > 1 and raw *p* < 0.05); (B) Relative abundances and statistical results for the 236 candidate differential metabolites. Table S3: Metabolites meeting the stricter statistical criteria (FDR ≤ 0.05 and |log_2_FC| ≥ 1), with annotation-level information; Table S4: The 13 reviewed differential metabolite features displayed in the Figure 6 heatmap; Table S5: Representative differential metabolite features emphasized in Figure 7 and the main biological interpretation; Table S6: Statistical results of KEGG pathway enrichment and topology analysis.

## References

[1] Hou H., Li Y., Zhou S., Zhang R., Wang Y., Lei L., Yang C., Huang S., Xu H., Gao M. (2024). Compositional Analysis of Grape Berries: Mapping the Global Metabolism of Grapes. Foods.

[2] Xia E.-Q., Deng G.-F., Guo Y.-J., Li H.-B. (2010). Biological Activities of Polyphenols from Grapes. Int. J. Mol. Sci..

[3] Nassiri-Asl M., Hosseinzadeh H. (2016). Review of the Pharmacological Effects of *Vitis vinifera* (Grape) and Its Bioactive Constituents: An Update. Phytother. Res..

[4] Moroldo M., Paillard S., Marconi R., Legeai F., Canaguier A., Cruaud C., De Berardinis V., Guichard C., Brunaud V., Le Clainche I. (2008). A Physical Map of the Heterozygous Grapevine ‘Cabernet Sauvignon’ Allows Mapping Candidate Genes for Disease Resistance. BMC Plant Biol..

[5] Cochetel N., Vondras A.M., Figueroa-Balderas R., Liou J., Peluso P., Cantu D. (2025). Phased Epigenomics and Methylation Inheritance in a Historical *Vitis vinifera* Hybrid. Genome Biol..

[6] Ren Y., Sadeghnezhad E., Leng X., Pei D., Dong T., Zhang P., Gong P., Jia H., Fang J. (2023). Assessment of ‘Cabernet Sauvignon’ Grape Quality Half-Véraison to Maturity for Grapevines Grown in Different Regions. Int. J. Mol. Sci..

[7] Krasteva D., Ivanov Y., Chengolova Z., Godjevargova T. (2023). Antimicrobial Potential, Antioxidant Activity, and Phenolic Content of Grape Seed Extracts from Four Grape Varieties. Microorganisms.

[8] Yermolin D., Yermolina G., Gerber Y., Zadorozhnaya D., Kotolovets Z. (2020). Phenolic Complex of Red Wine Materials from Grapes Growing in the Crimea. E3S Web Conf..

[9] Wang J., Zhang X., Su L., Li H., Zhang L., Wei J. (2020). Global Warming Effects on Climate Zones for Wine Grape in Ningxia Region, China. Theor. Appl. Climatol..

[10] Shi P., Yue T., Ai L., Cheng Y., Meng J., Li M., Zhang Z. (2016). Phenolic Compound Profiles in Grape Skins of Cabernet Sauvignon, Merlot, Syrah and Marselan Cultivated in the Shacheng Area (China). S. Afr. J. Enol. Vitic..

[11] Zhang K., Yuan L., Li Q., Wang R., Zhang Z.-Z. (2019). Comparison of the Anthocyanins Composition of Five Wine-Making Grape Cultivars Cultivated in the Wujiaqu Area of Xinjiang, China. OENO One.

[12] Song X., Yang W., Qian X., Zhang X., Ling M., Yang L., Shi Y., Duan C., Lan Y. (2023). Comparison of Chemical and Sensory Profiles Between Cabernet Sauvignon and Marselan Dry Red Wines in China. Foods.

[13] Cheng W., Zhou C., Liu H., Zhang Y., Jiang Y., Zhang Y., Yao Y. (2006). The Oasis Expansion and Eco-Environment Change over the Last 50 Years in Manas River Valley, Xinjiang. Sci. China Ser. D Earth Sci..

[14] Cheng G., He Y.-N., Yue T.-X., Wang J., Zhang Z.-W. (2014). Effects of Climatic Conditions and Soil Properties on Cabernet Sauvignon Berry Growth and Anthocyanin Profiles. Molecules.

[15] Lu H.-C., Hu L., Liu Y., Cheng C.-F., Chen W., Li S.-D., He F., Duan C.-Q., Wang J. (2022). Reducing the Source/Sink Ratio of Grapevine to Face Global Warming in a Semi-Arid Climate: Effects on Volatile Composition of Cabernet Sauvignon Grapes and Wines. Food Chem. X.

[16] He L., Xu X.-Q., Wang Y., Chen W.-K., Sun R.-Z., Cheng G., Liu B., Chen W., Duan C.-Q., Wang J. (2020). Modulation of Volatile Compound Metabolome and Transcriptome in Grape Berries Exposed to Sunlight under Dry-Hot Climate. BMC Plant Biol..

[17] Zhang H., Ahmed Z., Han W., Sun G. (2024). Ecosystem Service Changes and Water Management in the Manas River Basin. Water.

[18] Gao X.-T., Li H.-Q., Wang Y., Peng W.-T., Chen W., Cai X.-D., Li S.-D., He F., Duan C.-Q., Wang J. (2019). Influence of the Harvest Date on Berry Compositions and Wine Profiles of *Vitis vinifera* L. cv. ‘Cabernet Sauvignon’ Under a Semiarid Continental Climate over Two Consecutive Years. Food Chem..

[19] Rienth M., Vigneron N., Darriet P., Sweetman C., Burbidge C., Bonghi C., Walker R.P., Famiani F., Castellarin S.D. (2021). Grape Berry Secondary Metabolites and Their Modulation by Abiotic Factors in a Climate Change Scenario—A Review. Front. Plant Sci..

[20] Hewitt S., Hernández-Montes E., Dhingra A., Keller M. (2023). Impact of Heat Stress, Water Stress, and Their Combined Effects on the Metabolism and Transcriptome of Grape Berries. Sci. Rep..

[21] Shi N., Zhao Y.-F., Lu H.-C., Tian M.-B., Li M.-Y., Deng J.-Y., Wei J.-Y., Chen A.-S., Cheng C.-F., Li S.-D. (2025). Flavoromics Analysis and Sensory Evaluation of Wines Produced from Nine Red Grape Cultivars in Manas Region, Xinjiang, China: Implications for Terroir Adaptation. Food Chem..

[22] Pang Z., Zhou G., Ewald J., Chang L., Hacariz O., Basu N., Xia J. (2022). Using MetaboAnalyst 5.0 for LC-HRMS Spectra Processing, Multi-Omics Integration and Covariate Adjustment of Global Metabolomics Data. Nat. Protoc..

[23] Chen L., Lu W., Wang L., Xing X., Chen Z., Teng X., Zeng X., Muscarella A.D., Shen Y., Cowan A. (2021). Metabolite Discovery Through Global Annotation of Untargeted Metabolomics Data. Nat. Methods.

[24] Martins V., Szakiel A., Teixeira A., Abdallah C., Moreira C., Pączkowski C., Lanoue A., Gerós H. (2023). Combined Omics Approaches Expose Metabolite-Microbiota Correlations in Grape Berries of Three Cultivars of Douro Wine Region. Food Chem..

[25] Martins V., Abdallah C., Teixeira A., Moreira C., Nóbrega M., Lanoue A., Gerós H. (2025). Subregional Pedoclimatic Conditions with Contrasted UV-Radiation Shape Host-Microbiome and Metabolome Phenotypes in the Grape Berry. Food Chem. X.

[26] Nicolas S., Bois B., Billet K., Romanet R., Bahut F., Uhl J., Schmitt-Kopplin P., Gougeon R.D. (2024). High-Resolution Mass Spectrometry-Based Metabolomics for Increased Grape Juice Metabolite Coverage. Foods.

[27] Chu S., Wang S., Zhang R., Yin M., Yang X., Shi Q. (2022). Integrative Analysis of Transcriptomic and Metabolomic Profiles Reveals New Insights into the Molecular Foundation of Fruit Quality Formation in *Citrullus lanatus* (Thunb.) Matsum. & Nakai. Food Qual. Saf..

[28] Pang Z., Chong J., Zhou G., de Lima Morais D.A., Chang L., Barrette M., Gauthier C., Jacques P.-É., Li S., Xia J. (2021). MetaboAnalyst 5.0: Narrowing the Gap Between Raw Spectra and Functional Insights. Nucleic Acids Res..

[29] Lu S., Wang J., Zhuge Y., Zhang M., Liu C., Jia H., Fang J. (2021). Integrative Analyses of Metabolomes and Transcriptomes Provide Insights into Flavonoid Variation in Grape Berries. J. Agric. Food Chem..

[30] VanderWeide J., Falchi R., Calderan A., Peterlunger E., Vrhovsek U., Sivilotti P., Sabbatini P. (2022). Juxtaposition of the Source-to-Sink Ratio and Fruit Exposure to Solar Radiation on cv. Merlot (*Vitis vinifera* L.) Berry Phenolics in a Cool Versus Warm Growing Region. J. Agric. Food Chem..

[31] Wang H., Yao X., Liu M., Xu X., Wang Y., Kong J., Chen W., Xu Z., Kuang Y., Fan P. (2025). Climate, Soil, and Viticultural Factors Differentially Affect the Sub-Regional Variations in Biochemical Compositions of Grape Berries. Sci. Hortic..

[32] Ju Y., Wang W., Yue X., Xue W., Zhang Y., Fang Y. (2023). Integrated Metabolomic and Transcriptomic Analysis Reveals the Mechanism Underlying the Accumulation of Anthocyanins and Other Flavonoids in the Flesh and Skin of Teinturier Grapes. Plant Physiol. Biochem..

[33] Yu H., Li H.-Y., Zhou S.-H., Cheng G., Wei R.-F., Zhou Y.-M., Zhang Y., Xie T.-L., Zhang L. (2024). The Metabolomic Profiling of the Flavonoid Compounds in Red Wine Grapes and the Impact of Training Systems in the Southern Subtropical Region of China. Int. J. Mol. Sci..

[34] Zhang L., Wang Z., Zhang C., Zhou S., Yuan C. (2024). Metabolomics Analysis Based on UHPLC-QqQ-MS/MS to Discriminate Grapes and Wines from Different Geographical Origins and Climatological Characteristics. Food Chem. X.

[35] Zhang K., Zhang J., Zheng T., Gu W., Zhang Y., Li W., Zhou P., Fang Y., Chen K. (2024). Preharvest Application of MeJA Enhancing the Quality of Postharvest Grape Berries via Regulating Terpenes Biosynthesis and Phenylpropanoid Metabolisms. Food Chem..

[36] Liu M., Ji H., Jiang Q., Liu T., Cao H., Zhang Z. (2024). Effects of Full Shading of Clusters from Véraison to Ripeness on Fruit Quality and Volatile Compounds in Cabernet Sauvignon Grapes. Food Chem. X.

[37] Lu H.-C., Tian M.-B., Shi N., Li H.-Q., Li M.-Y., Cheng C.-F., Chen W., Li S.-D., He F., Duan C.-Q. (2024). Volatilomics of Cabernet Sauvignon Grapes and Sensory Perception of Wines Are Affected by Canopy Side in Vineyards with Different Row Orientations. Food Chem..

[38] Fu J., Liu C., Tu S., Liu H., Liu Z., He W., Dong L., Yi G., Xia Y., Wang J. (2025). Dietary Intervention with Resistant Starch-Rich Unripe Plantain Flour Restores Gut Microbiome–Metabolome Axis and Ameliorates Type 2 Diabetes in Rats. Foods.

