# Supplementary material for: Analysis of Metabolic Differences Between Marselan and Cabernet Sauvignon Grape Berries from the Manas Wine-Producing Region of Xinjiang Based on Untargeted Metabolomics
Source: Foods. 2026 Oct 3;15(19):3534. doi: 10.3390/foods15193534 (PMC13665544; doi:10.3390/foods15193534)
Supplement: Supplementary file 1 [file foods-15-03534-s001.zip › foods-4532769-supplementary.pdf]

# Supplemental Materials

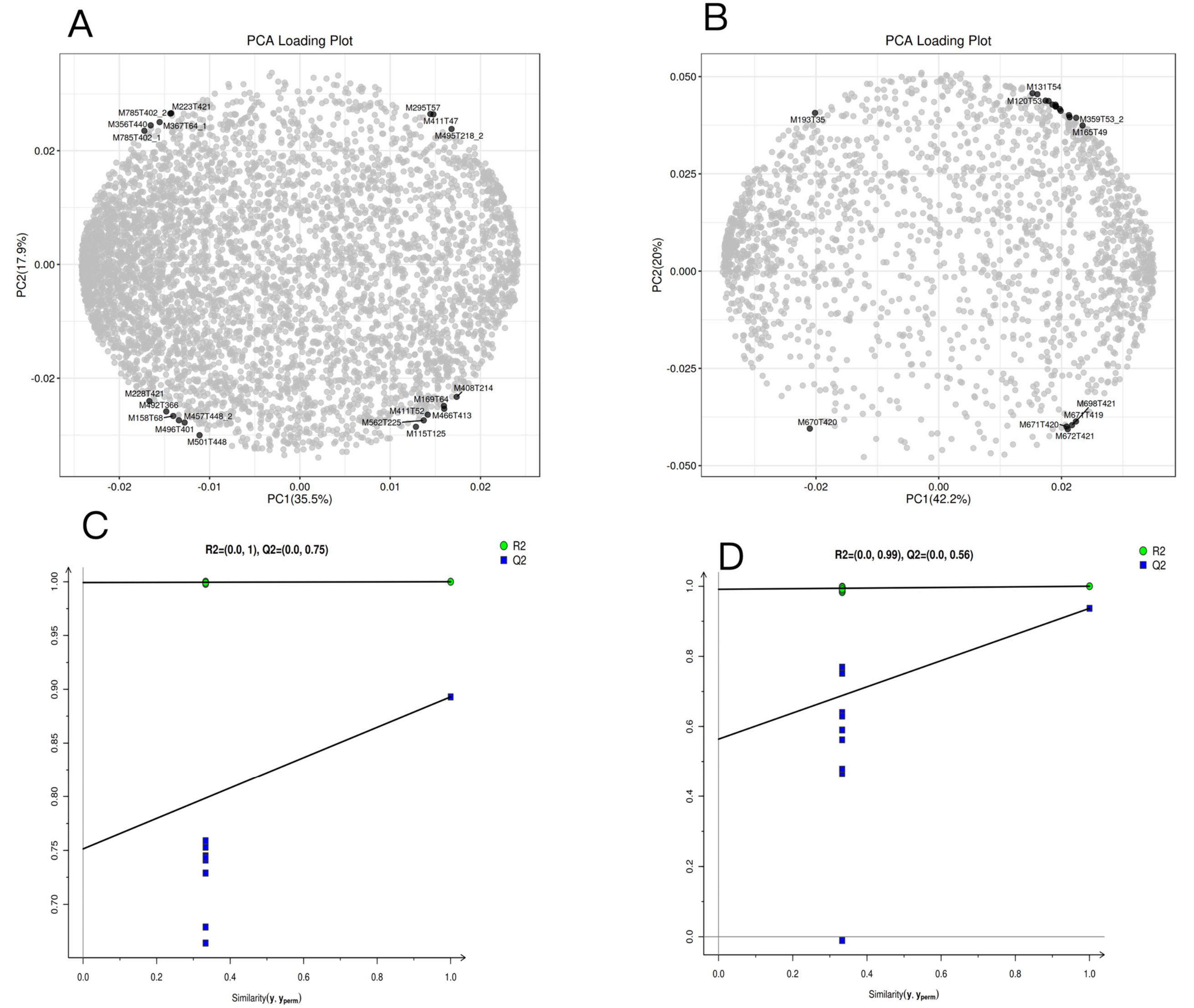


**Figure S1. PCA loading plots and PLS-DA permutation tests of Marselan and Cabernet Sauvignon grape berry metabolomic profiles in positive- and negative-ion modes. (A) PCA loading plot in positive-ion mode; (B) PCA loading plot in negative-ion mode; (C) permutation test of the PLS-DA model in positive-ion mode; (D) permutation test of the PLS-DA model in negative-ion mode.**

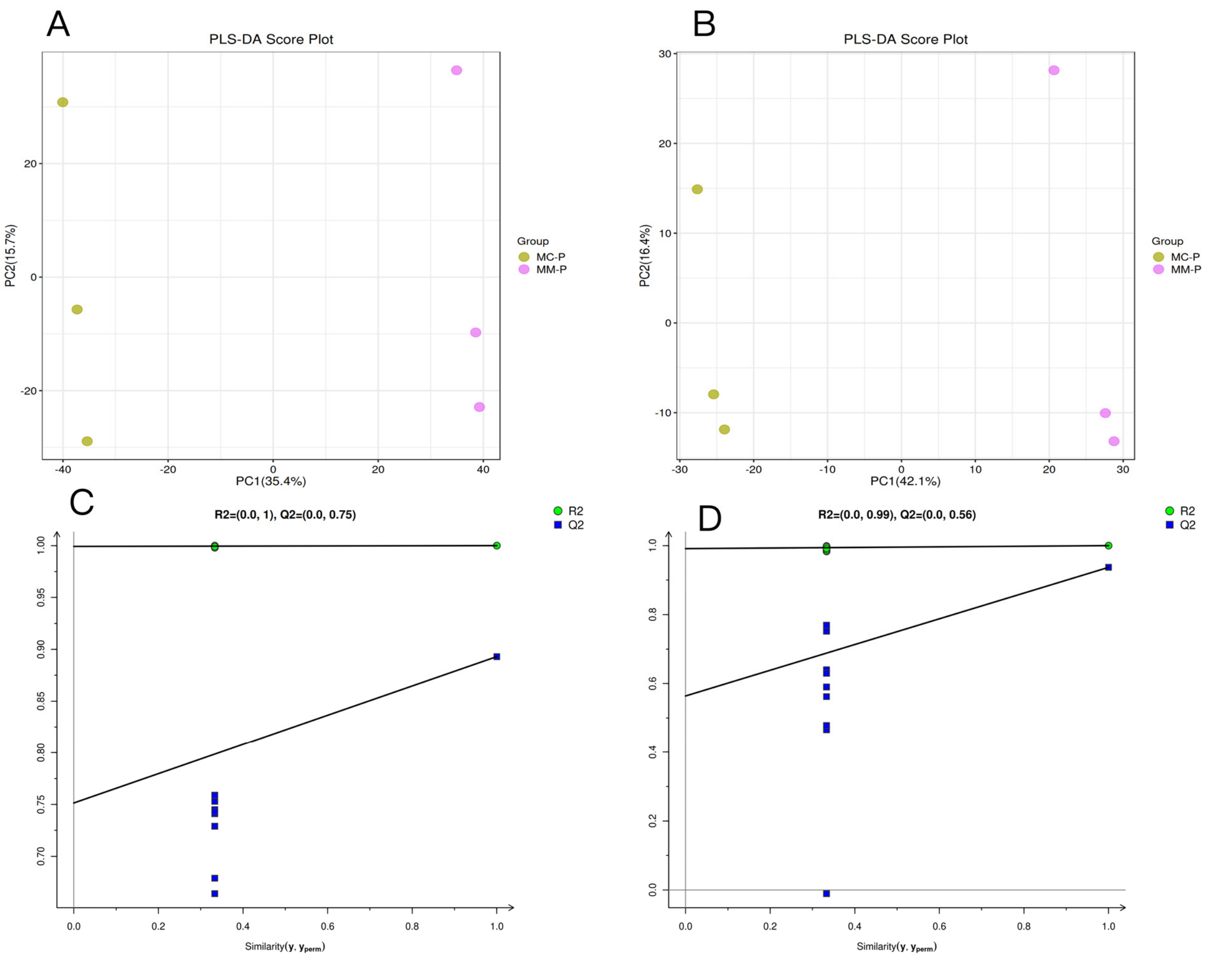


**Figure S2. PLS-DA score plots and permutation tests of Marselan and Cabernet Sauvignon grape berry metabolomic profiles in positive- and negative-ion modes. (A) PLS-DA score plot in positive-ion mode; (B) PLS-DA score plot in negative-ion mode; (C) permutation test of the PLS-DA model in positive-ion mode; (D) permutation test of the PLS-DA model in negative-ion mode.**

**Table S1. Summary of the current annotation-level labels for candidate and stricter statistical feature sets.**

| Feature set | Total | MSI_level1 | MSI_level2 | MSI_level3 |
|---|---|---|---|---|
| Candidate differential metabolites (VIP>1 and raw P<0.05) | 236 | 93 | 92 | 51 |
| Metabolites meeting stricter statistical criteria (FDR≤0.05 and $\lvert log_2FC \rvert$≥1) | 34 | 15 | 11 | 8 |

**Table S2A. Identification and annotation information for the 236 candidate differential metabolites (VIP>1 and raw P<0.05).**

| ID | Metabolite | m/z | RT (s) | ppm | Formula | Ion mode | Adduct | Library | Current annotation level |
|---|---|---|---|---|---|---|---|---|---|
| M103T84 | Styrene Oxide | 103.0549 | 84.10 | 6.567 | C8H8O | pos | [M-H2O+H]+ | BioDeepDB | MSI_level1 |
| M104T51_1 | 2-Aminoisobutyric acid | 104.0711 | 51.40 | 4.787 | C4H9NO2 | pos | [M+H]+ | BioDeepDB | MSI_level1 |
| M104T51_2 | 2-Pentanone | 104.1076 | 51.00 | 5.894 | C5H10O | pos | [M+NH4]+ | BioDeepDB | MSI_level1 |
| M111T328 | Furaneol (keto form) | 111.0205 | 327.90 | 5.903 | C6H7O3- | pos | [M-NH3+H]+ | BioDeepDB | MSI_level3 |
| M115T213 | Heptanal | 115.0392 | 213.00 | 2.008 | C7H14O | pos | [M+H]+ | mzVault | MSI_level2 |
| M116T54_2 | L-Proline | 116.0709 | 53.50 | 2.569 | C5H9NO2 | pos | [M+H]+ | BioDeepDB | MSI_level1 |
| M117T70 | α-Ketoisovaleric acid | 117.0552 | 70.20 | 4.970 | C5H8O3 | pos | [M+H]+ | BioDeepDB | MSI_level3 |
| M118T151 | 4-Methylaminobutyrate | 118.0865 | 150.60 | 2.108 | C5H11NO2 | pos | [M+H]+ | BioDeepDB | MSI_level1 |
| M120T84 | Indoline | 120.0812 | 84.10 | 3.566 | C8H9N | pos | [M+H]+ | BioDeepDB | MSI_level1 |
| M123T44 | Carbamic acid | 123.0408 | 44.20 | 6.242 | CH3NO2 | pos | [2M+H]+ | BioDeepDB | MSI_level3 |
| M128T64 | 3-Chlorocatechol | 127.9799 | 64.50 | 10.678 | C6H5ClO2 | pos | [M-NH3+H]+ | BioDeepDB | MSI_level3 |
| M129T122 | (±)-Furaneol | 129.0556 | 122.20 | 7.607 | C6H8O3 | pos | [M+H]+ | BioDeepDB | MSI_level1 |
| M130T202 | Triethylenetetramine | 130.0862 | 202.20 | 0.383 | C6H18N4 | pos | [M-NH3+H]+ | MoNA | MSI_level2 |
| M130T234 | Isoquinoline | 130.0656 | 233.90 | 3.670 | C9H7N | pos | [M+H]+ | BioDeepDB | MSI_level1 |
| M132T138 | Hydroxymethyl indol-3-yl ketone | 132.0812 | 138.00 | 3.242 | C10H9NO2 | pos | [M-CO2+H]+ | BioDeepDB | MSI_level3 |
| M139T315 | Phosphoglycolic acid | 139.1119 | 314.70 | 1.186 | C2H5O6P | pos | [M-H2O+H]+ | mzVault | MSI_level2 |
| M141T141 | cis-4-Carboxymethylenebut-2-en-4-olide | 141.0105 | 141.20 | 2.359 | C6H4O4 | pos | [M+H]+ | BioDeepDB | MSI_level1 |
| M141T224 | Hexamethylene tetramine | 141.0543 | 223.80 | 2.256 | C6H12N4 | pos | [M+H]+ | mzVault | MSI_level2 |
| M142T138_1 | N-Hydroxy-1-aminonaphthalene | 142.0656 | 137.80 | 3.366 | C10H9NO | pos | [M-H2O+H]+ | BioDeepDB | MSI_level1 |
| M142T75 | 1,4,5,6-Tetrahydro-6-oxonicotinic acid | 142.1227 | 75.10 | 0.461 | C6H7NO3 | pos | [M+H]+ | BioDeepDB | MSI_level1 |
| M143T232 | 2-(dihydroxymethyl)-5-formylfuran | 143.0828 | 232.50 | 8.384 | C6H6O4 | pos | [M+H]+ | BioDeepDB | MSI_level1 |
| M144T138 | Desethyl atrazine | 144.0812 | 138.10 | 8.916 | C6H10ClN5 | pos | [M-CO2+H]+ | BioDeepDB | MSI_level3 |
| M145T138 | Cyanurodiamide | 145.0844 | 138.40 | 8.041 | C3H5N5O | pos | [M+NH4]+ | BioDeepDB | MSI_level3 |
| M146T53_1 | 5-Hydroxylysine | 146.0820 | 53.00 | 5.717 | C6H14N2O3 | pos | [M-NH3+H]+ | HMDB | MSI_level2 |
| M147T252 | m-Coumaric acid | 147.0447 | 252.00 | 4.399 | C9H8O3 | pos | [M-H2O+H]+ | BioDeepDB | MSI_level1 |
| M151T44 | 4-Hydroxymandelic acid | 151.0360 | 43.90 | 19.652 | C8H8O4 | pos | [M-H2O+H]+ | BioDeepDB | MSI_level1 |
| M152T56 | Acetaminophen | 152.0569 | 55.90 | 1.418 | C8H9NO2 | pos | [M+H]+ | MoNA | MSI_level2 |
| M153T34 | Dihydroxyacetone phosphate | 152.9945 | 34.10 | 1.547 | C3H7O6P | pos | [M-H2O+H]+ | BioDeepDB | MSI_level1 |
| M154T143 | Metanephrine | 154.1231 | 143.40 | 3.021 | C10H15NO3 | pos | [M-CO2+H]+ | BioDeepDB | MSI_level3 |
| M157T34 | trans-2- | 156.9664 | 33.90 | 14.641 | C6H3ClO4 | pos | [M-H2O+H]+ | BioDeepDB | MSI_level1 |

| ID | Metabolite | m/z | RT (s) | ppm | Formula | Ion mode | Adduct | Library | Current annotation level |
|---|---|---|---|---|---|---|---|---|---|
| | Chlorodienelactone | | | | | | | | |
| M159T138 | N-hydroxyl-tryptamine | 159.0921 | 137.80 | 2.705 | C10H12N2O | pos | [M-H2O+H]+ | BioDeepDB | MSI_level3 |
| M160T138 | Indoleacetaldehyde | 160.0761 | 137.80 | 2.581 | C10H9NO | pos | [M+H]+ | BioDeepDB | MSI_level3 |
| M160T412 | 4,4'-Diaminodibutylamine | 160.0980 | 411.90 | 7.400 | C8H21N3 | pos | [M+H]+ | HMDB | MSI_level2 |
| M162T102 | Naphthalene epoxide | 162.0908 | 102.20 | 3.314 | C10H8O | pos | [M+NH4]+ | BioDeepDB | MSI_level1 |
| M162T53 | Carnitine | 162.1129 | 53.00 | 2.687 | C7H15NO3 | pos | [M+H]+ | BioDeepDB | MSI_level1 |
| M164T212 | Ureidoperacrylic acid | 164.0697 | 212.10 | 19.010 | C4H6N2O4 | pos | [M+NH4]+ | BioDeepDB | MSI_level3 |
| M165T58 | 2-Hydroxycinnamic acid | 165.0538 | 57.70 | 4.351 | C9H8O3 | pos | [M+H]+ | BioDeepDB | MSI_level1 |
| M166T315 | 1-Phenyl-1,2-propanedione | 166.0857 | 314.60 | 3.325 | C9H8O2 | pos | [M+NH4]+ | BioDeepDB | MSI_level3 |
| M168T326 | 3α,4,5,7α-Tetrahydro-5-hydroxy-1H-isoindole-1,3(2H)-dione | 168.0663 | 326.30 | 4.660 | C8H9NO3 | pos | [M+H]+ | HMDB | MSI_level2 |
| M169T228 | 2',4',6'-Trihydroxyacetophenone | 169.0512 | 228.10 | 9.860 | C8H8O4 | pos | [M+H]+ | BioDeepDB | MSI_level1 |
| M170T138 | 5-Methylthiopentanaldoxime | 170.0603 | 137.80 | 4.125 | C6H13NOS | pos | [M+Na]+ | BioDeepDB | MSI_level3 |
| M177T250 | (-)-trans-3,4-Dihydro-4,8-dihydroxy-3-methyl-1H-2-benzopyran-1-one | 177.0914 | 250.40 | 2.254 | C10H10O4 | pos | [M-H2O+H]+ | mzVault | MSI_level2 |
| M177T46 | 3-(3,4-Methylenedioxyphenyl)propenal | 177.0518 | 46.30 | 15.917 | C10H8O3 | pos | [M+H]+ | HMDB | MSI_level2 |
| M178T141 | Naphthalene-1,2-diol | 178.0863 | 141.30 | 0.268 | C10H8O2 | pos | [M+NH4]+ | BioDeepDB | MSI_level1 |
| M178T297 | 2,3-Dihydro-7-methyl-5-propanoyl-1H-pyrrolizine | 178.1231 | 296.90 | 2.614 | C11H15NO | pos | [M+H]+ | HMDB | MSI_level2 |
| M179T45 | 2-Oxo-4-phenylbutyric acid | 179.0676 | 44.70 | 14.896 | C10H10O3 | pos | [M+H]+ | BioDeepDB | MSI_level1 |
| M181T225 | Pterin | 181.0864 | 224.60 | 17.487 | C6H5N5O | pos | [M+NH4]+ | BioDeepDB | MSI_level1 |
| M181T254_1 | Aspirin | 181.0508 | 254.10 | 6.997 | C9H8O4 | pos | [M+H]+ | HMDB | MSI_level2 |
| M181T330_2 | 3-Heptyl acetate | 181.1229 | 329.60 | 16.600 | C9H18O2 | pos | [M+Na]+ | mzVault | MSI_level2 |
| M183T47 | 3-Methyluric acid | 183.0505 | 46.80 | 4.179 | C6H6N4O3 | pos | [M+H]+ | BioDeepDB | MSI_level3 |
| M185T34 | 2,4-Diaminobiphenyl | 184.9858 | 33.90 | 6.663 | C12H12N2 | pos | [M+H]+ | mzVault | MSI_level2 |
| M186T315 | 1,2-Dihydrophthalic acid | 186.0757 | 314.70 | 2.054 | C8H8O4 | pos | [M+NH4]+ | BioDeepDB | MSI_level1 |
| M188T233 | Indoleacrylic acid | 188.0712 | 233.30 | 3.181 | C11H9NO2 | pos | [M+H]+ | BioDeepDB | MSI_level1 |
| M189T47 | N6,N6,N6-trimethyl-L-lysine | 189.1603 | 47.40 | 2.927 | C9H20N2O2 | pos | [M+H]+ | Metlin | MSI_level2 |
| M190T409 | 1-Nitronaphthalene-7,8-oxide | 190.0505 | 409.10 | 3.327 | C10H7NO3 | pos | [M+H]+ | BioDeepDB | MSI_level1 |
| M190T68 | N-Acetylglutamate | 190.0714 | 68.40 | 2.125 | C7H11NO5 | pos | [M+H]+ | BioDeepDB | MSI_level1 |
| M190T72 | Guanine | 190.1444 | 71.80 | 14.512 | C5H5N5O | pos | [M+K]+ | BioDeepDB | MSI_level3 |
| M191T229 | β-Damascenone | 191.1435 | 229.30 | 2.437 | C13H18O | pos | [M+H]+ | BioDeepDB | MSI_level3 |
| M191T259 | Methyl 2-hydroxybenzoate | 191.1437 | 258.90 | 7.289 | C8H8O3 | pos | [M+K]+ | BioDeepDB | MSI_level3 |
| M191T348 | 4-Ethyl-2-methoxyphenol | 191.1800 | 348.30 | 7.724 | C9H12O2 | pos | [M+K]+ | mzVault | MSI_level2 |

| ID | Metabolite | m/z | RT (s) | ppm | Formula | Ion mode | Adduct | Library | Current annotation level |
|---|---|---|---|---|---|---|---|---|---|
| M192T47 | 3-(2-Methylthio)ethylmalate | 192.0555 | 46.90 | 6.339 | C7H12O5S | pos | [M-NH3+H]+ | BioDeepDB | MSI_level1 |
| M195T94 | (-)-1-(Methylthio)propyl 1-propenyl disulfide | 195.0295 | 94.10 | 18.134 | C7H14S3 | pos | [M+H]+ | HMDB | MSI_level2 |
| M197T35 | Deoxyribose 1-phosphate | 197.0207 | 34.80 | 1.269 | C5H11O7P | pos | [M-H2O+H]+ | BioDeepDB | MSI_level3 |
| M199T254_2 | N,N,N-Trimethyl-Histidine | 199.1316 | 254.30 | 0.392 | C9H16N3O2+ | pos | [M+H]+ | BioDeepDB | MSI_level1 |
| M205T285 | (3Z,6E)-α-Farnesene | 205.1228 | 284.80 | 2.437 | C15H24 | pos | [M+H]+ | mzVault | MSI_level2 |
| M205T409 | 5-Oxo-1,2-campholide | 205.0873 | 409.20 | 18.480 | C10H14O3 | pos | [M+Na]+ | BioDeepDB | MSI_level1 |
| M206T233 | Indole-3-lactic acid | 206.0817 | 232.70 | 2.591 | C11H11NO3 | pos | [M+H]+ | BioDeepDB | MSI_level1 |
| M211T265 | 3-Amino-9-ethylcarbazole | 211.0972 | 265.00 | 3.407 | C14H14N2 | pos | [M+H]+ | HMDB | MSI_level2 |
| M214T222 | N-Benzoyl-4-hydroxyanthranilate | 214.0867 | 221.90 | 2.097 | C14H11NO4 | pos | [M-CO2+H]+ | mzVault | MSI_level2 |
| M219T46 | Germacrone | 219.0618 | 45.60 | 15.440 | C15H22O | pos | [M+H]+ | mzVault | MSI_level2 |
| M222T203 | 2'-Deoxysepiapterin | 222.0766 | 203.10 | 12.840 | C9H11N5O2 | pos | [M+H]+ | mzVault | MSI_level2 |
| M224T115 | Scoparone | 224.0915 | 115.40 | 1.033 | C11H10O4 | pos | [M+NH4]+ | BioDeepDB | MSI_level3 |
| M224T130 | N-Acetylphinothricin | 224.0115 | 129.70 | 11.568 | C7H14NO5P | pos | [M+H]+ | mzVault | MSI_level2 |
| M231T222 | 6-Hydroxymelatonin | 231.1130 | 221.70 | 0.874 | C13H16N2O3 | pos | [M-H2O+H]+ | BioDeepDB | MSI_level1 |
| M232T75 | S-Acetyldihydrolipoamide | 232.0823 | 75.30 | 0.546 | C10H19NO2S2 | pos | [M-H2O+H]+ | mzVault | MSI_level2 |
| M235T61 | Polygodial | 234.9903 | 60.70 | 8.614 | C15H22O2 | pos | [M+H]+ | mzVault | MSI_level2 |
| M247T284 | SCHEMBL14765177 | 247.1333 | 284.10 | 18.038 | C10H18N2O5 | pos | [M+H]+ | BioDeepDB | MSI_level1 |
| M247T57_2 | Lacinilene C | 247.1296 | 57.00 | 13.209 | C15H18O3 | pos | [M+H]+ | BioDeepDB | MSI_level3 |
| M248T390 | Thiamine | 248.1061 | 389.80 | 11.739 | C12H17N4OS | pos | [M-H2O+H]+ | BioDeepDB | MSI_level3 |
| M248T53 | Isoniazid α-ketoglutaric acid | 248.1136 | 52.80 | 2.991 | C11H11N3O5 | pos | [M-H2O+H]+ | mzVault | MSI_level2 |
| M249T220 | 7-Hydroxy-6-(methoxyacetyl)-2,2-dimethyl-2H-1-benzopyran | 249.1126 | 220.20 | 1.887 | C14H16O4 | pos | [M+H]+ | HMDB | MSI_level2 |
| M249T248 | Enoximone | 249.1125 | 248.20 | 1.485 | C12H12N2O2S | pos | [M+H]+ | mzVault | MSI_level2 |
| M252T53 | 5'-Deoxyadenosine | 252.1083 | 53.40 | 3.218 | C10H13N5O3 | pos | [M+H]+ | BioDeepDB | MSI_level1 |
| M253T257 | (3xi,5Z)-1,5-Octadien-3-ol | 253.1440 | 257.10 | 2.258 | C8H14O | pos | [2M+H]+ | HMDB | MSI_level2 |
| M253T380 | 3-Methyl-5-pentyl-2-furanpentanoic acid | 253.1802 | 380.00 | 1.533 | C15H24O3 | pos | [M+H]+ | mzVault | MSI_level2 |
| M256T388 | 3-Dehydrosphinganine | 256.3003 | 388.20 | 1.714 | C18H37NO2 | pos | [M-CO2+H]+ | BioDeepDB | MSI_level3 |
| M260T54 | Propranolol | 260.1132 | 53.50 | 1.312 | C16H21NO2 | pos | [M+H]+ | HMDB | MSI_level2 |
| M264T47_2 | 2'-Deoxycytidine-5'-monophosphoric acid | 264.0766 | 47.40 | 8.404 | C9H14N3O7P | pos | [M-CO2+H]+ | BioDeepDB | MSI_level3 |
| M265T285 | Artemisinin | 265.1436 | 284.60 | 0.650 | C15H22O5 | pos | [M-H2O+H]+ | MoNA | MSI_level2 |
| M265T47_2 | 3-(6-methylthio)hexylmalate | 265.1121 | 46.80 | 6.354 | C11H20O5S | pos | [M+H]+ | BioDeepDB | MSI_level1 |
| M265T73 | Pentalenolactone D | 265.1439 | 73.40 | 1.779 | C15H20O4 | pos | [M+H]+ | BioDeepDB | MSI_level1 |
| M266T53 | N-acetylneuraminate | 266.1238 | 53.00 | 1.417 | C11H19NO9 | pos | [M-CO2+H]+ | BioDeepDB | MSI_level3 |

| ID | Metabolite | m/z | RT (s) | ppm | Formula | Ion mode | Adduct | Library | Current annotation level |
|---|---|---|---|---|---|---|---|---|---|
| M273T243 | Luteoforol | 273.0763 | 243.30 | 2.029 | C15H14O6 | pos | [M-H2O+H]+ | BioDeepDB | MSI_level1 |
| M274T329 | Palmitic acid | 274.2743 | 329.50 | 0.938 | C16H32O2 | pos | [M+NH4]+ | BioDeepDB | MSI_level1 |
| M275T268 | Ropivacaine | 275.2012 | 268.00 | 2.376 | C17H26N2O | pos | [M+H]+ | mzVault | MSI_level2 |
| M275T294 | 5α-Androstane-3α,17α-diol | 275.2006 | 293.80 | 0.199 | C19H32O2 | pos | [M-H2O+H]+ | mzVault | MSI_level2 |
| M275T382 | FA 18_4;O | 275.2006 | 382.10 | 0.199 | C18H28O3 | pos | [M-H2O+H]+ | BioDeepDB | MSI_level1 |
| M277T371 | FA(18_4) | 277.2172 | 371.20 | 3.625 | C18H28O2 | pos | [M+H]+ | BioDeepDB | MSI_level1 |
| M278T427 | Benznidazole | 278.1403 | 427.10 | 5.828 | C12H12N4O3 | pos | [M+NH4]+ | mzVault | MSI_level2 |
| M279T409 | Dibutyl phthalate | 279.1597 | 409.20 | 2.229 | C16H22O4 | pos | [M+H]+ | MoNA | MSI_level2 |
| M291T325 | FA 20_2 | 291.1954 | 324.90 | 0.207 | C20H36O2 | pos | [M-H2O+H]+ | HMDB | MSI_level2 |
| M291T85 | Leucopelargonidin | 291.0869 | 85.40 | 2.024 | C15H14O6 | pos | [M+H]+ | BioDeepDB | MSI_level3 |
| M293T294 | ACMC-20m5rp | 293.2113 | 293.80 | 0.650 | C18H30O4 | pos | [M-H2O+H]+ | BioDeepDB | MSI_level1 |
| M298T420 | Albendazole sulfone | 298.3108 | 420.50 | 1.258 | C12H15N3O4S | pos | [M+H]+ | HMDB | MSI_level2 |
| M298T60 | Pisatin | 298.0617 | 60.20 | 6.503 | C17H14O6 | pos | [M-NH3+H]+ | mzVault | MSI_level2 |
| M298T85 | 6-(α-D-glucosaminyl)-1D-myo-inositol | 298.1499 | 84.70 | 0.885 | C12H23NO10 | pos | [M-CO2+H]+ | BioDeepDB | MSI_level3 |
| M301T251 | Sophorol | 301.0713 | 251.10 | 1.788 | C16H12O6 | pos | [M+H]+ | BioDeepDB | MSI_level1 |
| M302T362 | Noroxycodone | 302.3057 | 361.70 | 1.522 | C17H19NO4 | pos | [M+H]+ | HMDB | MSI_level2 |
| M305T226 | (+)-Taxifolin | 305.0660 | 226.20 | 1.388 | C15H12O7 | pos | [M+H]+ | HMDB | MSI_level2 |
| M307T55_1 | (−)-Epigallocatechin | 307.0795 | 54.50 | 5.620 | C15H14O7 | pos | [M+H]+ | BioDeepDB | MSI_level3 |
| M307T70 | Leucocyanidin | 307.0802 | 70.50 | 3.341 | C15H14O7 | pos | [M+H]+ | BioDeepDB | MSI_level3 |
| M317T229 | 3,4-Dihydroxy-9,10-secoandrosta-1,3,5(10)-triene-9,17-dione | 317.0666 | 229.40 | 3.228 | C19H24O4 | pos | [M+H]+ | Metlin | MSI_level2 |
| M318T52 | N-acetylglucosaminylasparagine | 318.1305 | 52.30 | 2.923 | C12H21N3O8 | pos | [M-H2O+H]+ | BioDeepDB | MSI_level1 |
| M329T218 | Desulfosinalbin | 329.0658 | 218.20 | 9.560 | C14H19NO7S | pos | [M-NH3+H]+ | BioDeepDB | MSI_level3 |
| M329T251 | Aflatoxin M1 | 329.0657 | 251.20 | 0.375 | C17H12O7 | pos | [M+H]+ | BioDeepDB | MSI_level3 |
| M329T424 | Reticulinylium | 329.1604 | 424.00 | 5.321 | C19H22NO4+ | pos | [M+H]+ | BioDeepDB | MSI_level1 |
| M330T237 | Glutathione | 330.0737 | 237.10 | 2.051 | C10H17N3O6S | pos | [M+Na]+ | BioDeepDB | MSI_level3 |
| M330T57 | 3-O-β-D-galactopyranosyl-L-arabinose | 330.1396 | 57.50 | 0.402 | C11H20O10 | pos | [M+NH4]+ | HMDB | MSI_level2 |
| M331T218 | 3,7-Dimethylquercetin | 331.0812 | 218.20 | 2.338 | C17H14O7 | pos | [M+H]+ | BioDeepDB | MSI_level3 |
| M331T251_1 | (−)-Bisdechlorogeodin | 331.0820 | 251.00 | 2.338 | C17H14O7 | pos | [M+H]+ | BioDeepDB | MSI_level1 |
| M334T365 | Perilloside C | 334.2752 | 364.70 | 3.462 | C16H28O6 | pos | [M+NH4]+ | mzVault | MSI_level2 |
| M337T357 | 10S-HpOME | 337.2361 | 357.00 | 3.508 | C18H34O4 | pos | [M+Na]+ | BioDeepDB | MSI_level1 |
| M337T421 | 10,11-dihydro-12-oxo-LTB4 | 337.2742 | 420.80 | 1.462 | C20H32O4 | pos | [M+H]+ | mzVault | MSI_level2 |
| M344T192 | trans-p-Coumaric acid 4-glucoside | 344.1354 | 192.30 | 4.102 | C15H18O8 | pos | [M+NH4]+ | BioDeepDB | MSI_level1 |
| M349T181 | Inosine 5'-monophosphate (IMP) | 349.0584 | 180.80 | 11.530 | C10H13N4O8P | pos | [M+H]+ | BioDeepDB | MSI_level3 |
| M351T294 | 3′-O-Methyl-(-)-epicatechin-7-O-sulphate | 351.2149 | 294.00 | 4.808 | C16H16O8S | pos | [M-H2O+H]+ | mzVault | MSI_level2 |

| ID | Metabolite | m/z | RT (s) | ppm | Formula | Ion mode | Adduct | Library | Current annotation level |
|---|---|---|---|---|---|---|---|---|---|
| M367T112 | 4-O-β-D-mannopyranosyl-N-acetyl-D-glucosamine | 367.1505 | 112.40 | 8.263 | C14H25NO11 | pos | [M-NH3+H]+ | mzVault | MSI_level2 |
| M369T395 | Lathosterol | 369.3492 | 395.20 | 6.384 | C27H46O | pos | [M-H2O+H]+ | BioDeepDB | MSI_level1 |
| M369T423 | Cholesterol | 369.3499 | 423.10 | 4.760 | C27H46O | pos | [M-H2O+H]+ | BioDeepDB | MSI_level1 |
| M370T57 | Arbutin-6P | 370.0834 | 57.50 | 17.176 | C12H17O10P | pos | [M+NH4]+ | BioDeepDB | MSI_level1 |
| M397T416 | ST 29_1;O | 397.3838 | 415.60 | 2.374 | C29H50O | pos | [M-H2O+H]+ | BioDeepDB | MSI_level1 |
| M403T424_1 | Trandolaprilat | 403.2322 | 424.30 | 1.073 | C22H30N2O5 | pos | [M+H]+ | mzVault | MSI_level2 |
| M407T71 | Shanzhiside methyl ester | 407.1520 | 70.90 | 6.830 | C17H26O11 | pos | [M+H]+ | HMDB | MSI_level2 |
| M423T398 | 4α-Methyl-5α-cholest-7-en-3β-ol | 423.3640 | 397.90 | 10.118 | C28H48O | pos | [M+Na]+ | BioDeepDB | MSI_level1 |
| M433T228 | Dehydrochlortetracycline | 433.1139 | 227.90 | 5.043 | C22H21ClN2O8 | pos | [M-CO2+H]+ | BioDeepDB | MSI_level1 |
| M437T354_1 | Kanzonol K | 437.1925 | 354.00 | 7.674 | C26H28O6 | pos | [M+H]+ | HMDB | MSI_level2 |
| M441T254 | 8-Demethyltetracenomycin C | 441.0823 | 254.00 | 1.543 | C22H18O11 | pos | [M-H2O+H]+ | BioDeepDB | MSI_level1 |
| M455T348 | 13′-Carboxy-α-tocotrienol | 455.3504 | 347.60 | 3.411 | C29H42O4 | pos | [M+H]+ | mzVault | MSI_level2 |
| M455T405 | β-Citraurin | 455.2970 | 405.00 | 10.906 | C30H40O2 | pos | [M+Na]+ | BioDeepDB | MSI_level3 |
| M459T440 | Soyasapogenol B | 459.3845 | 440.30 | 2.718 | C30H50O3 | pos | [M+H]+ | BioDeepDB | MSI_level1 |
| M460T354_2 | Apstatin | 460.2809 | 354.00 | 0.687 | C23H33N5O5 | pos | [M+H]+ | mzVault | MSI_level2 |
| M465T88 | 3,5,7-Trihydroxy-2-[3-hydroxy-4-[(2S,5S)-3,4,5-trihydroxy-6-(hydroxymethyl)oxan-2-yl]oxyphenyl]chromen-4-one | 465.1049 | 87.70 | 4.626 | C21H20O12 | pos | [M+H]+ | HMDB | MSI_level2 |
| M473T348 | 6-Deoxocastasterone | 473.3618 | 347.60 | 3.569 | C28H50O4 | pos | [M+Na]+ | BioDeepDB | MSI_level3 |
| M479T427_1 | Iloprost | 479.2611 | 427.10 | 1.579 | C30H38O5 | pos | [M+H]+ | mzVault | MSI_level2 |
| M479T427_2 | 2-Methoxy-estradiol-17β 3-glucuronide | 479.2762 | 427.10 | 6.231 | C25H34O9 | pos | [M+H]+ | mzVault | MSI_level2 |
| M493T218_2 | Tricin 7-glucoside | 493.1347 | 218.20 | 1.325 | C23H24O12 | pos | [M+H]+ | HMDB | MSI_level2 |
| M507T223_1 | Quercetin 3-(6′-malonyl-glucoside) | 507.1136 | 222.80 | 0.567 | C24H22O15 | pos | [M-CO2+H]+ | BioDeepDB | MSI_level1 |
| M573T361 | Butirosin B | 573.3034 | 361.50 | 9.740 | C21H41N5O12 | pos | [M+NH4]+ | BioDeepDB | MSI_level1 |
| M577T254 | Aromadendrin | 577.1336 | 254.40 | 0.774 | C15H12O6 | pos | [2M+H]+ | BioDeepDB | MSI_level3 |
| M579T242_1 | Pelargonin | 579.1414 | 241.50 | 9.695 | C27H31O15+ | pos | [M-NH3+H]+ | BioDeepDB | MSI_level1 |
| M584T447 | 3-Hydroxyechinenone | 584.4381 | 446.80 | 13.832 | C40H54O2 | pos | [M+NH4]+ | BioDeepDB | MSI_level3 |
| M595T231_2 | Flavosativaside | 595.1509 | 231.00 | 19.893 | C27H30O15 | pos | [M+H]+ | BioDeepDB | MSI_level1 |
| M595T243_1 | Cyanidin 3-sophoroside | 595.1402 | 243.40 | 2.907 | C27H31O16+ | pos | [M-NH3+H]+ | BioDeepDB | MSI_level1 |
| M600T435_2 | ACMC-20dgc9 | 600.4728 | 435.20 | 7.797 | C35H38N2O6 | pos | [M+NH4]+ | BioDeepDB | MSI_level1 |
| M609T251_3 | Neodiosmin | 609.1720 | 251.10 | 15.412 | C28H32O15 | pos | [M+H]+ | HMDB | MSI_level2 |
| M611T239 | Delphinidin 3-sophoroside | 611.1414 | 238.80 | 7.453 | C27H31O17 | pos | [M-NH3+H]+ | BioDeepDB | MSI_level1 |
| M611T251_1 | Rutin | 611.1631 | 251.10 | 1.056 | C27H30O16 | pos | [M+H]+ | BioDeepDB | MSI_level1 |
| M625T268 | Isorhamnetin 3-neohesperoside | 625.1732 | 268.10 | 4.964 | C28H32O16 | pos | [M+H]+ | HMDB | MSI_level2 |

| ID | Metabolite | m/z | RT (s) | ppm | Formula | Ion mode | Adduct | Library | Current annotation level |
|---|---|---|---|---|---|---|---|---|---|
| M627T231 | 5'-Phosphoribosyl-N-formylglycinamidine | 627.1336 | 230.90 | 13.831 | C8H16N3O8P | pos | [2M+H]+ | BioDeepDB | MSI_level3 |
| M639T250 | Malvidin 3-laminaribioside | 639.1669 | 249.90 | 1.945 | C29H35O17 | pos | [M-NH3+H]+ | HMDB | MSI_level2 |
| M655T242_1 | Rhamnazin 3-sophoroside | 655.1538 | 241.90 | 5.061 | C29H34O17 | pos | [M+H]+ | HMDB | MSI_level2 |
| M655T242_2 | 3-methylthiopropyl-desulfoglucosinolate | 655.1687 | 241.90 | 0.955 | C11H21NO6S2 | pos | [2M+H]+ | BioDeepDB | MSI_level3 |
| M669T251_1 | 8-Demethyl-8-(2,3,4-O-trimethyl-α-L-rhamnosyl)tetracenomycin C | 669.1753 | 251.20 | 5.502 | C31H34O15 | pos | [M+Na]+ | BioDeepDB | MSI_level3 |
| M672T448 | PC(14_1(9Z)_15_0) | 672.4890 | 447.50 | 10.765 | C37H72NO8P | pos | [M-H2O+H]+ | HMDB | MSI_level2 |
| M673T208_2 | c-di-GMP | 673.0977 | 208.10 | 9.096 | C20H24N10O14P2 | pos | [M-H2O+H]+ | BioDeepDB | MSI_level1 |
| M679T251_2 | UDP-D-Man(2NAc3NAm)A | 679.1745 | 251.40 | 1.365 | C19H29N5O17P2 | pos | [M+NH4]+ | mzVault | MSI_level2 |
| M689T433_3 | PC(14_1(9Z)_P-16_0) | 688.5313 | 433.30 | 5.463 | C38H74NO7P | pos | [M+H]+ | HMDB | MSI_level2 |
| M731T250_2 | Selenodiglutathione | 731.1693 | 249.50 | 4.202 | C20H32N6O12S2Se | pos | [M+K]+ | mzVault | MSI_level2 |
| M733T431 | PC(18_3(6Z,9Z,12Z)_18_4(6Z,9Z,12Z,15Z)) | 732.5454 | 431.40 | 17.441 | C44H74NO8P | pos | [M-CO2+H]+ | HMDB | MSI_level2 |
| M791T448_1 | PC(16_0_22_5) | 790.5833 | 448.30 | 11.149 | C46H82NO8P | pos | [M-H2O+H]+ | HMDB | MSI_level2 |
| M125T128 | 4-hydroxy-5-methyl-2-methylene-3(2H)-furanone | 125.0234 | 127.90 | 8.128 | C6H6O3 | neg | [M-H]- | BioDeepDB | MSI_level1 |
| M143T56 | 4-Hydroxy-2-oxoglutaric acid | 142.9982 | 55.80 | 2.771 | C5H6O6 | neg | [M-H2O-H]- | BioDeepDB | MSI_level1 |
| M147T97 | 7-Methylxanthine | 147.0288 | 97.40 | 15.862 | C6H6N4O2 | neg | [M-H2O-H]- | BioDeepDB | MSI_level1 |
| M149T135 | Phenylglyoxylic acid | 149.0444 | 135.10 | 7.679 | C8H6O3 | neg | [M-H]- | mzVault | MSI_level2 |
| M149T209 | Tartronate semialdehyde | 149.0081 | 209.50 | 7.127 | C3H4O4 | neg | [M+HCOO]- | BioDeepDB | MSI_level3 |
| M149T246 | Homogentisic acid | 149.0445 | 246.40 | 15.977 | C8H8O4 | neg | [M-H2O-H]- | BioDeepDB | MSI_level1 |
| M153T320 | Acetol phosphate | 152.9949 | 319.80 | 6.103 | C3H7O5P | neg | [M-H]- | BioDeepDB | MSI_level1 |
| M154T55 | L-Histidine | 154.0620 | 55.50 | 1.279 | C6H9N3O2 | neg | [M-H]- | BioDeepDB | MSI_level1 |
| M154T80 | (3S,5S)-Carbapenam-3-carboxylate | 154.0610 | 80.40 | 7.769 | C7H9NO3 | neg | [M-H]- | BioDeepDB | MSI_level1 |
| M164T75 | L-Phenylalanine | 164.0715 | 74.80 | 1.208 | C9H11NO2 | neg | [M-H]- | BioDeepDB | MSI_level1 |
| M171T442 | FAL 8_1 | 171.1015 | 442.30 | 6.797 | C8H14O | neg | [M+HCOO]- | BioDeepDB | MSI_level1 |
| M173T139 | N-Methyltryptamine | 173.1038 | 139.10 | 3.431 | C11H14N2 | neg | [M-H]- | BioDeepDB | MSI_level1 |
| M204T97 | 5-Acetamidovalerate | 204.0854 | 96.70 | 11.477 | C7H13NO3 | neg | [M+HCOO]- | BioDeepDB | MSI_level1 |
| M225T132 | D-Psicose | 225.0566 | 132.40 | 3.931 | C6H12O6 | neg | [M+HCOO]- | BioDeepDB | MSI_level1 |
| M245T49 | Psoralen | 245.0426 | 48.80 | 12.018 | C11H6O3 | neg | [M+CH3CO2]- | BioDeepDB | MSI_level3 |
| M247T41 | 2,3-Diphosphoglyceric acid | 246.9387 | 41.30 | 11.061 | C3H8O10P2 | neg | [M-H2O-H]- | BioDeepDB | MSI_level1 |
| M255T54 | Hamamelose | 255.0713 | 54.20 | 3.343 | C6H12O7 | neg | [M+CH3CO2]- | BioDeepDB | MSI_level1 |
| M261T53 | Helenalin | 261.1340 | 52.90 | 8.921 | C15H18O4 | neg | [M-H]- | mzVault | MSI_level2 |
| M275T42 | 6-Phosphogluconic acid | 275.0201 | 42.00 | 9.976 | C6H13O10P | neg | [M-H]- | BioDeepDB | MSI_level1 |
| M279T310 | (2S)-2-(2,4-dichlorophenoxy)propano | 278.9808 | 309.50 | 8.787 | C9H8Cl2O3 | neg | [M+HCOO]- | BioDeepDB | MSI_level1 |

| ID | Metabolite | m/z | RT (s) | ppm | Formula | Ion mode | Adduct | Library | Current annotation level |
|---|---|---|---|---|---|---|---|---|---|
| | ic acid | | | | | | | | |
| M287T223 | (+)-Gallocatechin | 287.0555 | 223.30 | 2.118 | C15H14O7 | neg | [M-H2O-H]- | BioDeepDB | MSI_level1 |
| M299T229 | Kaempferide | 299.0554 | 229.40 | 2.370 | C16H12O6 | neg | [M-H]- | BioDeepDB | MSI_level1 |
| M299T85 | Methylnissolin | 299.0764 | 85.20 | 0.649 | C17H16O5 | neg | [M-H]- | mzVault | MSI_level2 |
| M301T264 | Quercetin | 301.0347 | 263.80 | 2.241 | C15H10O7 | neg | [M-H]- | BioDeepDB | MSI_level1 |
| M309T54 | Flindersiachromone | 309.0826 | 53.60 | 17.718 | C17H14O2 | neg | [M+CH3CO2]- | mzVault | MSI_level2 |
| M321T50 | Alditol 6-phosphate | 321.0616 | 49.90 | 7.415 | C6H15O9P | neg | [M+CH3CO2]- | BioDeepDB | MSI_level3 |
| M325T198 | Anordiol | 325.0917 | 197.50 | 3.649 | C22H30O2 | neg | [M-H]- | mzVault | MSI_level2 |
| M329T193 | Tricin | 329.0662 | 192.90 | 1.741 | C17H14O7 | neg | [M-H]- | MoNA | MSI_level2 |
| M333T41 | 5,5-Dehydrodivanillate | 333.0246 | 40.90 | 1.815 | C16H14O8 | neg | [M-H]- | mzVault | MSI_level2 |
| M333T47 | 2-(β-D-Glucosyl)-sn-glycerol 3-phosphate | 333.0575 | 47.50 | 5.161 | C9H19O11P | neg | [M-H]- | BioDeepDB | MSI_level1 |
| M339T439 | Behenic acid | 339.3258 | 439.30 | 3.053 | C22H44O2 | neg | [M-H]- | BioDeepDB | MSI_level1 |
| M344T76 | ST 21_3;O5 | 344.1342 | 76.10 | 14.251 | C21H30O5 | neg | [M-NH3-H]- | mzVault | MSI_level2 |
| M345T426 | FA 18_0;O | 345.2640 | 426.30 | 1.837 | C18H36O3 | neg | [M+HCOO]- | BioDeepDB | MSI_level1 |
| M355T80 | 5-Amino-6-(5'-phosphoribitylamino)uracil | 355.0649 | 80.20 | 3.199 | C9H17N4O9P | neg | [M-H]- | BioDeepDB | MSI_level3 |
| M366T76 | Dehydrohistidyltryptophyldiketopiperazine | 366.1172 | 76.40 | 9.758 | C17H15N5O2 | neg | [M+HCOO]- | BioDeepDB | MSI_level3 |
| M378T103 | 12-Hydroxychelirubine | 378.0954 | 103.10 | 7.685 | C21H17NO6 | neg | [M-H]- | BioDeepDB | MSI_level1 |
| M407T197 | Chelirubine | 407.0982 | 196.60 | 6.991 | C21H16NO5+ | neg | [M+HCOO]- | BioDeepDB | MSI_level3 |
| M409T45 | Xanthylic acid | 409.0395 | 45.30 | 1.754 | C10H13N4O9P | neg | [M+HCOO]- | BioDeepDB | MSI_level3 |
| M429T69 | Wuweizisu C | 429.1586 | 69.40 | 7.261 | C22H24O6 | neg | [M+HCOO]- | mzVault | MSI_level2 |
| M433T417 | ST 27_1;O4 | 433.2347 | 416.90 | 3.118 | C27H46O4 | neg | [M-H]- | mzVault | MSI_level2 |
| M436T332 | 13-Desoxyterpendole I | 436.2447 | 331.70 | 10.587 | C27H35NO4 | neg | [M-H]- | mzVault | MSI_level2 |
| M449T106 | PHC 4-O-glucoside | 449.1066 | 105.70 | 5.188 | C21H22O11 | neg | [M-H]- | BioDeepDB | MSI_level3 |
| M449T220 | 2-Hydroxy-3,6,7,4-tetramethylquercetagetin | 449.1066 | 219.80 | 5.188 | C19H18O9 | neg | [M+CH3CO2]- | BioDeepDB | MSI_level3 |
| M449T297 | Clinofibrate | 449.2307 | 297.40 | 12.043 | C28H36O6 | neg | [M-H2O-H]- | mzVault | MSI_level2 |
| M455T396 | Betulinic acid | 455.3446 | 396.20 | 18.556 | C30H48O3 | neg | [M-H]- | mzVault | MSI_level2 |
| M463T425 | Bracteatin 6-glucoside | 463.2883 | 425.00 | 6.330 | C21H20O12 | neg | [M-H]- | mzVault | MSI_level2 |
| M479T228 | THC 4-glucoside | 479.1175 | 227.80 | 4.163 | C21H22O10 | neg | [M+HCOO]- | BioDeepDB | MSI_level3 |
| M491T208 | Sissotrin | 491.1184 | 207.50 | 2.229 | C22H22O10 | neg | [M+HCOO]- | BioDeepDB | MSI_level1 |
| M521T52 | Cleistanthin | 521.1689 | 51.50 | 4.719 | C28H28O11 | neg | [M-H2O-H]- | mzVault | MSI_level2 |
| M563T417 | 5-O-beta-D-mycaminosyl-20-oxotylonolide | 563.3325 | 416.50 | 17.684 | C31H51NO9 | neg | [M-NH3-H]- | mzVault | MSI_level2 |
| M567T235 | Trilobolide | 567.2053 | 235.30 | 5.298 | C27H38O10 | neg | [M+HCOO]- | mzVault | MSI_level2 |
| M637T245_2 | Malvin | 637.1649 | 245.00 | 17.750 | [C29H35O17]+ | neg | [M-NH3-H]- | MoNA | MSI_level2 |
| M652T250 | Spinacetin 3-gentiobioside | 652.1413 | 250.20 | 0.949 | C29H34O18 | neg | [M-NH3-H]- | mzVault | MSI_level2 |
| M683T52_2 | Sucrose | 683.2317 | 51.50 | 9.607 | C12H22O11 | neg | [2M-H]- | BioDeepDB | MSI_level1 |
| M702T418_2 | Erythromycin D | 702.4488 | 418.20 | 7.721 | C36H65NO12 | neg | [M-H]- | BioDeepDB | MSI_level1 |

| ID | Metabolite | m/z | RT (s) | ppm | Formula | Ion mode | Adduct | Library | Current annotation level |
|---|---|---|---|---|---|---|---|---|---|
| M703T421_2 | 20-Hydroxy-leukotriene B4 | 703.4477 | 420.80 | 7.182 | C20H32O5 | neg | [2M-H]- | BioDeepDB | MSI_level1 |
| M707T429 | Uvaricin | 707.4259 | 428.90 | 16.487 | C39H68O7 | neg | [M+CH3CO2]- | mzVault | MSI_level2 |
| M725T416 | (3S,2S)-4-Ketomyxol 2-α-L-fucoside | 725.4468 | 416.50 | 6.268 | C46H64O8 | neg | [M-H2O-H]- | BioDeepDB | MSI_level1 |
| M743T426 | 8,8a-Deoxyoleandolide | 743.4912 | 426.10 | 5.212 | C20H36O6 | neg | [2M-H]- | BioDeepDB | MSI_level3 |
| M765T433 | Ginsenoside | 765.4866 | 433.50 | 9.373 | C42H72O13 | neg | [M-H2O-H]- | mzVault | MSI_level2 |
| M789T424_2 | (2R,5E)-6,10-dimethyl-2-[(2S)-4-methyl-5-oxido-1,2-dihydrofuro[2,3-c]quinolin-5-ium-2-yl]undeca-5,9-dien-2-ol | 789.4914 | 423.60 | 8.379 | C25H33NO3 | neg | [2M-H]- | mzVault | MSI_level2 |
| M791T434 | Cholesterol cholate | 791.4938 | 434.20 | 1.611 | C51H84O6 | neg | [M-H]- | mzVault | MSI_level2 |
| M805T407 | Tulathromycin | 804.5689 | 407.30 | 12.219 | C41H79N3O12 | neg | [M-H]- | mzVault | MSI_level2 |
| M837T410_2 | Dehydroxypaxilline | 837.4923 | 409.70 | 8.973 | C27H33NO3 | neg | [2M-H]- | mzVault | MSI_level2 |
| M850T423_2 | Solanesyl-PP | 849.5222 | 422.90 | 2.056 | C45H76O7P2 | neg | [M+CH3CO2]- | BioDeepDB | MSI_level1 |
| M865T225_2 | Procyanidin C1 | 865.1851 | 225.40 | 15.521 | C45H38O18 | neg | [M-H]- | MoNA | MSI_level2 |

Note: RT, retention time; ppm, mass error.

## Table S2B. Relative abundances and statistical results for the 236 candidate differential metabolites.

| ID | Metabolite | MC-P1 | MC-P2 | MC-P3 | MM-P1 | MM-P2 | MM-P3 | FC | log₂FC | P value | FDR | VIP |
|---|---|---|---|---|---|---|---|---|---|---|---|---|
| M103T84 | Styrene Oxide | 5.220e+06 | 1.027e+07 | 9.762e+06 | 2.432e+07 | 2.661e+07 | 3.165e+07 | 0.31 | -1.71 | 0.0061 | 0.0955 | 1.575 |
| M104T51_1 | 2-Aminoisobutyric acid | 1.328e+08 | 2.039e+08 | 2.474e+08 | 1.059e+08 | 1.105e+08 | 1.198e+08 | 1.74 | 0.80 | 0.0497 | 0.2591 | 1.366 |
| M104T51_2 | 2-Pentanone | 9.813e+08 | 1.195e+09 | 1.290e+09 | 6.749e+08 | 7.142e+08 | 7.013e+08 | 1.66 | 0.73 | 0.0039 | 0.0798 | 1.597 |
| M111T328 | Furaneol (keto form) | 1.385e+09 | 5.514e+09 | 6.233e+09 | 9.630e+07 | 6.765e+08 | 6.765e+08 | 9.06 | 3.18 | 0.0452 | 0.2492 | 1.382 |
| M115T213 | Heptanal | 4.835e+07 | 7.309e+07 | 3.597e+07 | 2.919e+07 | 1.106e+07 | 1.332e+07 | 2.94 | 1.55 | 0.0355 | 0.2209 | 1.417 |
| M116T54_2 | L-Proline | 3.246e+10 | 3.364e+10 | 2.452e+10 | 1.356e+10 | 1.122e+10 | 1.338e+10 | 2.38 | 1.25 | 0.0018 | 0.0555 | 1.624 |
| M117T70 | α-Ketoisovaleric acid | 5.107e+07 | 5.616e+07 | 3.730e+07 | 1.059e+07 | 1.034e+07 | 2.138e+07 | 3.42 | 1.77 | 0.0090 | 0.1153 | 1.552 |
| M118T151 | 4-Methylaminobutyrate | 1.853e+07 | 1.822e+07 | 2.410e+07 | 2.977e+07 | 5.012e+07 | 3.270e+07 | 0.54 | -0.89 | 0.0316 | 0.2089 | 1.434 |
| M120T84 | Indoline | 4.716e+08 | 8.887e+08 | 8.656e+08 | 1.303e+09 | 1.230e+09 | 1.516e+09 | 0.55 | -0.86 | 0.0426 | 0.2440 | 1.391 |
| M123T44 | Carbamic acid | 5.288e+08 | 4.716e+08 | 6.102e+08 | 6.801e+08 | 6.574e+08 | 7.846e+08 | 0.76 | -0.40 | 0.0392 | 0.2342 | 1.402 |
| M128T64 | 3-Chlorocatechol | 5.998e+07 | 6.228e+07 | 6.990e+07 | 5.684e+07 | 5.240e+07 | 5.192e+07 | 1.19 | 0.25 | 0.0324 | 0.2113 | 1.429 |
| M129T122 | (±)-Furaneol | 1.687e+08 | 1.157e+08 | 5.894e+07 | 1.096e+07 | 1.110e+07 | 2.876e+07 | 6.76 | 2.76 | 0.0121 | 0.1382 | 1.531 |
| M130T202 | Triethylenetetramine | 2.191e+07 | 1.499e+07 | 1.261e+07 | 3.833e+06 | 3.453e+06 | 3.586e+06 | 4.55 | 2.19 | 8.510e-04 | 0.0417 | 1.643 |
| M130T234 | Isoquinoline | 1.658e+07 | 3.284e+07 | 4.535e+07 | 2.788e+06 | 2.972e+06 | 2.645e+06 | 11.28 | 3.50 | 0.0147 | 0.1512 | 1.631 |
| M132T138 | Hydroxymethyl indol-3-yl ketone | 1.748e+07 | 1.585e+07 | 2.827e+07 | 4.149e+07 | 5.934e+07 | 4.891e+07 | 0.41 | -1.28 | 0.0116 | 0.1364 | 1.533 |
| M139T315 | Phosphoglycolic acid | 2.290e+07 | 1.795e+07 | 2.983e+07 | 1.123e+07 | 1.357e+07 | 1.513e+07 | 1.77 | 0.82 | 0.0308 | 0.2069 | 1.437 |
| M141T141 | cis-4-Carboxymethylenebut-2-en-4-olide | 7.948e+06 | 8.136e+06 | 1.016e+07 | 1.218e+07 | 1.079e+07 | 1.226e+07 | 0.75 | -0.42 | 0.0280 | 0.1961 | 1.447 |
| M141T224 | Hexamethylene tetramine | 6.406e+06 | 1.460e+07 | 1.789e+07 | 3.526e+07 | 3.676e+07 | 5.870e+07 | 0.30 | -1.75 | 0.0229 | 0.1810 | 1.471 |
| M142T138_1 | N-Hydroxy-1-aminonaphthalene | 5.212e+06 | 5.747e+06 | 7.680e+06 | 1.181e+07 | 2.061e+07 | 1.744e+07 | 0.37 | -1.42 | 0.0086 | 0.1144 | 1.555 |
| M142T75 | 1,4,5,6-Tetrahydro-6-oxonicotinic acid | 7.048e+07 | 1.674e+08 | 1.457e+08 | 3.462e+07 | 3.119e+07 | 3.551e+07 | 3.79 | 1.92 | 0.0394 | 0.2352 | 1.547 |
| M143T232 | 2-(dihydroxymethyl)-5-formylfuran | 1.484e+07 | 1.359e+07 | 1.635e+07 | 1.241e+06 | 4.007e+06 | 1.235e+06 | 6.91 | 2.79 | 0.0312 | 0.2071 | 1.574 |
| M144T138 | Desethyl atrazine | 6.332e+07 | 5.549e+07 | 6.831e+07 | 1.438e+08 | 1.998e+08 | 1.648e+08 | 0.37 | -1.44 | 9.265e-04 | 0.0428 | 1.641 |
| M145T138 | Cyanurodiamide | 3.017e+06 | 5.647e+06 | 8.681e+06 | 1.327e+07 | 2.090e+07 | 1.443e+07 | 0.36 | -1.49 | 0.0310 | 0.2069 | 1.436 |

| ID | Metabolite | MC-P1 | MC-P2 | MC-P3 | MM-P1 | MM-P2 | MM-P3 | FC | $log_2$FC | P value | FDR | VIP |
|---|---|---|---|---|---|---|---|---|---|---|---|---|
| M146T53_1 | 5-Hydroxylysine | 4.767e+08 | 4.171e+08 | 3.792e+08 | 2.578e+08 | 2.734e+08 | 3.038e+08 | 1.52 | 0.61 | 0.0069 | 0.1037 | 1.569 |
| M147T252 | m-Coumaric acid | 1.287e+07 | 1.175e+07 | 6.348e+06 | 3.419e+07 | 3.028e+07 | 4.766e+07 | 0.28 | -1.86 | 0.0072 | 0.1046 | 1.564 |
| M151T44 | 4-Hydroxymandelic acid | 3.743e+08 | 3.208e+08 | 4.013e+08 | 2.598e+08 | 2.590e+08 | 2.997e+08 | 1.34 | 0.42 | 0.0238 | 0.1842 | 1.468 |
| M152T56 | Acetaminophen | 7.924e+07 | 8.562e+07 | 1.008e+08 | 6.848e+07 | 6.300e+07 | 6.338e+07 | 1.36 | 0.45 | 0.0157 | 0.1555 | 1.508 |
| M153T34 | Dihydroxyacetone phosphate | 1.149e+08 | 1.274e+08 | 1.105e+08 | 1.591e+08 | 1.760e+08 | 1.355e+08 | 0.75 | -0.42 | 0.0311 | 0.2069 | 1.437 |
| M154T143 | Metanephrine | 6.185e+06 | 7.406e+06 | 1.347e+07 | 2.173e+07 | 2.951e+07 | 1.483e+07 | 0.41 | -1.29 | 0.0417 | 0.2425 | 1.396 |
| M157T34 | trans-2-Chlorodienelactone | 5.968e+06 | 6.738e+06 | 5.842e+06 | 5.287e+06 | 4.418e+06 | 4.138e+06 | 1.34 | 0.42 | 0.0260 | 0.1904 | 1.456 |
| M159T138 | N-hydroxyl-tryptamine | 2.375e+07 | 2.897e+07 | 3.528e+07 | 6.592e+07 | 8.602e+07 | 7.520e+07 | 0.39 | -1.37 | 0.0023 | 0.0612 | 1.618 |
| M160T138 | Indoleacetaldehyde | 9.597e+06 | 1.176e+07 | 9.799e+06 | 2.640e+07 | 3.258e+07 | 2.049e+07 | 0.39 | -1.35 | 0.0034 | 0.0755 | 1.603 |
| M160T412 | 4,4'-Diaminodibutylamine | 1.930e+07 | 2.303e+07 | 1.175e+07 | 1.824e+06 | 4.730e+06 | 1.611e+06 | 6.62 | 2.73 | 0.0075 | 0.1064 | 1.562 |
| M162T102 | Naphthalene epoxide | 2.658e+07 | 4.570e+07 | 5.077e+07 | 1.836e+08 | 8.122e+07 | 1.300e+08 | 0.31 | -1.68 | 0.0207 | 0.1723 | 1.481 |
| M162T53 | Carnitine | 7.407e+07 | 1.256e+08 | 9.162e+07 | 2.922e+07 | 4.383e+07 | 5.404e+07 | 2.29 | 1.20 | 0.0242 | 0.1850 | 1.465 |
| M164T212 | Ureidoperacrylic acid | 9.258e+06 | 3.943e+06 | 4.503e+06 | 2.231e+07 | 1.470e+07 | 1.348e+07 | 0.35 | -1.51 | 0.0234 | 0.1827 | 1.468 |
| M165T58 | 2-Hydroxycinnamic acid | 2.119e+07 | 2.151e+07 | 2.210e+07 | 5.385e+06 | 5.563e+06 | 5.257e+06 | 4.00 | 2.00 | 2.849e-07 | 0.0014 | 1.682 |
| M166T315 | 1-Phenyl-1,2-propanedione | 8.563e+06 | 1.153e+07 | 1.603e+07 | 3.023e+06 | 6.488e+06 | 2.823e+06 | 2.93 | 1.55 | 0.0257 | 0.1904 | 1.457 |
| M168T326 | 3α,4,5,7α-Tetrahydro-5-hydroxy-1H-isoindole-1,3(2H)-dione | 1.180e+07 | 1.203e+07 | 1.467e+07 | 6.892e+07 | 1.925e+07 | 4.140e+07 | 0.30 | -1.75 | 0.0445 | 0.2480 | 1.383 |
| M169T228 | 2',4',6'-Trihydroxyacetophenone | 6.654e+07 | 7.306e+07 | 7.409e+07 | 1.273e+08 | 9.265e+07 | 9.813e+07 | 0.67 | -0.57 | 0.0198 | 0.1688 | 1.486 |
| M170T138 | 5-Methylthiopentanaldoxime | 2.161e+07 | 2.390e+07 | 3.199e+07 | 5.838e+07 | 8.202e+07 | 6.132e+07 | 0.38 | -1.38 | 0.0038 | 0.0792 | 1.599 |
| M177T250 | (-)-trans-3,4-Dihydro-4,8-dihydroxy-3-methyl-1H-2-benzopyran-1-one | 1.186e+07 | 1.216e+07 | 1.151e+07 | 1.874e+07 | 1.571e+07 | 1.388e+07 | 0.74 | -0.44 | 0.0276 | 0.1943 | 1.450 |
| M177T46 | 3-(3,4-Methylenedioxyphenyl)propenal | 4.024e+07 | 3.425e+07 | 3.947e+07 | 6.249e+07 | 6.093e+07 | 6.854e+07 | 0.59 | -0.75 | 0.0011 | 0.0459 | 1.637 |
| M178T141 | Naphthalene-1,2-diol | 3.168e+06 | 1.295e+07 | 1.298e+07 | 3.571e+07 | 5.572e+07 | 2.338e+07 | 0.25 | -1.98 | 0.0489 | 0.2578 | 1.371 |
| M178T297 | 2,3-Dihydro-7-methyl-5-propanoyl-1H-pyrrolizine | 2.620e+07 | 2.891e+07 | 4.069e+07 | 9.094e+06 | 1.102e+07 | 1.494e+07 | 2.73 | 1.45 | 0.0068 | 0.1037 | 1.569 |
| M179T45 | 2-Oxo-4-phenylbutyric acid | 1.669e+06 | 1.572e+06 | 2.285e+06 | 4.217e+06 | 6.282e+06 | 4.482e+06 | 0.37 | -1.44 | 0.0042 | 0.0817 | 1.594 |
| M181T225 | Pterin | 4.818e+06 | 4.565e+06 | 4.241e+06 | 5.996e+06 | 7.378e+06 | 6.684e+06 | 0.68 | -0.56 | 0.0054 | 0.0918 | 1.581 |
| M181T254_1 | Aspirin | 4.640e+07 | 5.772e+07 | 4.568e+07 | 3.642e+07 | 3.492e+07 | 3.763e+07 | 1.37 | 0.46 | 0.0164 | 0.1590 | 1.504 |
| M181T330_2 | 3-Heptyl acetate | 1.870e+07 | 2.049e+07 | 2.539e+07 | 1.606e+07 | 1.175e+07 | 1.152e+07 | 1.64 | 0.72 | 0.0238 | 0.1842 | 1.467 |
| M183T47 | 3-Methyluric acid | 1.419e+08 | 1.271e+08 | 1.191e+08 | 1.012e+08 | 1.013e+08 | 1.000e+08 | 1.28 | 0.36 | 0.0397 | 0.2361 | 1.554 |
| M185T34 | 2,4-Diaminobiphenyl | 1.644e+07 | 9.262e+06 | 2.052e+07 | 5.342e+06 | 6.221e+06 | 7.122e+06 | 2.47 | 1.31 | 0.0267 | 0.1909 | 1.455 |
| M186T315 | 1,2-Dihydrophthalic acid | 5.028e+06 | 9.230e+06 | 1.624e+07 | 2.008e+07 | 5.250e+07 | 4.958e+07 | 0.25 | -2.00 | 0.0372 | 0.2285 | 1.411 |
| M188T233 | Indoleacrylic acid | 4.327e+07 | 8.456e+07 | 1.122e+08 | 2.225e+06 | 1.033e+07 | 2.126e+06 | 16.35 | 4.03 | 0.0070 | 0.1039 | 1.566 |
| M189T47 | N6,N6,N6-trimethyl-L-lysine | 4.147e+06 | 1.623e+06 | 7.011e+06 | 4.555e+05 | 4.529e+05 | 4.679e+05 | 9.29 | 3.22 | 0.0403 | 0.2386 | 1.555 |
| M190T409 | 1-Nitronaphthalene-7,8-oxide | 3.169e+07 | 3.062e+07 | 3.873e+07 | 5.537e+07 | 5.161e+07 | 5.922e+07 | 0.61 | -0.72 | 0.0038 | 0.0798 | 1.597 |
| M190T68 | N-Acetylglutamate | 1.052e+07 | 9.193e+06 | 1.142e+07 | 3.207e+06 | 4.121e+06 | 2.059e+06 | 3.32 | 1.73 | 0.0044 | 0.0830 | 1.591 |
| M190T72 | Guanine | 1.049e+07 | 1.240e+07 | 9.282e+06 | 6.585e+06 | 6.152e+06 | 5.759e+06 | 1.74 | 0.80 | 0.0041 | 0.0813 | 1.594 |
| M191T229 | β-Damascenone | 1.558e+07 | 2.135e+07 | 1.641e+07 | 7.650e+06 | 7.539e+06 | 6.765e+06 | 2.43 | 1.28 | 0.0011 | 0.0459 | 1.637 |
| M191T259 | Methyl 2-hydroxybenzoate | 1.571e+07 | 2.015e+07 | 1.864e+07 | 1.403e+07 | 1.165e+07 | 9.894e+06 | 1.53 | 0.62 | 0.0259 | 0.1904 | 1.456 |
| M191T348 | 4-Ethyl-2-methoxyphenol | 1.910e+06 | 1.926e+06 | 2.203e+06 | 1.712e+07 | 1.365e+07 | 2.223e+07 | 0.11 | -3.13 | 1.303e-04 | 0.0188 | 1.667 |
| M192T47 | 3-(2-Methylthio)ethylmalate | 4.420e+08 | 3.773e+08 | 3.591e+08 | 2.896e+08 | 2.998e+08 | 2.911e+08 | 1.34 | 0.42 | 0.0106 | 0.1300 | 1.540 |
| M195T94 | (-)-1-(Methylthio)propyl 1-propenyl disulfide | 2.450e+07 | 2.493e+07 | 1.771e+07 | 5.355e+07 | 5.167e+07 | 5.758e+07 | 0.41 | -1.28 | 0.0015 | 0.0514 | 1.630 |
| M197T35 | Deoxyribose 1-phosphate | 2.035e+06 | 1.934e+06 | 2.592e+06 | 7.915e+06 | 5.919e+06 | 5.846e+06 | 0.33 | -1.58 | 0.0012 | 0.0478 | 1.635 |

| ID | Metabolite | MC-P1 | MC-P2 | MC-P3 | MM-P1 | MM-P2 | MM-P3 | FC | $log_2FC$ | P value | FDR | VIP |
|---|---|---|---|---|---|---|---|---|---|---|---|---|
| M199T254_2 | N,N,N-Trimethyl-Histidine | 8.577e+06 | 9.400e+06 | 1.213e+07 | 4.981e+06 | 3.077e+06 | 5.144e+06 | 2.28 | 1.19 | 0.0128 | 0.1402 | 1.527 |
| M205T285 | (3Z,6E)-α-Farnesene | 1.191e+06 | 1.300e+06 | 3.190e+06 | 7.120e+06 | 7.305e+06 | 8.092e+06 | 0.25 | -1.99 | 0.0405 | 0.2386 | 1.547 |
| M205T409 | 5-Oxo-1,2-campholide | 3.499e+07 | 3.459e+07 | 4.139e+07 | 2.472e+07 | 2.399e+07 | 2.561e+07 | 1.49 | 0.58 | 0.0029 | 0.0695 | 1.610 |
| M206T233 | Indole-3-lactic acid | 1.704e+08 | 3.343e+08 | 4.374e+08 | 2.376e+07 | 2.233e+07 | 2.258e+07 | 13.72 | 3.78 | 0.0116 | 0.1364 | 1.644 |
| M211T265 | 3-Amino-9-ethylcarbazole | 8.697e+06 | 1.135e+07 | 1.679e+07 | 6.337e+06 | 6.403e+06 | 5.506e+06 | 2.02 | 1.01 | 0.0275 | 0.1942 | 1.449 |
| M214T222 | N-Benzoyl-4-hydroxyanthranilate | 2.758e+06 | 3.362e+06 | 4.415e+06 | 9.033e+06 | 1.021e+07 | 8.040e+06 | 0.39 | -1.37 | 0.0032 | 0.0731 | 1.605 |
| M219T46 | Germacrone | 6.102e+06 | 6.348e+06 | 7.763e+06 | 1.165e+07 | 1.038e+07 | 1.301e+07 | 0.58 | -0.79 | 0.0051 | 0.0893 | 1.583 |
| M222T203 | 2'-Deoxysepiapterin | 7.969e+06 | 1.151e+07 | 1.429e+07 | 3.040e+06 | 2.748e+06 | 3.249e+06 | 3.74 | 1.90 | 0.0019 | 0.0561 | 1.623 |
| M224T115 | Scoparone | 5.262e+06 | 5.800e+06 | 1.123e+07 | 1.846e+07 | 2.397e+07 | 2.172e+07 | 0.35 | -1.53 | 0.0113 | 0.1349 | 1.535 |
| M224T130 | N-Acetylphinothricin | 3.425e+07 | 2.510e+07 | 7.277e+07 | 4.800e+06 | 4.210e+06 | 4.593e+06 | 9.71 | 3.28 | 0.0193 | 0.1682 | 1.616 |
| M231T222 | 6-Hydroxymelatonin | 1.060e+07 | 9.508e+06 | 1.341e+07 | 2.114e+07 | 2.330e+07 | 1.667e+07 | 0.55 | -0.87 | 0.0134 | 0.1439 | 1.522 |
| M232T75 | S-Acetyldihydrolipoamide | 3.053e+07 | 3.516e+07 | 3.090e+07 | 1.732e+07 | 2.005e+07 | 1.794e+07 | 1.75 | 0.80 | 9.080e-04 | 0.0423 | 1.641 |
| M235T61 | Polygodial | 6.086e+07 | 5.294e+07 | 5.316e+07 | 2.781e+07 | 3.240e+07 | 2.992e+07 | 1.85 | 0.89 | 6.341e-04 | 0.0378 | 1.649 |
| M247T284 | SCHEMBL14765177 | 1.499e+07 | 2.019e+07 | 2.336e+07 | 5.807e+07 | 6.261e+07 | 5.649e+07 | 0.33 | -1.60 | 0.0011 | 0.0459 | 1.638 |
| M247T57_2 | Lacinilene C | 8.289e+06 | 1.125e+07 | 1.482e+07 | 5.352e+06 | 4.473e+06 | 6.652e+06 | 2.09 | 1.06 | 0.0239 | 0.1843 | 1.467 |
| M248T390 | Thiamine | 1.022e+07 | 9.599e+06 | 6.973e+06 | 2.975e+06 | 2.951e+06 | 2.404e+06 | 3.22 | 1.69 | 0.0011 | 0.0459 | 1.638 |
| M248T53 | Isoniazid α-ketoglutaric acid | 1.741e+07 | 2.223e+07 | 1.862e+07 | 1.120e+07 | 9.799e+06 | 1.096e+07 | 1.82 | 0.87 | 0.0021 | 0.0589 | 1.620 |
| M249T220 | 7-Hydroxy-6-(methoxyacetyl)-2,2-dimethyl-2H-1-benzopyran | 3.754e+06 | 4.883e+06 | 4.965e+06 | 6.938e+06 | 5.456e+06 | 7.879e+06 | 0.67 | -0.58 | 0.0483 | 0.2564 | 1.371 |
| M249T248 | Enoximone | 5.786e+06 | 6.793e+06 | 5.715e+06 | 7.346e+06 | 7.636e+06 | 7.642e+06 | 0.81 | -0.31 | 0.0196 | 0.1687 | 1.488 |
| M252T53 | 5'-Deoxyadenosine | 1.768e+07 | 2.425e+07 | 2.375e+07 | 3.576e+06 | 7.951e+06 | 9.433e+06 | 3.13 | 1.65 | 0.0185 | 0.1648 | 1.493 |
| M253T257 | (3xi,5Z)-1,5-Octadien-3-ol | 5.376e+06 | 4.737e+06 | 3.758e+06 | 3.202e+06 | 2.310e+06 | 2.742e+06 | 1.68 | 0.75 | 0.0215 | 0.1746 | 1.478 |
| M253T380 | 3-Methyl-5-pentyl-2-furanpentanoic acid | 1.699e+07 | 1.643e+07 | 1.792e+07 | 1.328e+07 | 1.244e+07 | 1.185e+07 | 1.37 | 0.45 | 0.0017 | 0.0534 | 1.626 |
| M256T388 | 3-Dehydrosphinganine | 2.705e+07 | 2.492e+07 | 1.791e+07 | 6.973e+06 | 4.816e+06 | 5.701e+06 | 4.00 | 2.00 | 0.0011 | 0.0459 | 1.637 |
| M260T54 | Propranolol | 1.460e+08 | 1.740e+08 | 8.833e+07 | 5.749e+07 | 4.343e+07 | 8.323e+07 | 2.22 | 1.15 | 0.0458 | 0.2509 | 1.381 |
| M264T47_2 | 2'-Deoxycytidine-5'-monophosphoric acid | 4.655e+07 | 3.893e+07 | 4.062e+07 | 5.004e+07 | 6.214e+07 | 5.650e+07 | 0.75 | -0.42 | 0.0247 | 0.1873 | 1.462 |
| M265T285 | Artemisinin | 6.357e+05 | 7.000e+05 | 7.260e+05 | 3.816e+06 | 4.705e+06 | 3.835e+06 | 0.17 | -2.58 | 2.329e-05 | 0.0083 | 1.677 |
| M265T47_2 | 3-(6-methylthio)hexylmalate | 1.144e+07 | 1.092e+07 | 9.357e+06 | 6.047e+06 | 7.845e+06 | 5.482e+06 | 1.64 | 0.71 | 0.0152 | 0.1530 | 1.510 |
| M265T73 | Pentalenolactone D | 4.541e+06 | 5.164e+06 | 3.762e+06 | 7.529e+06 | 7.610e+06 | 7.420e+06 | 0.60 | -0.74 | 0.0288 | 0.1983 | 1.588 |
| M266T53 | N-acetylneuraminate | 7.423e+07 | 8.189e+07 | 8.008e+07 | 5.532e+07 | 5.857e+07 | 6.642e+07 | 1.31 | 0.39 | 0.0116 | 0.1364 | 1.534 |
| M273T243 | Luteoforol | 1.220e+07 | 1.050e+07 | 1.307e+07 | 3.888e+06 | 7.862e+06 | 5.162e+06 | 2.12 | 1.08 | 0.0214 | 0.1746 | 1.478 |
| M274T329 | Palmitic acid | 1.143e+08 | 1.032e+08 | 1.156e+08 | 6.903e+07 | 7.450e+07 | 6.523e+07 | 1.60 | 0.67 | 9.024e-04 | 0.0423 | 1.642 |
| M275T268 | Ropivacaine | 1.664e+06 | 5.382e+06 | 2.067e+06 | 1.615e+07 | 1.182e+07 | 9.403e+06 | 0.24 | -2.04 | 0.0179 | 0.1634 | 1.497 |
| M275T294 | 5α-Androstane-3α,17α-diol | 3.598e+07 | 5.376e+07 | 5.083e+07 | 1.207e+08 | 7.824e+07 | 9.612e+07 | 0.48 | -1.07 | 0.0139 | 0.1472 | 1.518 |
| M275T382 | FA 18_4;O | 5.871e+07 | 7.211e+07 | 8.535e+07 | 5.212e+07 | 4.021e+07 | 4.943e+07 | 1.52 | 0.61 | 0.0360 | 0.2224 | 1.416 |
| M277T371 | FA(18_4) | 5.512e+08 | 5.911e+08 | 6.076e+08 | 4.438e+08 | 3.164e+08 | 4.029e+08 | 1.50 | 0.59 | 0.0163 | 0.1586 | 1.505 |
| M278T427 | Benznidazole | 1.931e+07 | 2.618e+07 | 2.521e+07 | 1.560e+07 | 1.299e+07 | 1.487e+07 | 1.63 | 0.70 | 0.0121 | 0.1382 | 1.530 |
| M279T409 | Dibutyl phthalate | 7.800e+07 | 7.341e+07 | 8.176e+07 | 5.437e+07 | 4.684e+07 | 5.020e+07 | 1.54 | 0.62 | 0.0012 | 0.0478 | 1.635 |
| M291T325 | FA 20_2 | 3.679e+07 | 4.815e+07 | 4.340e+07 | 2.474e+07 | 1.947e+07 | 2.482e+07 | 1.86 | 0.89 | 0.0052 | 0.0909 | 1.583 |
| M291T85 | Leucopelargonidin | 2.664e+07 | 2.009e+07 | 2.707e+07 | 1.374e+07 | 1.236e+07 | 6.648e+06 | 2.25 | 1.17 | 0.0260 | 0.1904 | 1.456 |
| M293T294 | ACMC-20m5rp | 2.768e+07 | 3.720e+07 | 3.763e+07 | 7.731e+07 | 4.944e+07 | 5.939e+07 | 0.55 | -0.86 | 0.0229 | 0.1813 | 1.471 |
| M298T420 | Albendazole sulfone | 6.012e+07 | 5.547e+07 | 6.767e+07 | 3.302e+07 | 1.388e+07 | 3.515e+07 | 2.23 | 1.16 | 0.0450 | 0.2491 | 1.385 |

| ID | Metabolite | MC-P1 | MC-P2 | MC-P3 | MM-P1 | MM-P2 | MM-P3 | FC | $log_2FC$ | P value | FDR | VIP |
|---|---|---|---|---|---|---|---|---|---|---|---|---|
| M298T60 | Pisatin | 1.804e+08 | 1.310e+08 | 1.121e+08 | 8.484e+07 | 5.221e+07 | 4.797e+07 | 2.29 | 1.20 | 0.0205 | 0.1714 | 1.483 |
| M298T85 | 6-(α-D-glucosaminyl)-1D-myo-inositol | 9.584e+06 | 8.597e+06 | 7.819e+06 | 2.730e+06 | 3.212e+06 | 4.262e+06 | 2.55 | 1.35 | 0.0027 | 0.0663 | 1.613 |
| M301T251 | Sophorol | 9.265e+06 | 6.751e+06 | 1.059e+06 | 4.950e+07 | 2.299e+07 | 4.124e+07 | 0.15 | -2.74 | 0.0376 | 0.2294 | 1.409 |
| M302T362 | Noroxycodone | 1.288e+07 | 1.467e+07 | 1.539e+07 | 9.511e+06 | 9.631e+06 | 1.089e+07 | 1.43 | 0.52 | 0.0065 | 0.1004 | 1.572 |
| M305T226 | (+)-Taxifolin | 8.949e+06 | 9.085e+06 | 1.360e+07 | 1.086e+06 | 4.382e+06 | 1.284e+06 | 4.69 | 2.23 | 0.0197 | 0.1687 | 1.485 |
| M307T55_1 | (−)-Epigallocatechin | 4.410e+07 | 5.141e+07 | 3.524e+07 | 2.441e+07 | 1.958e+07 | 2.630e+07 | 1.86 | 0.90 | 0.0119 | 0.1372 | 1.531 |
| M307T70 | Leucocyanidin | 1.094e+07 | 2.088e+07 | 1.301e+07 | 3.103e+06 | 4.244e+06 | 3.309e+06 | 4.21 | 2.07 | 0.0028 | 0.0695 | 1.609 |
| M317T229 | 3,4-Dihydroxy-9,10-secoandrosta-1,3,5(10)-triene-9,17-dione | 1.239e+07 | 6.691e+06 | 4.152e+06 | 2.951e+07 | 4.150e+07 | 2.746e+07 | 0.24 | -2.08 | 0.0110 | 0.1330 | 1.537 |
| M318T52 | N-acetylglucosaminylasparagine | 2.830e+07 | 3.809e+07 | 2.559e+07 | 8.888e+07 | 5.301e+07 | 7.785e+07 | 0.42 | -1.26 | 0.0116 | 0.1364 | 1.533 |
| M329T218 | Desulfosinalbin | 2.471e+07 | 2.216e+07 | 2.610e+07 | 3.910e+07 | 3.639e+07 | 4.658e+07 | 0.60 | -0.74 | 0.0043 | 0.0827 | 1.591 |
| M329T251 | Aflatoxin M1 | 9.862e+05 | 3.953e+06 | 6.219e+05 | 2.706e+07 | 1.659e+07 | 3.268e+07 | 0.07 | -3.78 | 0.0080 | 0.1099 | 1.559 |
| M329T424 | Reticulinylium | 1.989e+07 | 1.953e+07 | 2.417e+07 | 1.760e+07 | 1.416e+07 | 1.685e+07 | 1.31 | 0.39 | 0.0480 | 0.2557 | 1.374 |
| M330T237 | Glutathione | 9.969e+06 | 7.082e+06 | 5.826e+06 | 1.047e+07 | 1.326e+07 | 1.458e+07 | 0.60 | -0.74 | 0.0455 | 0.2503 | 1.380 |
| M330T57 | 3-O-β-D-galactopyranosyl-L-arabinose | 4.855e+07 | 4.436e+07 | 2.635e+07 | 2.157e+07 | 1.256e+07 | 1.577e+07 | 2.39 | 1.26 | 0.0250 | 0.1889 | 1.461 |
| M331T218 | 3,7-Dimethylquercetin | 7.946e+08 | 5.815e+08 | 5.081e+08 | 1.194e+09 | 1.205e+09 | 1.157e+09 | 0.53 | -0.92 | 0.0378 | 0.2294 | 1.559 |
| M331T251_1 | (−)-Bisdechlorogeodin | 1.108e+08 | 7.971e+07 | 4.535e+07 | 6.036e+08 | 5.387e+08 | 6.174e+08 | 0.13 | -2.90 | 0.0014 | 0.0507 | 1.631 |
| M334T365 | Perilloside C | 2.199e+07 | 2.154e+07 | 3.583e+07 | 7.796e+05 | 9.363e+06 | 4.261e+06 | 5.51 | 2.46 | 0.0492 | 0.2589 | 1.368 |
| M337T357 | 10S-HpOME | 3.016e+06 | 3.068e+06 | 3.080e+06 | 6.924e+06 | 4.795e+06 | 6.368e+06 | 0.51 | -0.98 | 0.0263 | 0.1907 | 1.596 |
| M337T421 | 10,11-dihydro-12-oxo-LTB4 | 2.838e+07 | 3.180e+07 | 1.390e+08 | 4.098e+08 | 2.334e+08 | 5.082e+08 | 0.17 | -2.53 | 0.0241 | 0.1850 | 1.464 |
| M344T192 | trans-p-Coumaric acid 4-glucoside | 3.105e+07 | 2.864e+07 | 2.593e+07 | 7.069e+07 | 6.407e+07 | 7.521e+07 | 0.41 | -1.29 | 2.129e-04 | 0.0227 | 1.663 |
| M349T181 | Inosine 5'-monophosphate (IMP) | 1.053e+07 | 1.011e+07 | 1.188e+07 | 4.157e+06 | 2.494e+06 | 2.077e+06 | 3.73 | 1.90 | 0.0031 | 0.0724 | 1.606 |
| M351T294 | 3′-O-Methyl-(-)-epicatechin-7-O-sulphate | 9.041e+06 | 1.285e+07 | 1.259e+07 | 3.399e+07 | 1.981e+07 | 2.500e+07 | 0.44 | -1.19 | 0.0136 | 0.1453 | 1.520 |
| M367T112 | 4-O-β-D-mannopyranosyl-N-acetyl-D-glucosamine | 7.690e+06 | 1.083e+07 | 9.476e+06 | 3.516e+07 | 2.593e+07 | 5.184e+07 | 0.25 | -2.01 | 0.0037 | 0.0782 | 1.599 |
| M369T395 | Lathosterol | 1.227e+07 | 1.163e+07 | 9.874e+06 | 4.875e+06 | 1.451e+06 | 2.155e+06 | 3.98 | 1.99 | 0.0141 | 0.1481 | 1.517 |
| M369T423 | Cholesterol | 7.098e+06 | 1.093e+07 | 1.900e+07 | 1.344e+06 | 1.219e+06 | 4.793e+06 | 5.03 | 2.33 | 0.0293 | 0.2004 | 1.444 |
| M370T57 | Arbutin-6P | 1.169e+08 | 1.153e+08 | 1.252e+08 | 1.027e+08 | 7.833e+07 | 6.406e+07 | 1.46 | 0.54 | 0.0467 | 0.2516 | 1.376 |
| M397T416 | ST 29_1;O | 3.607e+07 | 1.752e+07 | 5.363e+07 | 2.254e+08 | 3.923e+08 | 1.749e+08 | 0.14 | -2.89 | 0.0073 | 0.1046 | 1.565 |
| M403T424_1 | Trandolaprilat | 7.211e+07 | 7.416e+07 | 8.495e+07 | 6.103e+07 | 5.518e+07 | 5.603e+07 | 1.34 | 0.42 | 0.0079 | 0.1099 | 1.560 |
| M407T71 | Shanzhiside methyl ester | 4.058e+06 | 1.881e+06 | 1.668e+06 | 5.818e+06 | 1.228e+07 | 6.953e+06 | 0.30 | -1.72 | 0.0270 | 0.1913 | 1.452 |
| M423T398 | 4α-Methyl-5α-cholest-7-en-3β-ol | 1.995e+07 | 2.311e+07 | 2.389e+07 | 1.545e+07 | 1.209e+07 | 1.196e+07 | 1.70 | 0.76 | 0.0061 | 0.0954 | 1.575 |
| M433T228 | Dehydrochlortetracycline | 4.210e+06 | 2.517e+06 | 1.971e+06 | 1.121e+07 | 9.735e+06 | 1.132e+07 | 0.27 | -1.89 | 0.0040 | 0.0813 | 1.595 |
| M437T354_1 | Kanzonol K | 8.870e+07 | 1.177e+08 | 1.095e+08 | 8.335e+07 | 7.092e+07 | 5.765e+07 | 1.49 | 0.58 | 0.0416 | 0.2424 | 1.393 |
| M441T254 | 8-Demethyltetracenomycin C | 2.163e+07 | 4.129e+07 | 3.505e+07 | 1.136e+07 | 1.717e+07 | 8.413e+06 | 2.65 | 1.41 | 0.0257 | 0.1904 | 1.456 |
| M455T348 | 13′-Carboxy-α-tocotrienol | 1.669e+07 | 1.771e+07 | 1.357e+07 | 1.015e+08 | 6.631e+07 | 9.813e+07 | 0.18 | -2.47 | 4.297e-04 | 0.0313 | 1.654 |
| M455T405 | β-Citraurin | 1.571e+07 | 1.384e+07 | 1.624e+07 | 7.520e+06 | 5.692e+06 | 6.229e+06 | 2.36 | 1.24 | 8.343e-04 | 0.0417 | 1.644 |
| M459T440 | Soyasapogenol B | 1.823e+07 | 1.567e+07 | 1.631e+07 | 9.921e+05 | 9.077e+05 | 1.061e+06 | 16.96 | 4.08 | 1.561e-06 | 0.0037 | 1.682 |
| M460T354_2 | Apstatin | 8.376e+07 | 1.090e+08 | 2.487e+07 | 6.749e+06 | 1.115e+07 | 4.839e+06 | 9.57 | 3.26 | 0.0141 | 0.1481 | 1.516 |
| M465T88 | 3,5,7-Trihydroxy-2-[3-hydroxy-4-[(2S,5S)-3,4,5-trihydroxy-6-(hydroxymethyl)oxan-2-yl]oxyphenyl]chromen-4-one | 3.373e+07 | 1.141e+07 | 9.630e+06 | 9.066e+07 | 1.432e+08 | 5.519e+07 | 0.19 | -2.40 | 0.0216 | 0.1749 | 1.478 |
| M473T348 | 6-Deoxocastasterone | 3.419e+06 | 6.523e+06 | 4.593e+06 | 2.853e+07 | 2.086e+07 | 2.997e+07 | 0.18 | -2.45 | 0.0014 | 0.0507 | 1.631 |

| ID | Metabolite | MC-P1 | MC-P2 | MC-P3 | MM-P1 | MM-P2 | MM-P3 | FC | log$_2$FC | P value | FDR | VIP |
|---|---|---|---|---|---|---|---|---|---|---|---|---|
| M479T427_1 | Iloprost | 1.996e+08 | 1.638e+08 | 3.276e+08 | 4.343e+07 | 4.525e+07 | 6.750e+07 | 4.42 | 2.15 | 0.0042 | 0.0817 | 1.594 |
| M479T427_2 | 2-Methoxy-estradiol-17β 3-glucuronide | 2.698e+07 | 2.229e+07 | 1.941e+07 | 6.127e+06 | 2.573e+06 | 8.760e+06 | 3.93 | 1.98 | 0.0171 | 0.1613 | 1.502 |
| M493T218_2 | Tricin 7-glucoside | 9.978e+09 | 7.841e+09 | 6.171e+09 | 1.464e+10 | 1.381e+10 | 1.349e+10 | 0.57 | -0.81 | 0.0149 | 0.1518 | 1.513 |
| M507T223_1 | Quercetin 3-(6′-malonyl-glucoside) | 2.897e+07 | 1.656e+07 | 9.654e+06 | 6.141e+07 | 8.380e+07 | 6.412e+07 | 0.26 | -1.92 | 0.0128 | 0.1402 | 1.525 |
| M573T361 | Butirosin B | 9.194e+06 | 1.119e+07 | 1.624e+07 | 3.028e+06 | 1.687e+06 | 5.400e+06 | 3.62 | 1.86 | 0.0218 | 0.1762 | 1.478 |
| M577T254 | Aromadendrin | 3.772e+07 | 5.202e+07 | 8.907e+07 | 2.151e+07 | 1.801e+07 | 2.116e+07 | 2.95 | 1.56 | 0.0166 | 0.1595 | 1.503 |
| M579T242_1 | Pelargonin | 4.857e+07 | 2.968e+07 | 3.178e+07 | 9.586e+06 | 1.515e+07 | 4.898e+06 | 3.71 | 1.89 | 0.0186 | 0.1648 | 1.491 |
| M584T447 | 3-Hydroxyechinenone | 2.681e+07 | 2.448e+07 | 2.955e+07 | 1.723e+07 | 1.206e+07 | 1.253e+07 | 1.93 | 0.95 | 0.0060 | 0.0954 | 1.576 |
| M595T231_2 | Flavosativaside | 1.027e+07 | 7.455e+06 | 1.122e+07 | 1.760e+06 | 1.325e+06 | 1.182e+06 | 6.79 | 2.76 | 3.681e-04 | 0.0295 | 1.657 |
| M595T243_1 | Cyanidin 3-sophoroside | 9.071e+06 | 7.532e+06 | 1.210e+07 | 2.398e+07 | 2.234e+07 | 2.148e+07 | 0.42 | -1.24 | 0.0034 | 0.0755 | 1.602 |
| M600T435_2 | ACMC-20dgc9 | 1.356e+08 | 1.299e+08 | 1.469e+08 | 8.136e+07 | 8.542e+07 | 1.023e+08 | 1.53 | 0.62 | 0.0053 | 0.0915 | 1.583 |
| M609T251_3 | Neodiosmin | 1.616e+07 | 4.970e+07 | 9.995e+06 | 2.924e+08 | 2.263e+08 | 4.273e+08 | 0.08 | -3.64 | 0.0059 | 0.0954 | 1.576 |
| M611T239 | Delphinidin 3-sophoroside | 1.700e+07 | 1.745e+07 | 5.803e+06 | 1.395e+08 | 1.831e+08 | 1.349e+08 | 0.09 | -3.51 | 0.0025 | 0.0642 | 1.614 |
| M611T251_1 | Rutin | 6.615e+06 | 5.323e+06 | 6.804e+05 | 5.266e+07 | 2.594e+07 | 5.436e+07 | 0.09 | -3.40 | 0.0247 | 0.1873 | 1.462 |
| M625T268 | Isorhamnetin 3-neohesperoside | 1.416e+06 | 9.231e+05 | 5.617e+05 | 2.308e+07 | 2.503e+06 | 1.913e+07 | 0.06 | -3.95 | 0.0326 | 0.2118 | 1.427 |
| M627T231 | 5'-Phosphoribosyl-N-formylglycinamidine | 3.356e+06 | 1.833e+06 | 7.324e+05 | 9.775e+06 | 1.269e+07 | 8.604e+06 | 0.19 | -2.39 | 0.0163 | 0.1586 | 1.505 |
| M639T250 | Malvidin 3-laminaribioside | 1.246e+09 | 1.072e+09 | 3.977e+08 | 5.741e+09 | 8.171e+09 | 6.168e+09 | 0.14 | -2.89 | 0.0049 | 0.0876 | 1.586 |
| M655T242_1 | Rhamnazin 3-sophoroside | 3.738e+08 | 1.456e+08 | 1.212e+08 | 6.963e+08 | 1.412e+09 | 4.909e+08 | 0.25 | -2.02 | 0.0376 | 0.2294 | 1.410 |
| M655T242_2 | 3-methylthiopropyl-desulfoglucosinolate | 6.125e+08 | 4.324e+08 | 1.988e+08 | 1.820e+09 | 1.437e+09 | 1.683e+09 | 0.25 | -1.99 | 0.0122 | 0.1386 | 1.529 |
| M669T251_1 | 8-Demethyl-8-(2,3,4-O-trimethyl-α-L-rhamnosyl)tetracenomycin C | 2.526e+06 | 4.194e+06 | 8.597e+05 | 1.839e+07 | 1.046e+07 | 1.074e+07 | 0.19 | -2.38 | 0.0227 | 0.1806 | 1.472 |
| M672T448 | PC(14_1(9Z)_15_0) | 1.066e+07 | 1.094e+07 | 1.522e+07 | 7.498e+06 | 7.299e+06 | 7.173e+06 | 1.68 | 0.75 | 0.0467 | 0.2516 | 1.530 |
| M673T208_2 | c-di-GMP | 1.035e+07 | 9.846e+06 | 7.409e+06 | 2.529e+06 | 5.528e+06 | 1.506e+06 | 2.89 | 1.53 | 0.0383 | 0.2312 | 1.405 |
| M679T251_2 | UDP-D-Man(2NAc3NAm)A | 1.016e+06 | 2.653e+06 | 3.765e+05 | 1.052e+07 | 8.021e+06 | 1.064e+07 | 0.14 | -2.85 | 0.0167 | 0.1602 | 1.503 |
| M689T433_3 | PC(14_1(9Z)_P-16_0) | 5.461e+07 | 5.137e+07 | 5.817e+07 | 3.338e+07 | 4.010e+07 | 4.143e+07 | 1.43 | 0.51 | 0.0092 | 0.1160 | 1.551 |
| M731T250_2 | Selenodiglutathione | 3.650e+07 | 2.373e+07 | 2.769e+07 | 8.176e+06 | 1.285e+07 | 9.366e+06 | 2.89 | 1.53 | 0.0044 | 0.0835 | 1.591 |
| M733T431 | PC(18_3(6Z,9Z,12Z)_18_4(6Z,9Z,12Z,15Z)) | 6.994e+07 | 6.739e+07 | 5.712e+07 | 1.625e+07 | 1.602e+07 | 2.916e+07 | 3.17 | 1.66 | 0.0045 | 0.0842 | 1.591 |
| M791T448_1 | PC(16_0_22_5) | 1.034e+07 | 1.138e+07 | 1.451e+07 | 5.274e+06 | 6.083e+06 | 4.194e+06 | 2.33 | 1.22 | 0.0046 | 0.0844 | 1.588 |
| M125T128 | 4-hydroxy-5-methyl-2-methylene-3(2H)-furanone | 4.118e+06 | 1.128e+07 | 2.867e+06 | 1.955e+07 | 2.508e+07 | 6.373e+07 | 0.17 | -2.57 | 0.0289 | 0.1404 | 1.333 |
| M143T56 | 4-Hydroxy-2-oxoglutaric acid | 7.106e+06 | 6.195e+06 | 4.466e+06 | 1.294e+07 | 1.743e+07 | 1.292e+07 | 0.41 | -1.29 | 0.0061 | 0.0598 | 1.440 |
| M147T97 | 7-Methylxanthine | 1.280e+07 | 2.727e+06 | 7.304e+06 | 1.776e+07 | 2.767e+07 | 5.590e+07 | 0.23 | -2.15 | 0.0500 | 0.1879 | 1.259 |
| M149T135 | Phenylglyoxylic acid | 1.358e+07 | 9.469e+06 | 7.873e+06 | 3.531e+07 | 2.768e+07 | 2.034e+07 | 0.37 | -1.43 | 0.0118 | 0.0840 | 1.400 |
| M149T209 | Tartronate semialdehyde | 6.782e+06 | 5.948e+06 | 4.384e+06 | 2.236e+07 | 1.448e+07 | 1.105e+07 | 0.36 | -1.48 | 0.0145 | 0.0917 | 1.387 |
| M149T246 | Homogentisic acid | 1.559e+06 | 1.456e+06 | 1.239e+06 | 1.646e+07 | 9.802e+06 | 5.814e+06 | 0.13 | -2.91 | 0.0033 | 0.0426 | 1.474 |
| M153T320 | Acetol phosphate | 7.214e+07 | 3.599e+07 | 8.692e+06 | 1.540e+06 | 1.014e+06 | 6.964e+06 | 12.27 | 3.62 | 0.0408 | 0.1727 | 1.294 |
| M154T55 | L-Histidine | 5.271e+07 | 9.933e+07 | 6.394e+07 | 1.254e+08 | 1.505e+08 | 1.200e+08 | 0.55 | -0.87 | 0.0333 | 0.1542 | 1.315 |
| M154T80 | (3S,5S)-Carbapenam-3-carboxylate | 1.746e+07 | 2.376e+07 | 1.915e+07 | 6.336e+07 | 7.657e+07 | 6.609e+07 | 0.29 | -1.77 | 3.334e-04 | 0.0141 | 1.529 |
| M164T75 | L-Phenylalanine | 4.262e+07 | 5.101e+07 | 4.877e+07 | 1.026e+08 | 9.070e+07 | 8.436e+07 | 0.51 | -0.96 | 0.0011 | 0.0242 | 1.512 |
| M171T442 | FAL 8_1 | 3.735e+07 | 2.986e+07 | 3.794e+07 | 7.054e+06 | 1.314e+07 | 8.190e+06 | 3.70 | 1.89 | 0.0027 | 0.0390 | 1.488 |
| M173T139 | N-Methyltryptamine | 1.686e+07 | 3.210e+07 | 1.877e+07 | 5.749e+06 | 1.291e+07 | 3.606e+06 | 3.04 | 1.61 | 0.0455 | 0.1803 | 1.281 |
| M204T97 | 5-Acetamidovalerate | 6.553e+06 | 1.011e+07 | 8.744e+06 | 3.588e+06 | 3.705e+06 | 2.309e+06 | 2.65 | 1.40 | 0.0079 | 0.0665 | 1.438 |

| ID | Metabolite | MC-P1 | MC-P2 | MC-P3 | MM-P1 | MM-P2 | MM-P3 | FC | $\log_2$FC | P value | FDR | VIP |
|---|---|---|---|---|---|---|---|---|---|---|---|---|
| M225T132 | D-Psicose | 3.244e+07 | 1.518e+07 | 1.994e+07 | 6.555e+07 | 5.675e+07 | 7.389e+07 | 0.34 | -1.54 | 0.0091 | 0.0737 | 1.425 |
| M245T49 | Psoralen | 1.161e+06 | 7.171e+05 | 5.509e+05 | 1.401e+07 | 7.097e+07 | 7.966e+07 | 0.01 | -6.08 | 0.0026 | 0.0385 | 1.484 |
| M247T41 | 2,3-Diphosphoglyceric acid | 4.780e+06 | 3.958e+06 | 3.142e+06 | 6.739e+06 | 5.753e+06 | 6.646e+06 | 0.62 | -0.69 | 0.0205 | 0.1133 | 1.356 |
| M255T54 | Hamamelose | 1.523e+08 | 1.404e+08 | 1.029e+08 | 2.851e+08 | 2.181e+08 | 2.483e+08 | 0.53 | -0.93 | 0.0103 | 0.0772 | 1.413 |
| M261T53 | Helenalin | 1.114e+07 | 6.548e+06 | 6.807e+06 | 5.600e+05 | 6.665e+05 | 2.765e+06 | 6.14 | 2.62 | 0.0182 | 0.1039 | 1.374 |
| M275T42 | 6-Phosphogluconic acid | 2.368e+08 | 2.914e+08 | 2.312e+08 | 1.315e+08 | 1.845e+08 | 1.488e+08 | 1.63 | 0.71 | 0.0159 | 0.0973 | 1.394 |
| M279T310 | (2S)-2-(2,4-dichlorophenoxy)propanoic acid | 5.906e+07 | 1.826e+07 | 3.080e+07 | 6.045e+06 | 3.873e+06 | 3.894e+06 | 7.83 | 2.97 | 0.0060 | 0.0598 | 1.458 |
| M287T223 | (+)-Gallocatechin | 1.105e+07 | 7.390e+06 | 7.605e+06 | 3.771e+05 | 7.870e+05 | 4.669e+05 | 15.97 | 4.00 | 3.835e-04 | 0.0145 | 1.531 |
| M299T229 | Kaempferide | 1.006e+07 | 4.027e+06 | 1.617e+06 | 7.586e+05 | 4.579e+05 | 2.364e+05 | 10.81 | 3.43 | 0.0237 | 0.1233 | 1.368 |
| M299T85 | Methylnissolin | 9.718e+05 | 3.742e+06 | 1.989e+06 | 9.137e+06 | 5.147e+06 | 5.524e+06 | 0.34 | -1.56 | 0.0498 | 0.1876 | 1.268 |
| M301T264 | Quercetin | 1.228e+07 | 1.768e+07 | 8.734e+06 | 1.827e+05 | 4.111e+05 | 2.431e+05 | 46.23 | 5.53 | 2.504e-04 | 0.0139 | 1.534 |
| M309T54 | Flindersiachromone | 4.329e+06 | 4.838e+06 | 3.668e+06 | 9.257e+06 | 9.720e+06 | 7.629e+06 | 0.48 | -1.05 | 0.0026 | 0.0385 | 1.479 |
| M321T50 | Alditol 6-phosphate | 2.332e+07 | 1.400e+07 | 1.232e+07 | 7.660e+06 | 9.446e+05 | 1.029e+06 | 5.15 | 2.37 | 0.0420 | 0.1755 | 1.290 |
| M325T198 | Anordiol | 1.250e+07 | 7.396e+06 | 6.517e+06 | 1.068e+08 | 5.539e+07 | 9.444e+07 | 0.10 | -3.28 | 0.0013 | 0.0266 | 1.503 |
| M329T193 | Tricin | 2.770e+05 | 1.732e+05 | 1.349e+05 | 3.125e+08 | 2.735e+08 | 2.324e+08 | 7.149e-04 | -10.45 | 5.682e-06 | 0.0095 | 1.547 |
| M333T41 | 5,5-Dehydrodivanillate | 1.882e+07 | 1.998e+07 | 1.541e+07 | 1.233e+07 | 1.247e+07 | 1.097e+07 | 1.52 | 0.60 | 0.0097 | 0.0754 | 1.436 |
| M333T47 | 2-(β-D-Glucosyl)-sn-glycerol 3-phosphate | 1.222e+08 | 9.744e+07 | 4.233e+07 | 3.429e+08 | 2.869e+08 | 4.145e+08 | 0.25 | -1.99 | 0.0125 | 0.0858 | 1.399 |
| M339T439 | Behenic acid | 4.710e+06 | 8.913e+06 | 7.301e+06 | 1.455e+07 | 2.819e+07 | 1.760e+07 | 0.35 | -1.53 | 0.0181 | 0.1036 | 1.381 |
| M344T76 | ST 21_3;O5 | 1.489e+07 | 1.103e+07 | 1.654e+07 | 3.812e+07 | 3.362e+07 | 2.681e+07 | 0.43 | -1.21 | 0.0060 | 0.0598 | 1.452 |
| M345T426 | FA 18_0;O | 1.405e+07 | 1.864e+07 | 1.239e+07 | 9.080e+05 | 1.648e+06 | 8.418e+05 | 13.27 | 3.73 | 4.304e-04 | 0.0152 | 1.529 |
| M355T80 | 5-Amino-6-(5'-phosphoribitylamino)uracil | 3.452e+06 | 3.548e+06 | 2.053e+06 | 5.079e+07 | 4.728e+07 | 3.966e+07 | 0.07 | -3.93 | 1.403e-04 | 0.0134 | 1.533 |
| M366T76 | Dehydrohistidyltryptophyldiketopiperazine | 1.375e+08 | 2.174e+08 | 1.716e+08 | 8.841e+07 | 1.228e+07 | 1.032e+07 | 4.74 | 2.25 | 0.0436 | 0.1772 | 1.280 |
| M378T103 | 12-Hydroxychelirubine | 8.103e+06 | 1.006e+07 | 1.171e+07 | 1.288e+06 | 1.393e+06 | 1.498e+06 | 7.15 | 2.84 | 7.111e-05 | 0.0101 | 1.537 |
| M407T197 | Chelirubine | 5.571e+07 | 2.505e+07 | 1.384e+07 | 8.172e+06 | 1.182e+06 | 5.841e+05 | 9.52 | 3.25 | 0.0376 | 0.1652 | 1.310 |
| M409T45 | Xanthylic acid | 1.022e+07 | 9.939e+06 | 4.907e+06 | 2.472e+07 | 1.472e+07 | 2.595e+07 | 0.38 | -1.38 | 0.0312 | 0.1470 | 1.316 |
| M429T69 | Wuweizisu C | 9.892e+06 | 6.212e+06 | 3.348e+06 | 1.255e+07 | 2.064e+07 | 1.826e+07 | 0.38 | -1.40 | 0.0397 | 0.1703 | 1.277 |
| M433T417 | ST 27_1;O4 | 4.466e+07 | 1.911e+07 | 3.335e+07 | 8.666e+06 | 1.845e+07 | 8.018e+06 | 2.76 | 1.47 | 0.0470 | 0.1824 | 1.278 |
| M436T332 | 13-Desoxyterpendole I | 2.030e+07 | 1.836e+07 | 3.529e+06 | 1.767e+06 | 4.892e+05 | 1.962e+05 | 17.21 | 4.10 | 0.0249 | 0.1276 | 1.360 |
| M449T106 | PHC 4-O-glucoside | 1.020e+07 | 4.035e+06 | 5.751e+06 | 2.019e+06 | 1.210e+06 | 2.115e+05 | 5.81 | 2.54 | 0.0498 | 0.1876 | 1.271 |
| M449T220 | 2-Hydroxy-3,6,7,4-tetramethylquercetagetin | 7.080e+06 | 6.234e+06 | 1.127e+07 | 4.635e+04 | 7.778e+05 | 1.860e+05 | 24.34 | 4.61 | 0.0110 | 0.0807 | 1.418 |
| M449T297 | Clinofibrate | 1.552e+07 | 3.054e+07 | 2.436e+07 | 2.803e+05 | 6.926e+05 | 3.554e+05 | 53.02 | 5.73 | 2.826e-04 | 0.0139 | 1.530 |
| M455T396 | Betulinic acid | 6.075e+07 | 1.008e+08 | 8.334e+07 | 4.413e+07 | 2.230e+07 | 2.550e+07 | 2.66 | 1.41 | 0.0172 | 0.1003 | 1.378 |
| M463T425 | Bracteatin 6-glucoside | 5.350e+08 | 5.997e+08 | 3.911e+08 | 1.356e+07 | 1.384e+08 | 1.910e+07 | 8.92 | 3.16 | 0.0208 | 0.1133 | 1.373 |
| M479T228 | THC 4-glucoside | 7.910e+06 | 2.216e+06 | 1.975e+06 | 5.156e+05 | 7.764e+05 | 5.003e+05 | 6.75 | 2.76 | 0.0212 | 0.1144 | 1.378 |
| M491T208 | Sissotrin | 1.703e+08 | 8.272e+07 | 5.550e+07 | 3.133e+06 | 4.785e+06 | 3.657e+06 | 26.65 | 4.74 | 8.109e-04 | 0.0203 | 1.522 |
| M521T52 | Cleistanthin | 1.157e+06 | 8.499e+05 | 5.576e+05 | 3.770e+06 | 1.182e+07 | 8.473e+06 | 0.11 | -3.23 | 0.0055 | 0.0575 | 1.450 |
| M563T417 | 5-O-beta-D-mycaminosyl-20-oxotylonolide | 2.884e+07 | 3.132e+07 | 4.276e+07 | 6.138e+06 | 1.131e+07 | 8.276e+06 | 4.00 | 2.00 | 0.0028 | 0.0394 | 1.482 |
| M567T235 | Trilobolide | 3.108e+07 | 2.938e+07 | 2.780e+07 | 8003 | 2.534e+04 | 1.599e+04 | 1789.31 | 10.81 | 0.0018 | 0.0313 | 1.546 |
| M637T245_2 | Malvin | 4.529e+07 | 2.440e+07 | 6.182e+06 | 1.233e+06 | 3.144e+06 | 1.582e+06 | 12.73 | 3.67 | 0.0230 | 0.1217 | 1.371 |
| M652T250 | Spinacetin 3-gentiobioside | 2.008e+07 | 1.126e+07 | 1.032e+07 | 1.643e+06 | 1.334e+06 | 1.594e+06 | 9.11 | 3.19 | 5.831e-04 | 0.0173 | 1.525 |
| M683T52_2 | Sucrose | 1.512e+07 | 2.198e+07 | 7.827e+06 | 2.529e+07 | 7.821e+07 | 8.308e+07 | 0.24 | -2.05 | 0.0480 | 0.1838 | 1.260 |

| ID | Metabolite | MC-P1 | MC-P2 | MC-P3 | MM-P1 | MM-P2 | MM-P3 | FC | $log_2FC$ | P value | FDR | VIP |
|---|---|---|---|---|---|---|---|---|---|---|---|---|
| M702T418_2 | Erythromycin D | 1.020e+08 | 6.099e+07 | 6.296e+07 | 2.662e+07 | 3.040e+07 | 3.315e+07 | 2.51 | 1.33 | 0.0074 | 0.0636 | 1.449 |
| M703T421_2 | 20-Hydroxy-leukotriene B4 | 3.820e+08 | 3.999e+08 | 3.064e+08 | 5.804e+07 | 8.521e+07 | 7.574e+07 | 4.97 | 2.31 | 3.289e-04 | 0.0141 | 1.532 |
| M707T429 | Uvaricin | 8.264e+07 | 8.202e+07 | 7.526e+07 | 1.510e+07 | 2.782e+07 | 1.460e+07 | 4.17 | 2.06 | 0.0180 | 0.1036 | 1.497 |
| M725T416 | (3S,2S)-4-Ketomyxol 2-α-L-fucoside | 6.056e+07 | 6.740e+07 | 4.540e+07 | 8.869e+06 | 1.673e+07 | 9.359e+06 | 4.96 | 2.31 | 0.0023 | 0.0356 | 1.497 |
| M743T426 | 8,8a-Deoxyoleandolide | 8.707e+07 | 6.856e+07 | 9.301e+07 | 1.210e+07 | 3.455e+07 | 3.907e+07 | 2.90 | 1.54 | 0.0374 | 0.1652 | 1.297 |
| M765T433 | Ginsenoside | 1.122e+07 | 1.350e+07 | 5.505e+06 | 2.024e+06 | 1.542e+06 | 1.103e+06 | 6.47 | 2.69 | 0.0049 | 0.0530 | 1.471 |
| M789T424_2 | (2R,5E)-6,10-dimethyl-2-[(2S)-4-methyl-5-oxido-1,2-dihydrofuro[2,3-c]quinolin-5-ium-2-yl]undeca-5,9-dien-2-ol | 8.283e+06 | 2.933e+06 | 6.084e+06 | 1.678e+06 | 3.721e+05 | 5.703e+05 | 6.60 | 2.72 | 0.0209 | 0.1133 | 1.366 |
| M791T434 | Cholesterol cholate | 9.491e+07 | 6.580e+07 | 5.906e+07 | 1.487e+07 | 1.029e+07 | 2.855e+06 | 7.84 | 2.97 | 0.0125 | 0.0858 | 1.417 |
| M805T407 | Tulathromycin | 4.627e+07 | 4.109e+07 | 4.162e+07 | 1.482e+07 | 2.502e+07 | 1.814e+07 | 2.22 | 1.15 | 0.0064 | 0.0606 | 1.455 |
| M837T410_2 | Dehydroxypaxilline | 3.371e+07 | 3.035e+07 | 6.801e+07 | 4.400e+06 | 8.568e+06 | 7.431e+06 | 6.47 | 2.69 | 0.0048 | 0.0530 | 1.457 |
| M850T423_2 | Solanesyl-PP | 1.314e+08 | 1.475e+08 | 1.131e+08 | 4.717e+07 | 9.974e+07 | 5.808e+07 | 1.91 | 0.94 | 0.0423 | 0.1758 | 1.292 |
| M865T225_2 | Procyanidin C1 | 5.871e+07 | 4.080e+07 | 4.283e+07 | 1.749e+06 | 1.155e+07 | 4.729e+06 | 7.90 | 2.98 | 0.0139 | 0.0902 | 1.404 |

Note: Relative abundance values are normalized peak-area signals for the six independent biological samples. FC is defined as MC-P/MM-P. The 236-feature list represents the uncorrected candidate set screened by VIP>1 and raw P<0.05; FDR values are additionally reported for transparency.

## Table S3. Metabolites meeting the stricter statistical criteria (FDR≤0.05 and $|log_2FC|≥1$), with annotation-level information.

| ID | Metabolite | Direction | FC | $log_2FC$ | P value | FDR | VIP | m/z | RT (s) | Ion mode | Library | Current annotation level | Main-text status |
|---|---|---|---|---|---|---|---|---|---|---|---|---|---|
| M130T202 | Triethylenetetramine | MC-P higher | 4.55 | 2.19 | 8.510e-04 | 0.0417 | 1.643 | 130.0862 | 202.20 | pos | MoNA | MSI_level2 | Supplementary only |
| M144T138 | Desethyl atrazine | MM-P higher | 0.37 | -1.44 | 9.265e-04 | 0.0428 | 1.641 | 144.0812 | 138.10 | pos | BioDeepDB | MSI_level3 | Supplementary only |
| M165T58 | 2-Hydroxycinnamic acid | MC-P higher | 4.00 | 2.00 | 2.849e-07 | 0.0014 | 1.682 | 165.0538 | 57.70 | pos | BioDeepDB | MSI_level1 | Included in Fig. 6 |
| M191T229 | β-Damascenone | MC-P higher | 2.43 | 1.28 | 0.0011 | 0.0459 | 1.637 | 191.1435 | 229.30 | pos | BioDeepDB | MSI_level3 | Included in Fig. 6 |
| M191T348 | 4-Ethyl-2-methoxyphenol | MM-P higher | 0.11 | -3.13 | 1.303e-04 | 0.0188 | 1.667 | 191.1800 | 348.30 | pos | mzVault | MSI_level2 | Supplementary only |
| M197T35 | Deoxyribose 1-phosphate | MM-P higher | 0.33 | -1.58 | 0.0012 | 0.0478 | 1.635 | 197.0207 | 34.80 | pos | BioDeepDB | MSI_level3 | Included in Fig. 6 |
| M247T284 | SCHEMBL14765177 | MM-P higher | 0.33 | -1.60 | 0.0011 | 0.0459 | 1.638 | 247.1333 | 284.10 | pos | BioDeepDB | MSI_level1 | Supplementary only |
| M248T390 | Thiamine | MC-P higher | 3.22 | 1.69 | 0.0011 | 0.0459 | 1.638 | 248.1061 | 389.80 | pos | BioDeepDB | MSI_level3 | Included in Fig. 6 |
| M256T388 | 3-Dehydrosphinganine | MC-P higher | 4.00 | 2.00 | 0.0011 | 0.0459 | 1.637 | 256.3003 | 388.20 | pos | BioDeepDB | MSI_level3 | Included in Fig. 6 |
| M265T285 | Artemisinin | MM-P higher | 0.17 | -2.58 | 2.329e-05 | 0.0083 | 1.677 | 265.1436 | 284.60 | pos | MoNA | MSI_level2 | Supplementary only |
| M344T192 | trans-p-Coumaric acid 4-glucoside | MM-P higher | 0.41 | -1.29 | 2.129e-04 | 0.0227 | 1.663 | 344.1354 | 192.30 | pos | BioDeepDB | MSI_level1 | Included in Fig. 6 |
| M455T348 | 13′-Carboxy-α-tocotrienol | MM-P higher | 0.18 | -2.47 | 4.297e-04 | 0.0313 | 1.654 | 455.3504 | 347.60 | pos | mzVault | MSI_level2 | Supplementary only |
| M455T405 | β-Citraurin | MC-P higher | 2.36 | 1.24 | 8.343e-04 | 0.0417 | 1.644 | 455.2970 | 405.00 | pos | BioDeepDB | MSI_level3 | Supplementary only |
| M459T440 | Soyasapogenol B | MC-P higher | 16.96 | 4.08 | 1.561e-06 | 0.0037 | 1.682 | 459.3845 | 440.30 | pos | BioDeepDB | MSI_level1 | Supplementary only |
| M595T231_2 | Flavosativaside | MC-P higher | 6.79 | 2.76 | 3.681e-04 | 0.0295 | 1.657 | 595.1509 | 231.00 | pos | BioDeepDB | MSI_level1 | Included in Fig. 6 |
| M149T246 | Homogentisic acid | MM-P higher | 0.13 | -2.91 | 0.0033 | 0.0426 | 1.474 | 149.0445 | 246.40 | neg | BioDeepDB | MSI_level1 | Included in Fig. 6 |
| M154T80 | (3S,5S)-Carbapenam-3-carboxylate | MM-P higher | 0.29 | -1.77 | 3.334e-04 | 0.0141 | 1.529 | 154.0610 | 80.40 | neg | BioDeepDB | MSI_level1 | Supplementary only |
| M171T442 | FAL 8_1 | MC-P higher | 3.70 | 1.89 | 0.0027 | 0.0390 | 1.488 | 171.1015 | 442.30 | neg | BioDeepDB | MSI_level1 | Included in Fig. 6 |
| M245T49 | Psoralen | MM-P higher | 0.01 | -6.08 | 0.0026 | 0.0385 | 1.484 | 245.0426 | 48.80 | neg | BioDeepDB | MSI_level3 | Supplementary only |
| M287T223 | (+)-Gallocatechin | MC-P higher | 15.97 | 4.00 | 3.835e-04 | 0.0145 | 1.531 | 287.0555 | 223.30 | neg | BioDeepDB | MSI_level1 | Included in Fig. 6 |
| M301T264 | Quercetin | MC-P higher | 46.23 | 5.53 | 2.504e-04 | 0.0139 | 1.534 | 301.0347 | 263.80 | neg | BioDeepDB | MSI_level1 | Supplementary only |
| M309T54 | Flindersiachromone | MM-P higher | 0.48 | -1.05 | 0.0026 | 0.0385 | 1.479 | 309.0826 | 53.60 | neg | mzVault | MSI_level2 | Supplementary only |
| M325T198 | Anordiol | MM-P higher | 0.10 | -3.28 | 0.0013 | 0.0266 | 1.503 | 325.0917 | 197.50 | neg | mzVault | MSI_level2 | Supplementary only |

| ID | Metabolite | Direction | FC | $log_2$FC | P value | FDR | VIP | m/z | RT (s) | Ion mode | Library | Current annotation level | Main-text status |
|---|---|---|---|---|---|---|---|---|---|---|---|---|---|
| M329T193 | Tricin | MM-P higher | 7.149e-04 | -10.45 | 5.682e-06 | 0.0095 | 1.547 | 329.0662 | 192.90 | neg | MoNA | MSI_level2 | Included in Fig. 6 |
| M345T426 | FA 18_0;O | MC-P higher | 13.27 | 3.73 | 4.304e-04 | 0.0152 | 1.529 | 345.2640 | 426.30 | neg | BioDeepDB | MSI_level1 | Included in Fig. 6 |
| M355T80 | 5-Amino-6-(5'-phosphoribitylamino)uracil | MM-P higher | 0.07 | -3.93 | 1.403e-04 | 0.0134 | 1.533 | 355.0649 | 80.20 | neg | BioDeepDB | MSI_level3 | Included in Fig. 6 |
| M378T103 | 12-Hydroxychelirubine | MC-P higher | 7.15 | 2.84 | 7.111e-05 | 0.0101 | 1.537 | 378.0954 | 103.10 | neg | BioDeepDB | MSI_level1 | Supplementary only |
| M449T297 | Clinofibrate | MC-P higher | 53.02 | 5.73 | 2.826e-04 | 0.0139 | 1.530 | 449.2307 | 297.40 | neg | mzVault | MSI_level2 | Supplementary only |
| M491T208 | Sissotrin | MC-P higher | 26.65 | 4.74 | 8.109e-04 | 0.0203 | 1.522 | 491.1184 | 207.50 | neg | BioDeepDB | MSI_level1 | Supplementary only |
| M563T417 | 5-O-beta-D-mycaminosyl-20-oxotylonolide | MC-P higher | 4.00 | 2.00 | 0.0028 | 0.0394 | 1.482 | 563.3325 | 416.50 | neg | mzVault | MSI_level2 | Supplementary only |
| M567T235 | Trilobolide | MC-P higher | 1789.31 | 10.81 | 0.0018 | 0.0313 | 1.546 | 567.2053 | 235.30 | neg | mzVault | MSI_level2 | Supplementary only |
| M652T250 | Spinacetin 3-gentiobioside | MC-P higher | 9.11 | 3.19 | 5.831e-04 | 0.0173 | 1.525 | 652.1413 | 250.20 | neg | mzVault | MSI_level2 | Supplementary only |
| M703T421_2 | 20-Hydroxy-leukotriene B4 | MC-P higher | 4.97 | 2.31 | 3.289e-04 | 0.0141 | 1.532 | 703.4477 | 420.80 | neg | BioDeepDB | MSI_level1 | Supplementary only |
| M725T416 | (3S,2S)-4-Ketomyxol 2-α-L-fucoside | MC-P higher | 4.96 | 2.31 | 0.0023 | 0.0356 | 1.497 | 725.4468 | 416.50 | neg | BioDeepDB | MSI_level1 | Supplementary only |

## Table S4. The 13 reviewed differential metabolite features displayed in the Figure 6 heatmap.

| Metabolite | MC-P1 | MC-P2 | MC-P3 | MM-P1 | MM-P2 | MM-P3 | $log_2$FC | FDR | VIP | Library | Current annotation level |
|---|---|---|---|---|---|---|---|---|---|---|---|
| 2-Hydroxycinnamic acid | 2.119e+07 | 2.151e+07 | 2.210e+07 | 5.385e+06 | 5.563e+06 | 5.257e+06 | 2.00 | 0.0014 | 1.682 | BioDeepDB | MSI_level1 |
| trans-p-Coumaric acid 4-glucoside | 3.105e+07 | 2.864e+07 | 2.593e+07 | 7.069e+07 | 6.407e+07 | 7.521e+07 | -1.29 | 0.0227 | 1.663 | BioDeepDB | MSI_level1 |
| (+)-Gallocatechin | 1.105e+07 | 7.390e+06 | 7.605e+06 | 3.771e+05 | 7.870e+05 | 4.669e+05 | 4.00 | 0.0145 | 1.531 | BioDeepDB | MSI_level1 |
| Flavosativaside | 1.027e+07 | 7.455e+06 | 1.122e+07 | 1.760e+06 | 1.325e+06 | 1.182e+06 | 2.76 | 0.0295 | 1.657 | BioDeepDB | MSI_level1 |
| Thiamine | 1.022e+07 | 9.599e+06 | 6.973e+06 | 2.975e+06 | 2.951e+06 | 2.404e+06 | 1.69 | 0.0459 | 1.638 | BioDeepDB | MSI_level3 |
| 3-Dehydrosphinganine | 2.705e+07 | 2.492e+07 | 1.791e+07 | 6.973e+06 | 4.816e+06 | 5.701e+06 | 2.00 | 0.0459 | 1.637 | BioDeepDB | MSI_level3 |
| β-Damascenone | 1.558e+07 | 2.135e+07 | 1.641e+07 | 7.650e+06 | 7.539e+06 | 6.765e+06 | 1.28 | 0.0459 | 1.637 | BioDeepDB | MSI_level3 |
| Deoxyribose 1-phosphate | 2.035e+06 | 1.934e+06 | 2.592e+06 | 7.915e+06 | 5.919e+06 | 5.846e+06 | -1.58 | 0.0478 | 1.635 | BioDeepDB | MSI_level3 |
| Tricin | 2.770e+05 | 1.732e+05 | 1.349e+05 | 3.125e+08 | 2.735e+08 | 2.324e+08 | -10.45 | 0.0095 | 1.547 | MoNA | MSI_level2 |
| 5-Amino-6-(5'-phosphoribitylamino)uracil | 3.452e+06 | 3.548e+06 | 2.053e+06 | 5.079e+07 | 4.728e+07 | 3.966e+07 | -3.93 | 0.0134 | 1.533 | BioDeepDB | MSI_level3 |
| FA 18_0;O | 1.405e+07 | 1.864e+07 | 1.239e+07 | 9.080e+05 | 1.648e+06 | 8.418e+05 | 3.73 | 0.0152 | 1.529 | BioDeepDB | MSI_level1 |
| FAL 8_1 | 3.735e+07 | 2.986e+07 | 3.794e+07 | 7.054e+06 | 1.314e+07 | 8.190e+06 | 1.89 | 0.0390 | 1.488 | BioDeepDB | MSI_level1 |
| Homogentisic acid | 1.559e+06 | 1.456e+06 | 1.239e+06 | 1.646e+07 | 9.802e+06 | 5.814e+06 | -2.91 | 0.0426 | 1.474 | BioDeepDB | MSI_level1 |

Note: These 13 features were selected from the 34-feature stricter statistical set after review of annotation evidence and biological plausibility in grape berries.

## Table S5. Representative differential metabolite features emphasized in Figure 7 and the main biological interpretation.

| Metabolite | Higher group | FC (MC-P/MM-P) | $log_2$FC | P value | FDR | VIP | m/z | RT (s) | Ion mode | Adduct | Library | Current annotation level | Interpretation |
|---|---|---|---|---|---|---|---|---|---|---|---|---|---|
| 2-Hydroxycinnamic acid | MC-P | 4.00 | 2.00 | 2.849e-07 | 0.0014 | 1.682 | 165.0538 | 57.70 | pos | [M+H]+ | BioDeepDB | MSI_level1 | Representative feature |
| (+)-Gallocatechin | MC-P | 15.97 | 4.00 | 3.835e-04 | 0.0145 | 1.531 | 287.0555 | 223.30 | neg | [M-H2O-H]- | BioDeepDB | MSI_level1 | Representative feature |
| trans-p-Coumaric acid 4-glucoside | MM-P | 0.41 | -1.29 | 2.129e-04 | 0.0227 | 1.663 | 344.1354 | 192.30 | pos | [M+NH4]+ | BioDeepDB | MSI_level1 | Representative feature |
| Tricin | MM-P | 7.149e-04 | -10.45 | 5.682e-06 | 0.0095 | 1.547 | 329.0662 | 192.90 | neg | [M-H]- | MoNA | MSI_level2 | Putative annotation; cautious interpretation |

**Table S6. Statistical results of KEGG pathway enrichment and topology analysis.**

| Pathway ID | Pathway name | Total | Hits | Up_hits | Down_hits | Raw P value | −log10(P) | FDR | Impact | DA-score | Compound IDs | Compound names |
|---|---|---|---|---|---|---|---|---|---|---|---|---|
| ko00941 | Flavonoid biosynthesis | 74 | 11 | 11 | 0 | 6.592e-06 | 5.181 | 8.108e-04 | 0.1907 | 1 | C00389; C00974; C01617; C03648; C05906; C05907; C12127; C12136; C16407; C16408; C16410 | Quercetin; Aromadendrin; (+)-Taxifolin; Leucopelargonidin; Leucocyanidin; Luteoforol; (+)-Gallocatechin; (−)-Epigallocatechin; THC 4-glucoside; PHC 4-O-glucoside; Bracteatin 6-glucoside |
| ko00944 | Flavone and flavonol biosynthesis | 51 | 6 | 3 | 3 | 0.0031 | 2.513 | 0.1889 | 0.2938 | 0 | C00389; C01265; C04024; C05625; C10098; C12638 | Quercetin; 3,7-Dimethylquercetin; Flavosativaside; Rutin; Kaempferide; Quercetin 3-(6′-malonyl-glucoside) |
| ko00966 | Glucosinolate biosynthesis | 77 | 7 | 3 | 4 | 0.0061 | 2.214 | 0.2506 | 0.0735 | -0.143 | C00079; C00141; C17212; C17227; C17240; C17244; C17245 | L-Phenylalanine; α-Ketoisovaleric acid; 3-(2-Methylthio)ethylmalate; 3-(6-methylthio)hexylmalate; Desulfosinalbin; 3-methylthiopropyl-desulfoglucosinolate; 5-Methylthiopentanaldoxime |
| ko00946 | Degradation of flavonoids | 40 | 4 | 3 | 1 | 0.0263 | 1.581 | 0.5843 | 0.0203 | 0.5 | C00389; C00974; C01617; C05625 | Quercetin; Aromadendrin; (+)-Taxifolin; Rutin |
| ko04112 | Cell cycle - Caulobacter | 1 | 1 | 1 | 0 | 0.0285 | 1.545 | 0.5843 | 1.0000 | 1 | C16463 | c-di-GMP |
| ko05217 | Basal cell carcinoma | 1 | 1 | 1 | 0 | 0.0285 | 1.545 | 0.5843 | 1.0000 | 1 | C00187 | Cholesterol |
| ko00360 | Phenylalanine metabolism | 49 | 4 | 1 | 3 | 0.0501 | 1.300 | 0.8785 | 0.0945 | -0.5 | C00079; C01772; C02137; C12621 | L-Phenylalanine; 2-Hydroxycinnamic acid; Phenylglyoxylic acid; m-Coumaric acid |
| ko00010 | Glycolysis / Gluconeogenesis | 31 | 3 | 1 | 2 | 0.0571 | 1.243 | 0.8785 | 0.0178 | -0.333 | C00111; C01159; C06187 | Dihydroxyacetone phosphate; 2,3-Diphosphoglyceric acid; Arbutin-6P |
| ko05230 | Central carbon metabolism in cancer | 37 | 3 | 1 | 2 | 0.0874 | 1.058 | 0.8970 | 0.0811 | -0.333 | C00079; C00135; C00148 | L-Phenylalanine; L-Histidine; L-Proline |
| ko00333 | Prodigiosin biosynthesis | 18 | 2 | 2 | 0 | 0.0918 | 1.037 | 0.8970 | 0.0241 | 1 | C00148; C21138 | L-Proline; FAL 8_1 |
| ko00984 | Steroid degradation | 18 | 2 | 1 | 1 | 0.0918 | 1.037 | 0.8970 | 0.0822 | 0 | C00187; C04793 | Cholesterol; 3,4-Dihydroxy-9,10-secoandrosta-1,3,5(10)-triene-9,17-dione |
| ko00630 | Glyoxylate and dicarboxylate metabolism | 64 | 4 | 1 | 3 | 0.1089 | 0.963 | 0.8970 | 0.0247 | -0.5 | C00111; C00988; C01127; C01146 | Dihydroxyacetone phosphate; Phosphoglycolic acid; 4-Hydroxy-2-oxoglutaric acid; Tartronate semialdehyde |
| ko00240 | Pyrimidine | 64 | 4 | 0 | 4 | 0.1089 | 0.963 | 0.8970 | 0.0843 | -1 | C00239; C00672; | 2'-Deoxycytidine- |

| Pathway ID | Pathway name | Total | Hits | Up_hits | Down_hits | Raw P value | −log10(P) | FDR | Impact | DA-score | Compound IDs | Compound names |
|---|---|---|---|---|---|---|---|---|---|---|---|---|
| | metabolism | | | | | | | | | | C01563; C20231 | 5'-monophosphoric acid; Deoxyribose 1-phosphate; Carbamic acid; Ureidoperacrylic acid |
| ko00470 | D-Amino acid metabolism | 69 | 4 | 2 | 2 | 0.1333 | 0.875 | 0.8970 | 0.0157 | 0 | C00079; C00135; C00148; C00624 | L-Phenylalanine; L-Histidine; L-Proline; N-Acetylglutamate |
| ko00791 | Atrazine degradation | 23 | 2 | 0 | 2 | 0.1386 | 0.858 | 0.8970 | 0.0786 | -1 | C06559; C08733 | Desethyl atrazine; Cyanurodiamide |
| ko04974 | Protein digestion and absorption | 47 | 3 | 1 | 2 | 0.1493 | 0.826 | 0.8970 | 0.0638 | -0.333 | C00079; C00135; C00148 | L-Phenylalanine; L-Histidine; L-Proline |
| ko00404 | Staurosporine biosynthesis | 48 | 3 | 2 | 1 | 0.1561 | 0.807 | 0.8970 | 0.0243 | 0.333 | C00135; C00148; C22164 | L-Histidine; L-Proline; Dehydrohistidyltryptophyldiketopiperazine |
| ko02026 | Biofilm formation - Escherichia coli | 6 | 1 | 1 | 0 | 0.1593 | 0.798 | 0.8970 | 0.1667 | 1 | C16463 | c-di-GMP |
| ko00073 | Cutin, suberine and wax biosynthesis | 27 | 2 | 1 | 1 | 0.1789 | 0.747 | 0.8970 | 0.0488 | 0 | C00249; C08281 | Palmitic acid; Behenic acid |
| ko00970 | Aminoacyl-tRNA biosynthesis | 52 | 3 | 1 | 2 | 0.1842 | 0.735 | 0.8970 | 0.0337 | -0.333 | C00079; C00135; C00148 | L-Phenylalanine; L-Histidine; L-Proline |
| ko04978 | Mineral absorption | 29 | 2 | 1 | 1 | 0.1997 | 0.700 | 0.8970 | 0.0690 | 0 | C00079; C00148 | L-Phenylalanine; L-Proline |
| ko02010 | ABC transporters | 139 | 6 | 2 | 4 | 0.2048 | 0.689 | 0.8970 | 0.0432 | -0.333 | C00051; C00079; C00089; C00135; C00148; C00378 | Glutathione; L-Phenylalanine; Sucrose; L-Histidine; L-Proline; Thiamine |
| ko00760 | Nicotinate and nicotinamide metabolism | 55 | 3 | 1 | 2 | 0.2062 | 0.686 | 0.8970 | 0.0786 | -0.333 | C00111; C04226; C15987 | Dihydroxyacetone phosphate; 1,4,5,6-Tetrahydro-6-oxonicotinic acid; 4-Methylaminobutyrate |
| ko02025 | Biofilm formation - Pseudomonas aeruginosa | 8 | 1 | 1 | 0 | 0.2066 | 0.685 | 0.8970 | 0.1250 | 1 | C16463 | c-di-GMP |
| ko05111 | Biofilm formation - Vibrio cholerae | 8 | 1 | 1 | 0 | 0.2066 | 0.685 | 0.8970 | 0.1250 | 1 | C16463 | c-di-GMP |
| ko00380 | Tryptophan metabolism | 83 | 4 | 1 | 3 | 0.2114 | 0.675 | 0.8970 | 0.0127 | -0.5 | C00637; C05643; C06213; C17203 | Indoleacetaldehyde; 6-Hydroxymelatonin; N-Methyltryptamine; N-hydroxyl-tryptamine |
| ko00310 | Lysine degradation | 56 | 3 | 3 | 0 | 0.2136 | 0.670 | 0.8970 | 0.0012 | 1 | C03087; C03793; C16741 | 5-Acetamidovalerate; N6,N6,N6-trimethyl-L-lysine; 5-Hydroxylysine |
| ko02060 | Phosphotransferase system (PTS) | 57 | 3 | 2 | 1 | 0.2211 | 0.655 | 0.8970 | 0.0526 | 0.333 | C00089; C01096; C06187 | Sucrose; Alditol 6-phosphate; Arbutin-6P |
| ko00332 | Carbapenem biosynthesis | 32 | 2 | 1 | 1 | 0.2313 | 0.636 | 0.8970 | 0.0556 | 0 | C00148; C17367 | L-Proline; (3S,5S)-Carbapenam-3-carboxylate |
| ko04742 | Taste transduction | 33 | 2 | 1 | 1 | 0.2420 | 0.616 | 0.8970 | 0.0606 | 0 | C00089; C00130 | Sucrose; Inosine 5'-monophosphate (IMP) |
| ko00403 | Indole diterpene | 33 | 2 | 2 | 0 | 0.2420 | 0.616 | 0.8970 | 0.1032 | 1 | C20531; C20542 | Dehydroxypaxilline |

| Pathway ID | Pathway name | Total | Hits | Up_hits | Down_hits | Raw P value | −log10(P) | FDR | Impact | DA-score | Compound IDs | Compound names |
|---|---|---|---|---|---|---|---|---|---|---|---|---|
| | alkaloid biosynthesis | | | | | | | | | | | ; 13-Desoxyterpendole I |
| ko04979 | Cholesterol metabolism | 10 | 1 | 1 | 0 | 0.2513 | 0.600 | 0.8970 | 0.1000 | 1 | C00187 | Cholesterol |
| ko00999 | Biosynthesis of various plant secondary metabolites | 149 | 6 | 3 | 3 | 0.2513 | 0.600 | 0.8970 | 0.0034 | 0 | C00079; C01772; C09305; C09311; C20778; C20894 | L-Phenylalanine; 2-Hydroxycinnamic acid; Psoralen; Scoparone; Ginsenoside; β-Citraurin |
| ko00400 | Phenylalanine, tyrosine and tryptophan biosynthesis | 35 | 2 | 0 | 2 | 0.2633 | 0.580 | 0.8970 | 0.0031 | -1 | C00079; C20327 | L-Phenylalanine; 2-Oxo-4-phenylbutyric acid |
| ko04122 | Sulfur relay system | 11 | 1 | 1 | 0 | 0.2726 | 0.564 | 0.8970 | 0.0909 | 1 | C00378 | Thiamine |
| ko00943 | Isoflavonoid biosynthesis | 64 | 3 | 2 | 1 | 0.2749 | 0.561 | 0.8970 | 0.0144 | 0.333 | C05376; C10516; C16227 | Sissotrin; Pisatin; Sophorol |
| ko00030 | Pentose phosphate pathway | 37 | 2 | 1 | 1 | 0.2846 | 0.546 | 0.8970 | 0.0973 | 0 | C00345; C00672 | 6-Phosphogluconic acid; Deoxyribose 1-phosphate |
| ko00942 | Anthocyanin biosynthesis | 66 | 3 | 1 | 2 | 0.2905 | 0.537 | 0.8970 | 0.0663 | -0.333 | C08725; C16306; C16307 | Pelargonin; Cyanidin 3-sophoroside; Delphinidin 3-sophoroside |
| ko04927 | Cortisol synthesis and secretion | 12 | 1 | 1 | 0 | 0.2934 | 0.533 | 0.8970 | 0.0833 | 1 | C00187 | Cholesterol |
| ko04977 | Vitamin digestion and absorption | 39 | 2 | 2 | 0 | 0.3059 | 0.514 | 0.8970 | 0.0513 | 1 | C00187; C00378 | Cholesterol; Thiamine |
| ko05415 | Diabetic cardiomyopathy | 39 | 2 | 1 | 1 | 0.3059 | 0.514 | 0.8970 | 0.0513 | 0 | C00051; C00318 | Glutathione; Carnitine |
| ko04934 | Cushing syndrome | 13 | 1 | 1 | 0 | 0.3136 | 0.504 | 0.8970 | 0.0769 | 1 | C00187 | Cholesterol |
| ko04975 | Fat digestion and absorption | 13 | 1 | 1 | 0 | 0.3136 | 0.504 | 0.8970 | 0.0769 | 1 | C00187 | Cholesterol |
| ko00230 | Purine metabolism | 101 | 4 | 2 | 2 | 0.3250 | 0.488 | 0.8977 | 0.1662 | 0 | C00130; C00242; C00655; C04640 | Inosine 5'-monophosphate (IMP); Guanine; Xanthylic acid; 5'-Phosphoribosyl-N-formylglycinamidine |
| ko05417 | Lipid and atherosclerosis | 14 | 1 | 1 | 0 | 0.3332 | 0.477 | 0.8977 | 0.0714 | 1 | C00187 | Cholesterol |
| ko00592 | alpha-Linolenic acid metabolism | 44 | 2 | 2 | 0 | 0.3584 | 0.446 | 0.8977 | 0.0024 | 1 | C16300; C16320 | FA(18_4); FA 18_4;O |
| ko00996 | Biosynthesis of various alkaloids | 76 | 3 | 2 | 1 | 0.3691 | 0.433 | 0.8977 | 0.0214 | 0.333 | C00079; C00187; C17268 | L-Phenylalanine; Cholesterol; 1-Phenyl-1,2-propanedione |
| ko00052 | Galactose metabolism | 46 | 2 | 0 | 2 | 0.3791 | 0.421 | 0.8977 | 0.0159 | -1 | C00089; C00111 | Sucrose; Dihydroxyacetone phosphate |
| ko00120 | Primary bile acid biosynthesis | 47 | 2 | 2 | 0 | 0.3893 | 0.410 | 0.8977 | 0.0850 | 1 | C00187; C01301 | Cholesterol; ST 27_1;O4 |
| ko00340 | Histidine metabolism | 47 | 2 | 1 | 1 | 0.3893 | 0.410 | 0.8977 | 0.1877 | 0 | C00135; C05575 | L-Histidine; N,N,N-Trimethyl-Histidine |
| ko00910 | Nitrogen metabolism | 19 | 1 | 0 | 1 | 0.4232 | 0.374 | 0.8977 | 0.1412 | -1 | C01563 | Carbamic acid |
| ko00361 | Chlorocyclohexane and chlorobenzene degradation | 83 | 3 | 2 | 1 | 0.4233 | 0.373 | 0.8977 | 0.0592 | 0.333 | C04431; C04729; C05618 | cis-4-Carboxymethylenebut-2-en-4-olide; trans-2-Chlorodienelactone; 3-Chlorocatechol |
| ko00522 | Biosynthesis of 12-, | 87 | 3 | 3 | 0 | 0.4535 | 0.343 | 0.8977 | 0.1090 | 1 | C06633; C11989; | Erythromycin D; |

| Pathway ID | Pathway name | Total | Hits | Up_hits | Down_hits | Raw P value | −log10(P) | FDR | Impact | DA-score | Compound IDs | Compound names |
|---|---|---|---|---|---|---|---|---|---|---|---|---|
| | 14- and 16-membered macrolides | | | | | | | | | | C20760 | 8,8a-Deoxyoleandolide; 5-O-beta-D-mycaminosyl-20-oxotylonolide |
| ko00980 | Metabolism of xenobiotics by cytochrome P450 | 121 | 4 | 0 | 4 | 0.4545 | 0.342 | 0.8977 | 0.0189 | -1 | C03012; C14786; C14789; C14802 | Naphthalene-1,2-diol; Naphthalene epoxide; N-Hydroxy-1-aminonaphthalene; 1-Nitronaphthalene-7,8-oxide |
| ko00710 | Carbon fixation in photosynthetic organisms | 21 | 1 | 0 | 1 | 0.4557 | 0.341 | 0.8977 | 0.0139 | -1 | C00111 | Dihydroxyacetone phosphate |
| ko04918 | Thyroid hormone synthesis | 21 | 1 | 0 | 1 | 0.4557 | 0.341 | 0.8977 | 0.0476 | -1 | C00051 | Glutathione |
| ko00051 | Fructose and mannose metabolism | 55 | 2 | 1 | 1 | 0.4680 | 0.330 | 0.8977 | 0.0796 | 0 | C00111; C01096 | Dihydroxyacetone phosphate; Alditol 6-phosphate |
| ko04925 | Aldosterone synthesis and secretion | 22 | 1 | 1 | 0 | 0.4712 | 0.327 | 0.8977 | 0.0455 | 1 | C00187 | Cholesterol |
| ko00232 | Caffeine metabolism | 22 | 1 | 0 | 1 | 0.4712 | 0.327 | 0.8977 | 0.1289 | -1 | C16353 | 7-Methylxanthine |
| ko00541 | O-Antigen nucleotide sugar biosynthesis | 90 | 3 | 1 | 2 | 0.4758 | 0.323 | 0.8977 | 0.0204 | -0.333 | C00270; C19972; C22246 | N-acetylneuraminate; SCHEMBL14765177; UDP-D-Man(2NAc3NAm)A |
| ko00220 | Arginine biosynthesis | 23 | 1 | 1 | 0 | 0.4863 | 0.313 | 0.8977 | 0.0202 | 1 | C00624 | N-Acetylglutamate |
| ko04152 | AMPK signaling pathway | 23 | 1 | 1 | 0 | 0.4863 | 0.313 | 0.8977 | 0.0435 | 1 | C00389 | Quercetin |
| ko04714 | Thermogenesis | 23 | 1 | 1 | 0 | 0.4863 | 0.313 | 0.8977 | 0.0435 | 1 | C00318 | Carnitine |
| ko00290 | Valine, leucine and isoleucine biosynthesis | 23 | 1 | 1 | 0 | 0.4863 | 0.313 | 0.8977 | 0.0916 | 1 | C00141 | α-Ketoisovaleric acid |
| ko00100 | Steroid biosynthesis | 57 | 2 | 2 | 0 | 0.4867 | 0.313 | 0.8977 | 0.0478 | 1 | C00187; C01189 | Cholesterol; Lathosterol |
| ko00643 | Styrene degradation | 24 | 1 | 0 | 1 | 0.5010 | 0.300 | 0.8977 | 0.0325 | -1 | C00544 | Homogentisic acid |
| ko04913 | Ovarian steroidogenesis | 24 | 1 | 1 | 0 | 0.5010 | 0.300 | 0.8977 | 0.0417 | 1 | C00187 | Cholesterol |
| ko00740 | Riboflavin metabolism | 24 | 1 | 0 | 1 | 0.5010 | 0.300 | 0.8977 | 0.0953 | -1 | C04454 | 5-Amino-6-(5'-phosphoribitylamino)uracil |
| ko00981 | Insect hormone biosynthesis | 25 | 1 | 1 | 0 | 0.5153 | 0.288 | 0.8977 | 0.0032 | 1 | C00187 | Cholesterol |
| ko00950 | Isoquinoline alkaloid biosynthesis | 131 | 4 | 4 | 0 | 0.5166 | 0.287 | 0.8977 | 0.0304 | 1 | C05193; C06167; C06327; C06512 | 12-Hydroxychelirubine; Reticulinylium; Chelirubine; ACMC-20dgc9 |
| ko00140 | Steroid hormone biosynthesis | 99 | 3 | 2 | 1 | 0.5400 | 0.268 | 0.8977 | 0.0147 | 0.333 | C00187; C05469; C11131 | Cholesterol; ST 21_3;O5; 2-Methoxy-estradiol-17β 3-glucuronide |
| ko00254 | Aflatoxin biosynthesis | 27 | 1 | 0 | 1 | 0.5427 | 0.265 | 0.8977 | 0.0007 | -1 | C16755 | Aflatoxin M1 |
| ko00450 | Selenocompound metabolism | 27 | 1 | 1 | 0 | 0.5427 | 0.265 | 0.8977 | 0.0016 | 1 | C18870 | Selenodiglutathione |
| ko04973 | Carbohydrate digestion and absorption | 27 | 1 | 0 | 1 | 0.5427 | 0.265 | 0.8977 | 0.0370 | -1 | C00089 | Sucrose |

| Pathway ID | Pathway name | Total | Hits | Up_hits | Down_hits | Raw P value | −log10(P) | FDR | Impact | DA-score | Compound IDs | Compound names |
|---|---|---|---|---|---|---|---|---|---|---|---|---|
| ko04976 | Bile secretion | 174 | 5 | 4 | 1 | 0.5570 | 0.254 | 0.8977 | 0.0287 | 0.6 | C00051; C00187; C00318; C01405; C06804 | Glutathione; Cholesterol; Carnitine; Aspirin; Acetaminophen |
| ko00905 | Brassinosteroid biosynthesis | 29 | 1 | 0 | 1 | 0.5685 | 0.245 | 0.8977 | 0.0077 | -1 | C15802 | 6-Deoxocastasterone |
| ko00770 | Pantothenate and CoA biosynthesis | 30 | 1 | 1 | 0 | 0.5808 | 0.236 | 0.8977 | 0.0721 | 1 | C00141 | α-Ketoisovaleric acid |
| ko04216 | Ferroptosis | 31 | 1 | 0 | 1 | 0.5928 | 0.227 | 0.8977 | 0.0323 | -1 | C00051 | Glutathione |
| ko05200 | Pathways in cancer | 31 | 1 | 1 | 0 | 0.5928 | 0.227 | 0.8977 | 0.0323 | 1 | C00187 | Cholesterol |
| ko00730 | Thiamine metabolism | 31 | 1 | 1 | 0 | 0.5928 | 0.227 | 0.8977 | 0.0586 | 1 | C00378 | Thiamine |
| ko00401 | Novobiocin biosynthesis | 32 | 1 | 1 | 0 | 0.6045 | 0.219 | 0.8977 | 0.0006 | 1 | C00148 | L-Proline |
| ko00410 | beta-Alanine metabolism | 32 | 1 | 0 | 1 | 0.6045 | 0.219 | 0.8977 | 0.0013 | -1 | C00135 | L-Histidine |
| ko00130 | Ubiquinone and other terpenoid-quinone biosynthesis | 71 | 2 | 1 | 1 | 0.6058 | 0.218 | 0.8977 | 0.0601 | 0 | C00544; C04145 | Homogentisic acid; Solanesyl-PP |
| ko00960 | Tropane, piperidine and pyridine alkaloid biosynthesis | 72 | 2 | 1 | 1 | 0.6134 | 0.212 | 0.8982 | 0.0031 | 0 | C00079; C06366 | L-Phenylalanine; 4,4'-Diaminodibutylamine |
| ko01040 | Biosynthesis of unsaturated fatty acids | 74 | 2 | 1 | 1 | 0.6284 | 0.202 | 0.9060 | 0.0208 | 0 | C00249; C08281 | Palmitic acid; Behenic acid |
| ko00600 | Sphingolipid metabolism | 35 | 1 | 1 | 0 | 0.6375 | 0.196 | 0.9060 | 0.0362 | 1 | C02934 | 3-Dehydrosphinganine |
| ko00350 | Tyrosine metabolism | 78 | 2 | 0 | 2 | 0.6570 | 0.182 | 0.9060 | 0.0559 | -1 | C00544; C05588 | Homogentisic acid; Metanephrine |
| ko00500 | Starch and sucrose metabolism | 37 | 1 | 0 | 1 | 0.6580 | 0.182 | 0.9060 | 0.2016 | -1 | C00089 | Sucrose |
| ko00561 | Glycerolipid metabolism | 38 | 1 | 0 | 1 | 0.6678 | 0.175 | 0.9060 | 0.0006 | -1 | C00111 | Dihydroxyacetone phosphate |
| ko00480 | Glutathione metabolism | 38 | 1 | 0 | 1 | 0.6678 | 0.175 | 0.9060 | 0.2738 | -1 | C00051 | Glutathione |
| ko00062 | Fatty acid elongation | 40 | 1 | 1 | 0 | 0.6866 | 0.163 | 0.9060 | 0.0007 | 1 | C00249 | Palmitic acid |
| ko00640 | Propanoate metabolism | 41 | 1 | 0 | 1 | 0.6955 | 0.158 | 0.9060 | 0.0005 | -1 | C00111 | Dihydroxyacetone phosphate |
| ko00280 | Valine, leucine and isoleucine degradation | 42 | 1 | 1 | 0 | 0.7043 | 0.152 | 0.9060 | 0.0164 | 1 | C00141 | α-Ketoisovaleric acid |
| ko00627 | Aminobenzoate degradation | 86 | 2 | 1 | 1 | 0.7089 | 0.149 | 0.9060 | 0.0467 | 0 | C02137; C18347 | Phenylglyoxylic acid; 5,5-Dehydrodivanillate |
| ko00785 | Lipoic acid metabolism | 44 | 1 | 1 | 0 | 0.7210 | 0.142 | 0.9060 | 0.0009 | 1 | C00141 | α-Ketoisovaleric acid |
| ko00460 | Cyanoamino acid metabolism | 45 | 1 | 0 | 1 | 0.7290 | 0.137 | 0.9060 | 0.0007 | -1 | C00079 | L-Phenylalanine |
| ko00900 | Terpenoid backbone biosynthesis | 46 | 1 | 1 | 0 | 0.7368 | 0.133 | 0.9060 | 0.0006 | 1 | C04145 | Solanesyl-PP |
| ko00562 | Inositol phosphate metabolism | 47 | 1 | 0 | 1 | 0.7443 | 0.128 | 0.9060 | 0.0104 | -1 | C00111 | Dihydroxyacetone phosphate |
| ko00071 | Fatty acid degradation | 50 | 1 | 1 | 0 | 0.7657 | 0.116 | 0.9060 | 0.0006 | 1 | C00249 | Palmitic acid |
| ko00907 | Pinene, camphor and geraniol degradation | 55 | 1 | 1 | 0 | 0.7975 | 0.098 | 0.9060 | 0.0096 | 1 | C21930 | 5-Oxo-1,2-campholide |
| ko02020 | Two-component system | 55 | 1 | 1 | 0 | 0.7975 | 0.098 | 0.9060 | 0.0182 | 1 | C16463 | c-di-GMP |

| Pathway ID | Pathway name | Total | Hits | Up_hits | Down_hits | Raw P value | −log10(P) | FDR | Impact | DA-score | Compound IDs | Compound names |
|---|---|---|---|---|---|---|---|---|---|---|---|---|
| ko02024 | Quorum sensing | 55 | 1 | 1 | 0 | 0.7975 | 0.098 | 0.9060 | 0.0182 | 1 | C16463 | c-di-GMP |
| ko00440 | Phosphonate and phosphinate metabolism | 56 | 1 | 1 | 0 | 0.8033 | 0.095 | 0.9060 | 0.0042 | 1 | C17952 | N-Acetylphinothricin |
| ko00564 | Glycerophospholipid metabolism | 56 | 1 | 0 | 1 | 0.8033 | 0.095 | 0.9060 | 0.0713 | -1 | C00111 | Dihydroxyacetone phosphate |
| ko00053 | Ascorbate and aldarate metabolism | 57 | 1 | 0 | 1 | 0.8090 | 0.092 | 0.9060 | 0.0027 | -1 | C01146 | Tartronate semialdehyde |
| ko05208 | Chemical carcinogenesis - reactive oxygen species | 57 | 1 | 0 | 1 | 0.8090 | 0.092 | 0.9060 | 0.0175 | -1 | C00051 | Glutathione |
| ko00061 | Fatty acid biosynthesis | 58 | 1 | 1 | 0 | 0.8145 | 0.089 | 0.9060 | 0.0002 | 1 | C00249 | Palmitic acid |
| ko00940 | Phenylpropanoid biosynthesis | 58 | 1 | 0 | 1 | 0.8145 | 0.089 | 0.9060 | 0.0002 | -1 | C00079 | L-Phenylalanine |
| ko00624 | Polycyclic aromatic hydrocarbon degradation | 107 | 2 | 0 | 2 | 0.8148 | 0.089 | 0.9060 | 0.0011 | -1 | C03012; C16215 | Naphthalene-1,2-diol; 1,2-Dihydrophthalic acid |
| ko00998 | Biosynthesis of various antibiotics | 108 | 2 | 1 | 1 | 0.8188 | 0.087 | 0.9060 | 0.0142 | 0 | C20399; C21875 | Pentalenolactone D; (2R,5E)-6,10-dimethyl-2-[(2S)-4-methyl-5-oxido-1,2-dihydrofuro[2,3-c]quinolin-5-ium-2-yl]undeca-5,9-dien-2-ol |
| ko00040 | Pentose and glucuronate interconversions | 59 | 1 | 0 | 1 | 0.8198 | 0.086 | 0.9060 | 0.0023 | -1 | C00111 | Dihydroxyacetone phosphate |
| ko00626 | Naphthalene degradation | 60 | 1 | 0 | 1 | 0.8250 | 0.084 | 0.9060 | 0.0650 | -1 | C03012 | Naphthalene-1,2-diol |
| ko00983 | Drug metabolism - other enzymes | 63 | 1 | 1 | 0 | 0.8396 | 0.076 | 0.9078 | 0.0019 | 1 | C16625 | Isoniazid α-ketoglutaric acid |
| ko00520 | Amino sugar and nucleotide sugar metabolism | 118 | 2 | 1 | 1 | 0.8553 | 0.068 | 0.9078 | 0.0176 | 0 | C00270; C19972 | N-acetylneuraminate; SCHEMBL14765177 |
| ko00270 | Cysteine and methionine metabolism | 67 | 1 | 0 | 1 | 0.8573 | 0.067 | 0.9078 | 0.0002 | -1 | C00051 | Glutathione |
| ko00997 | Biosynthesis of various other secondary metabolites | 67 | 1 | 0 | 1 | 0.8573 | 0.067 | 0.9078 | 0.0025 | -1 | C00079 | L-Phenylalanine |
| ko00330 | Arginine and proline metabolism | 69 | 1 | 1 | 0 | 0.8654 | 0.063 | 0.9078 | 0.0866 | 1 | C00148 | L-Proline |
| ko00906 | Carotenoid biosynthesis | 123 | 2 | 2 | 0 | 0.8709 | 0.060 | 0.9078 | 0.0049 | 1 | C15942; C15966 | (3S,2S)-4-Ketomyxol 2-α-L-fucoside; 3-Hydroxyechinenone |
| ko05204 | Chemical carcinogenesis - DNA adducts | 78 | 1 | 0 | 1 | 0.8966 | 0.047 | 0.9164 | 0.0128 | -1 | C03012 | Naphthalene-1,2-diol |
| ko00590 | Arachidonic acid metabolism | 79 | 1 | 1 | 0 | 0.8995 | 0.046 | 0.9164 | 0.0003 | 1 | C04853 | 20-Hydroxy-leukotriene B4 |
| ko00524 | Neomycin, kanamycin and gentamicin biosynthesis | 81 | 1 | 1 | 0 | 0.9053 | 0.043 | 0.9164 | 0.0041 | 1 | C17586 | Butirosin B |
| ko01057 | Biosynthesis of type II polyketide products | 138 | 2 | 1 | 1 | 0.9089 | 0.041 | 0.9164 | 0.0182 | 0 | C12379; C20977 | 8-Demethyltetracenomycin C; 8- |

| Pathway ID | Pathway name | Total | Hits | Up_hits | Down_hits | Raw P value | −log10(P) | FDR | Impact | DA-score | Compound IDs | Compound names |
|---|---|---|---|---|---|---|---|---|---|---|---|---|
| | | | | | | | | | | | | Demethyl-8-(2,3,4-O-trimethyl-α-L-rhamnosyl)tetracenomycin C |
| ko00680 | Methane metabolism | 88 | 1 | 0 | 1 | 0.9228 | 0.035 | 0.9228 | 0.0002 | -1 | C00111 | Dihydroxyacetone phosphate |

Note: DA-score = (Up_hits − Down_hits) / Hits. Positive values indicate a predominance of metabolites with higher relative abundance in Cabernet Sauvignon (MC-P), whereas negative values indicate a predominance in Marselan (MM-P). Raw P values were adjusted using the Benjamini–Hochberg procedure to obtain FDR values. Raw P value, unadjusted pathway-enrichment P value; FDR, Benjamini-Hochberg-adjusted P value; Impact, pathway-topology impact value. The KEGG column contains hyperlinks to the corresponding mapped pathway.
